# A BAHD-type acyltransferase concludes the biosynthetic pathway of non-bitter glycoalkaloids in ripe tomato fruit

**DOI:** 10.1038/s41467-023-40092-5

**Published:** 2023-07-27

**Authors:** Prashant D. Sonawane, Sachin A. Gharat, Adam Jozwiak, Ranjit Barbole, Sarah Heinicke, Efrat Almekias-Siegl, Sagit Meir, Ilana Rogachev, Sarah E. O’ Connor, Ashok P. Giri, Asaph Aharoni

**Affiliations:** 1 Department of Natural Product Biosynthesis, Max Planck Institute for Chemical Ecology, Jena, 07745 Germany; 2 Department of Plant and Environmental Sciences, Weizmann Institute of Science, Rehovot, 7610001 Israel; 3 Biochemical Sciences Division, CSIR-National Chemical Laboratory, Pune, 411008 India; 4 Academy of Scientific and Innovative Research (AcSIR), Ghaziabad, 201002 India

**Keywords:** Molecular engineering in plants, Metabolomics, Secondary metabolism, Plant domestication

## Abstract

Tomato is the highest value fruit and vegetable crop worldwide, yet produces *α*-tomatine, a renowned toxic and bitter-tasting anti-nutritional steroidal glycoalkaloid (SGA) involved in plant defense. A suite of modifications during tomato fruit maturation and ripening converts *α*-tomatine to the non-bitter and less toxic Esculeoside A. This important metabolic shift prevents bitterness and toxicity in ripe tomato fruit. While the enzymes catalyzing glycosylation and hydroxylation reactions in the Esculeoside A pathway have been resolved, the proposed acetylating step remains, to date, elusive. Here, we discovered that GAME36 (GLYCOALKALOID METABOLISM36), a BAHD-type acyltransferase catalyzes SGA-acetylation in cultivated and wild tomatoes. This finding completes the elucidation of the core Esculeoside A biosynthetic pathway in ripe tomato, allowing reconstitution of Esculeoside A production in heterologous microbial and plant hosts. The involvement of GAME36 in bitter SGA detoxification pathway points to a key role in the evolution of sweet-tasting tomato as well as in the domestication and breeding of modern cultivated tomato fruit.

## Abstract (web summary)

During tomato fruit ripening, bitter and toxic steroidal glycoalkaloids (SGAs) are converted to nonbitter and less toxic forms, but proposed acylating enzyme in pathway remain unknown. Here, authors report BAHD-type acyltransferase that catalyze acylation step in biosynthesis of non-bitter SGAs in tomato.

## Introduction

Steroidal glycoalkaloids (SGAs) are cholesterol-derived specialized metabolites produced by most species of the genus *Solanum*, including major staple food crops such as tomato (*Solanum lycopersicum*), potato (*Solanum tuberosum*) and eggplant (*Solanum melongena*)^1–4^. SGAs protect the plant against a broad range of pathogens and herbivores and have potent pharmacological activity in mammals^2,3,5–7^. However, some SGAs are considered as anti-nutritional to humans due to their high toxicity (e.g., *α*-solanine and *α*-chaconine in potato), bitterness and unpleasant sensations (e.g., *α*-tomatine in tomato)^2,3,5,8^. Despite the presence of these notorious substances that exert a negative impact on nutritional quality and marketability, tomato is the highest-value fruit and vegetable crop (FAO; World Food and Agriculture-statistical yearbook 2021) consumed on a daily basis worldwide. During domestication and breeding, tomato fruit with increased sweetness along with reduced SGA content have been selected for^8,9^.

**Fig. 1:**
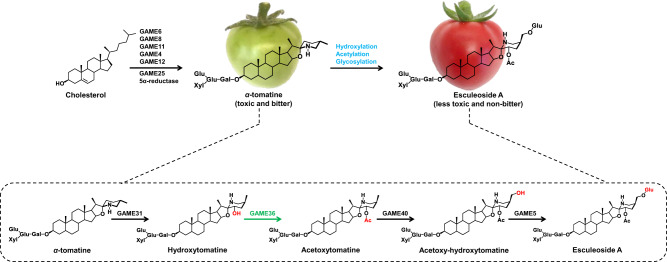
The biosynthetic pathway of non-bitter and less toxic Esculeoside A during tomato fruit development and ripening. The known SGA biosynthetic enzymes (i.e., GAME31, GAME40, and GAME5) in the Esculeoside A pathway are marked in black. The GAME36 enzymatic step discovered in this study is shown in green. Specific activity displayed by these GAME enzymes is shown in red on the SGAs structures. A detailed pathway of tomato SGAs biosynthesis, including the dehydro-SGA branch is presented in Supplementary Fig. 1. GAME: GLYCOALKALOID METABOLISM; Glu: Glucose; Gal: Galactose; Xyl: Xylose; Rha: Rhamnose; Ac: Acetoxy.

*α*-tomatine, a renowned toxic and bitter-tasting compound is the major SGA that accumulates predominantly in leaves and green fruit of tomato^2,7,10^. To prevent the self-toxicity effect, *α*-tomatine is transported and stored in the vacuole by an unknown transporter after its biosynthesis. During the transition from green to red fruit, the entire pool of *α*-tomatine is metabolized to the non-bitter and less toxic Esculeoside A in the cytosolic compartment via a suite of hydroxylation, acetylation, and glycosylation reactions^11–14^ (Fig. 1, and refer Supplementary Fig. 1 for detailed SGA pathway). GORKY, a nitrate/peptide family transporter facilitates this metabolic shift by exporting *α*-tomatine from vacuoles to cytosol during tomato fruit ripening^15^. The relocation of *α*-tomatine back to the cytosol is important where it is available for further enzymatic conversions to form Esculeoside A, the dominant SGA in red, ripe tomato fruit^7,11^. Thus, the major change in SGA composition in the course of fruit ripening is the accumulation of Esculeoside A in the red ripe stage at the expense of *α*-tomatine. Notably, the ripening associated metabolic shift from defense-associated alkaloids (e.g., *α*-tomatine) to Esculeoside A appears to be ethylene-dependent in later stages of fruit development^13^. Moreover, this reduction in bitterness and toxicity in ripe tomato fruit increases palatability for consumption and favors seed dispersal^9,11,13^. Earlier studies^16,17^ suggested that catabolism of the bitter *α*-tomatine during fruit ripening occurs in the following four steps: (i) C-23 hydroxylation of *α*-tomatine to hydroxytomatine, followed by (ii) 23-O-acetylation of hydroxytomatine to form acetoxytomatine; (iii) conversion of acetoxytomatine to acetoxy-hydroxytomatine via C-27 hydroxylation, (iv) which is then converted to Esculeoside A through 27-O-glucosylation (Fig. 1). We recently reported the tomato GLYCOALKALOID METABOLISM31 (GAME31), GAME40 and GAME5 enzymes catalyzing the first C-23 hydroxylation^11^, penultimate C-27 hydroxylation^12,18^ and last 27-O-glucosylation^13^ steps in Esculeoside A biosynthesis, respectively (Fig. 1). However, the biosynthetic enzyme involved in the conversion of hydroxytomatine to acetoxytomatine remains to be identified in any cultivated and wild tomato species.

In this study, we uncovered the missing enzymatic step in the catabolism of the bitter *α*-tomatine in tomato fruit. We identified a BAHD-type acyltransferase enzyme (termed here GLYCOALKALOID METABOLISM 36; GAME36) that catalyzes acylation of various hydroxylated SGAs (e.g., hydroxytomatine) in both cultivated and wild tomato species. Discovery of the GAME36 acyltransferase not merely completes the biosynthetic pathway of non-bitter and less toxic SGAs (e.g., Esculeoside A) in tomato fruit, but also allows the in vivo and in vitro reconstitution of the entire metabolic pathway from *α*-tomatine up to Esculeoside A. Our results expand the understanding of the ripening-associated metabolic shift in SGA composition assuring non-bitter forms of ripe tomato fruits as consumed at present.

## Results

### Acetoxytomatine, a key intermediate in the Esculeoside A pathway accumulates in green fruit of cultivated and wild tomato species

Starting from *α*-tomatine at early fruit development, the biosynthesis of Esculeoside A requires four enzymatic steps (Fig. 1). Having recently uncovered the GAME31, GAME40 and GAME5 enzymes that produce hydroxytomatine, acetoxy-hydroxytomatine and Esculeoside A respectively^11–13^ the only missing step remaining to be discovered in the Esculeoside A pathway was the acetylation of hydroxytomatine to acetoxytomatine (Fig. 1). We envisaged that as for acetoxytomatine, a similar acetylation step and corresponding enzyme might be required to form acetoxy-dehydrotomatine, a dehydro-derivative of acetoxytomatine from hydroxy-dehydrotomatine (Supplementary Fig. 1 for a detailed tomato SGA pathway). A BAHD acyltransferase family protein could possibly catalyze these acetylation steps, as members of this family carry out acylation of diverse substrates in plant specialized metabolic pathways^19,20^. To identify a putative BAHD acyltransferase associated with acetoxytomatine biosynthesis, we searched for BAHD acyltransferase gene candidates co-expressed with known Esculeoside A pathway genes (i.e., *GAME31*, *GAME40,* and *GAME5*) using various tomato transcriptome datasets. Unfortunately, this approach failed to provide any potential BAHD candidates for functional characterization. This could be due to differential regulation of the SGA pathway genes during fruit ripening. Earlier steps of this pathway up to acetoxytomatine (catalyzed by GAME31 and a yet unknown acyltransferase) are predicted to occur in green stage of fruit development in ethylene-independent manner. Further downstream steps towards Esculeoside A performed by GAME40 and GAME5 are ethylene-dependent and importantly, occur in later stages of fruit development (e.g., orange and red ripe stages)^13^. We then tested whether two communal germplasm resources [i.e., Introgression lines (ILs) and Backcross inbred lines (BILs)] derived from crosses between cultivated (*S. lycopersicum cv*. M82) and wild (*S. pennellii* LA0716) tomato species^21,22^ could facilitate BAHD acyltransferase gene discovery via a ‘forward’ genetic screening. We had previously investigated ILs and BILs tomato populations to map the Quantitative Trait Loci (QTLs) associated with SGA biosynthesis in tomato^11^. These approaches proved to be effective as we discovered two key enzymes (GAME31 and GAME5) involved in the Esculeoside A pathway^11,13^. In the context of this study, we next looked for narrow genomic regions associated with acetoxytomatine content in the ILs and BILs. Although we found two QTLs associated with an increase (on chromosome 2) and a decrease (on chromosome 4) in acetoxytomatine levels, the corresponding mapped genomic regions did not contain any putative BAHD acyltransferase gene candidates. As an alternative, we implemented a more comprehensive functional genomics approach and searched for all putative BAHD acyltransferase genes in the cultivated tomato genome. We revealed 100 coding sequences annotated as putative BAHD acyltransferase in the tomato genome (ITAG v2.4) (Supplementary Data 1). However, after excluding the BAHDs with well-characterized functions^20,23^, the list of candidate BAHDs involved in acetoxytomatine biosynthesis could not be narrowed down further based on the sequence. At this point, we reasoned that comparative profiling of acetoxytomatine in different tomato tissues, coupled with transcript expression profiles of BAHDs obtained from the same tissues could aid in prioritizing a reasonable number of candidate genes for further work.

**Fig. 2:**
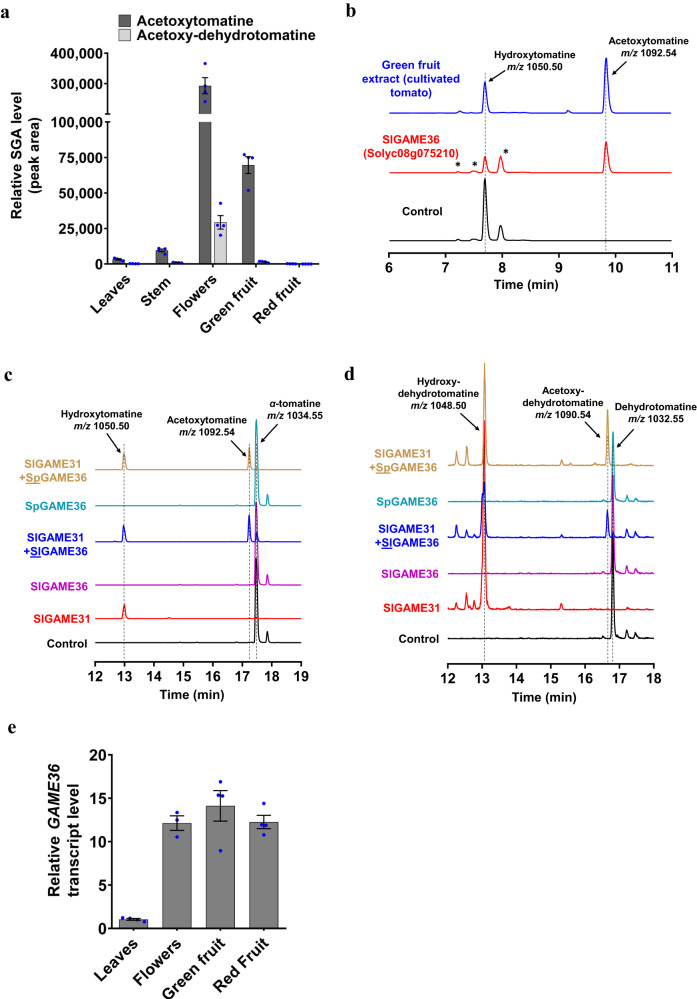
GAME36, a BAHD acyltransferase catalyzes the biosynthesis of acetoxytomatine in cultivated and wild tomato. **a** Profiling of acetoxytomatine and acetoxy-dehydrotomatine in different tissue types of cultivated tomato (*S. lycopersicum cv*. Micro Tom). **b** Acetylation of hydroxytomatine by the recombinant tomato GAME36 (Solyc08g075210) enzyme produced in *E. coli* cells (marked in red). The control reaction (shown in black) was performed with hydroxytomatine using extracts from *E. coli* cells transformed with an empty pET28 vector. The recombinant GAME36 enzyme produces the same acetoxytomatine peak as typically observed in green fruit extracts of tomato (in blue). Aligned ion chromatograms were obtained by LC-MS analysis (short, 15 min. run). The isolated hydroxytomatine compound for in vitro assays contained additional isomers as impurities (shown by asterisks). **c**, **d** The recombinant SlGAME36 [*S. lycopersicum* (*cv*. Micro Tom); shown in blue] and SpGAME36 (*S. pennellii*, in tan, brown) enzymes convert *α*-tomatine (**c**) and dehydrotomatine (**d**) to acetoxytomatine and acetoxy-dehydrotomatine, respectively when assayed together with SlGAME31 (each enzyme produced separately in *E. coli* cells). The control reaction (in black) contained the respective substrate and the protein extracts of empty vector-transformed *E. coli* cells. Individual assays of SlGAME31, SlGAME36 and SpGAME36 recombinant enzymes are shown in red, dark purple and turquoise colors, respectively. Enzyme assays analysis was carried out by LC-MS (40 min. run). Extracted ion chromatograms are shown. **e** *GAME36* gene expression in selected tissues of cultivated tomato (*cv*. Micro Tom) as determined by quantitative Real Time-PCR (qRT-PCR). The values in panel (**a**) and (**e**) indicate the mean of four biological replicates ± standard error mean (*n* = 4) except for flower tissue in panel ‘**e**’ (*n* = 3). Source data are provided as a Source Data file. *m/z*, mass to charge. *E. coli*, *Escherichia coli*.

Using ultra-performance liquid chromatography coupled to quadrupole time-of-flight mass spectrometry (UPLC-qTOF-MS), we examined the distribution of 6 Esculeoside A pathway SGAs including acetoxytomatine, acetoxy-dehydrotomatine, *α*-tomatine, dehydrotomatine, hydroxytomatine, and Esculeoside A in three tissues (i.e., leaves, stem, flowers) and two fruit developmental stages (green and red ripe; skin and flesh together) of the cultivated tomato. We observed higher accumulation of acetoxytomatine and acetoxy-dehydrotomatine in flowers and green fruit compared to leaves, stem and red, ripe fruit of tomato (Fig. 2a). Furthermore, we also noted that all remaining SGAs excluding Esculeoside A showed similar accumulation pattern to the one of acetoxytomatine, but their levels were either very low or undetectable in red, ripe tomato fruit (Supplementary Fig. 2a–c). On the contrary, Esculeoside A was the most abundant SGA found in red, ripe tomato fruit but could not be detected in any other tissue examined (Supplementary Fig. 2d). These results showed that acetoxytomatine accumulates at the green stage of fruit ripening in cultivated tomato. We next followed the distribution of acetoxytomatine and acetoxy-dehydrotomatine during fruit maturation across several wild tomato species. We thus monitored these SGAs in extracts of fruit at four developmental stages (i.e., immature green, mature green, breaker, and ripe; skin and flesh together) across twelve wild tomato accessions. Both acetoxytomatine and acetoxy-dehydrotomatine SGAs were detected among all tested wild tomato accessions, and were more enriched in green fruit (both immature and mature stages) compared to later stages of tomato fruit development (Supplementary Fig. 3).

### Identification of the GAME36 BAHD acyltransferase involved in acetoxytomatine biosynthesis

Directed by the metabolic profiling results, we selected 16 candidate proteins of the BAHD class (excluding the ones characterized earlier) from our in-house generated tomato transcriptome dataset (NCBI SRA: PRJNA307656). This dataset was obtained from tomato (*cv*. Micro Tom) skin and flesh tissues of five fruit developmental stages (immature green, mature green, orange, breaker and red ripe) and various other tissues (young leaf, flower buds, young flower petals and roots). The selected BAHD candidate genes included those that were preferentially expressed in the immature and mature green stages of fruit development versus the red ripe stage [skin and/or flesh with fragments per kilobase of transcript per million mapped reads (FPKM) ≥ 15)]. Additional filtering by transcript expression level in flower buds’ tissue (FPKM ≥ 10) that typically accumulate acetoxytomatine reduced this list to 11 BAHD candidates for further functional characterization (Supplementary Fig. 4). To test their role in acetoxytomatine biosynthesis, we cloned each BAHD candidate from the cultivated tomato and expressed it separately in *E. coli* cells. Purified proteins were then assayed for in vitro acyltransferase activity with hydroxytomatine as a substrate (isolated from transgenic tomato plants overexpressing the *GAME31* gene^11^). Analysis of assay mixtures using a short LC-MS method (15 min. per sample) revealed that one candidate (Solyc08g075210) generated an acetylated compound that is acetoxytomatine (*m/z* 1092.54; C_52_H_85_NO_23_) when incubated with hydroxytomatine (*m/z* 1050.50; C_50_H_83_NO_22_) and acetyl-CoA as acyl donor (Fig. 2b). The acetoxytomatine peak observed here was identical to the one detected in the green fruit extracts of cultivated tomato (Fig. 2b). Thus, in vitro enzyme assays confirmed that candidate Solyc08g075210, named here GLYCOALKALOID METABOLISM36 (GAME36), can catalyze the biosynthesis of acetoxytomatine.

### Recombinant GAME36 enzymes produce a range of acylated SGAs in vitro

Following our finding that the cultivated tomato GAME36 (SlGAME36) generates acetoxytomatine in vitro via acetylation of hydroxytomatine, we sought to assess whether this enzyme can also act on different hydroxylated SGA substrates that are typically found in tomato (e.g., hydroxy-dehydrotomatine, hydroxytomatidine) and eggplant (e.g., hydroxysolamargine) and form the corresponding acetoxy-SGA derivatives. However, since these hydroxylated SGAs are produced at relatively low levels in tomato or eggplant, their isolation for in vitro assays is highly inefficient. To address this issue, we used the previously reported GAME31 enzyme from tomato (SlGAME31) that hydroxylates core SGAs including *α*-tomatine, dehydrotomatine, tomatidine and *α*-solamargine to produce hydroxytomatine, hydroxy-dehydrotomatine, hydroxytomatidine and hydroxysolamargine, respectively^11^. We next performed in vitro coupled enzyme assays using the purified SlGAME31 and SlGAME36 proteins with different SGAs as substrates. Incubation of both recombinant enzymes with *α*-tomatine, acetyl-coA, *α*-ketoglutarate, ascorbate, and Fe^2+^ generated the expected product, acetoxytomatine (Fig. 2c, in blue). Moreover, the combination of SlGAME31 and SlGAME36 enzymes also generated acetoxy-dehydrotomatine (Fig. 2d, in blue), acetoxytomatidine (Supplementary Fig. 5, in blue) and acetoxysolamargine (Supplementary Fig. 6, in blue) from dehydrotomatine, tomatidine and *α*-solamargine, respectively. As previously reported, assays containing SlGAME31 and any of the core SGA substrates (e.g., dehydrotomatine) only produced their corresponding hydroxylated products (e.g., hydroxy-dehydrotomatine) (Fig. 2c, d and Supplementary Figs. 5 and 6; in red). On the other hand, when examined alone, the recombinant SlGAME36 enzyme did not exhibit acyltransferase activity with any of the core SGA substrates (e.g., *α*-tomatine) (Fig. 2c, d and, Supplementary Figs. 5 and 6; in dark purple) suggesting its strict substrate preference towards hydroxylated SGAs. Through a sequence similarity search, we identified a clear ortholog of *GAME36* in wild tomato *S. pennellii* LA0716 (*SpGAME36;* Sopen08g023820), encoding a protein that shares 99% amino acid identity with SlGAME36. The recombinant SpGAME36 protein expressed in *E. coli* tested together with SlGAME31 enzyme and required co-factors also converted *α*-tomatine and dehydrotomatine to acetoxytomatine and acetoxy-dehydrotomatine, respectively (Fig. 2c and d; in tan, brown). Moreover, recombinant SlGAME31 and SpGAME36 enzymes generated acetoxytomatidine and acetoxysolamargine when assayed using tomatidine and *α*-solamargine substrates, respectively (Supplementary Figs. 5 and 6, in tan brown). We also examined the activity of the recombinant SmGAME36 (from cultivated eggplant; 60% amino acid identity with SlGAME36) in coupled assays with SlGAME31 using a similar set of SGA substrates. The recombinant SmGAME36 enzyme did not metabolize hydroxylated SGAs (SlGAME31 assay products) to generate the corresponding acetylated SGA derivatives. The formation of acetylated SGAs by both SlGAME36 (cultivated tomato) and SpGAME36 (wild tomato) proteins suggested that GAME36 has a conserved function in the tomato branch of genus *Solanum*. Altogether, these results showed that GAME36 is capable of catalyzing in vitro acetylation specifically on various hydroxylated SGAs. Acetylated metabolites (enzyme assay products) were putatively identified by comparing their retention times, accurate mass-derived elemental composition, and mass fragmentation pattern with those described for the same in the literature^11,17^. An additional MS/MS analysis was performed to analyze the structures of acetylated compounds (Supplementary Data 3).

To examine the prospect of substrate promiscuity, we next tested the activity of SlGAME36 with various aliphatic (acetyl CoA, malonyl CoA, butyryl CoA, isovalyryl CoA, hexanoyl CoA, octanoyl CoA) and aromatic (coumaroyl CoA and cinnamoyl CoA) acyl donors in coupled assays with SlGAME31 using *α*-tomatine as a substrate. The recombinant SlGAME36 enzyme was able to utilize hydroxytomatine (GAME31 assay product) and all aliphatic acyl donors (except malonyl CoA) tested to generate corresponding acylated SGA metabolites (Supplementary Fig. 7). Notably, SlGAME36 did not show any acyltransferase activity when aromatic CoA thioesters were used as acyl donors (Supplementary Fig. 7). We further set up an additional experiment in which recombinant SlGAME36 enzyme was incubated with hydroxytomatine (acceptor substrate) and mixture of five acyl CoAs (0.2 mM each CoA; acetyl-, butyryl-, isovaleryl-, hexanoyl- and octanoyl-) in a single tube. The acetyl CoA and butyryl CoA were efficiently used as acyl donor with hydroxytomatine, and formed over 93% acylated SGAs together compared to all acylated SGA metabolites generated in the enzyme assay (Supplementary Fig. 8a). In case of hydroxysolamargine as a substrate, we also noted the dominance of acetylated- and butyrylated- SGAs as assay products, suggesting the preference of SlGAME36 enzyme for acetyl CoA and butyryl CoA compared to other acyl donors (Supplementary Fig. 8b). We also examined whether GAME36 could catalyze freely reversible reaction, as reported previously for several other BAHD enzymes^24,25^. Interestingly, GAME36 enzyme did not catalyze reversible reaction when incubated with acetoxytomatine (typical assay product) and CoA as substrates (Supplementary Fig. 9). In addition, we did not observe any spontaneous formation of hydroxytomatine when acetoxytomatine was incubated with CoA, but without GAME36 enzyme (Supplementary Fig. 9).

**Fig. 3:**
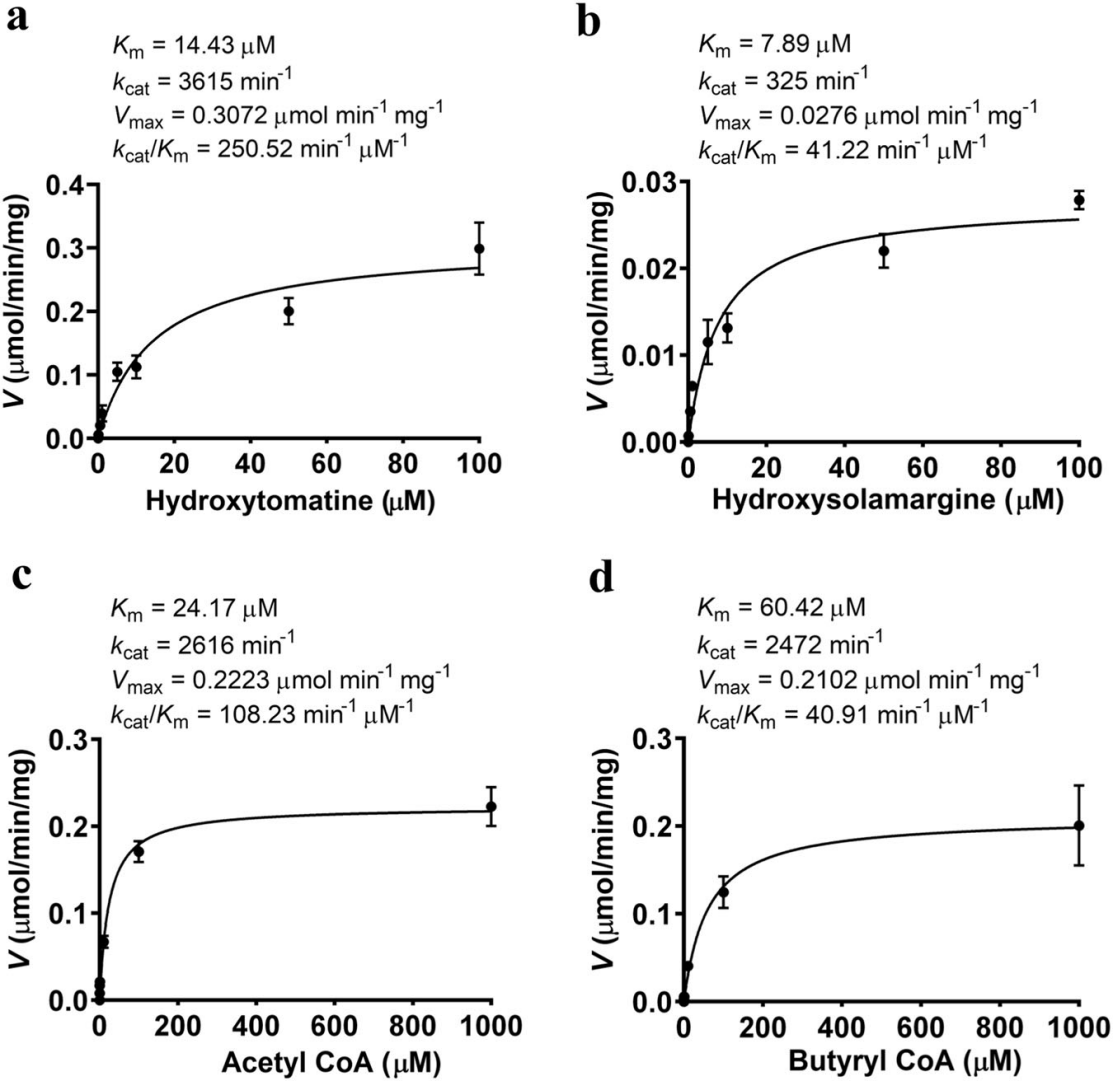
Kinetic characterization of recombinant SlGAME36 enzyme. **a**, **b** Kinetic analysis of SlGAME36 with hydroxytomatine (**a**) and hydroxysolamargine (**b**) as acceptor substrates. **c**, **d** Kinetic analysis of acetyl CoA (**c**) and butyryl CoA (**d**) as acyl donor substrates for SlGAME36. Values represent means ± standard deviation obtained from three independent assays (*n* = 3). Kinetic parameters were calculated using non-linear regression model in the GraphPad Prism 8.0 software. Source data are provided as a Source Data file.

To study the kinetic properties of the SlGAME36 enzyme, we tested its affinity towards acyl donors (acetyl CoA, butyryl CoA) and acyl acceptors (hydroxytomatine and hydroxysolamargine). In vitro steady-state kinetics analysis suggested that SlGAME36 had a higher catalytic efficiency with hydroxytomatine (native substrate from tomato; *k*_cat_/*K*_*m*_ = 250.52 min^−1^ μM^−1^) than hydroxysolamargine (SGA substrate from eggplant; *k*_cat_/*K*_*m*_ = 41.22 min^−1^ μM^−1^) (Fig. 3a, b). In case of acyl donors, enzyme kinetic assays showed preferred specificity toward acetyl CoA over butyryl CoA (*k*_cat_/*K*_*m*_ = 108.23 min^−1^ μM^−1^ for acetyl CoA compared with *k*_cat_/*K*_*m*_ = 40.91 min^−1^ μM^−1^ for butyryl CoA) (Fig. 3c, d). Overall, our results demonstrated the promiscuity of the GAME36 enzyme in vitro by acylating hydroxy-SGAs using various aliphatic acyl CoA donors. Hydroxytomatine and hydroxysolamargine substrates used in the biochemical characterization of GAME36 were isolated from in vitro enzyme assays using GAME31 with either *α*-tomatine or *α*-solamargine, respectively (See Methods section for more details).

### *GAME36* expression is associated with acetoxytomatine accumulation in cultivated and wild tomato species

Acetoxytomatine is one of the few SGAs commonly detected in almost every cultivated and wild tomato species. Yet, Iijima et al. (2013)^17^ reported the absence of acetoxytomatine and further downstream SGAs, acetoxy-hydroxytomatine and Esculeoside A in ripe fruit of the wild tomato *S. neorickii* (accession LA2133), but detected high levels of neorickiioside B (i.e., hydroxytomatine) in the same tissue. This suggested that biosynthesis of Esculeoside A from *α*-tomatine is inhibited at the acetylation step of hydroxytomatine in *S. neorickii* (LA2133). Here, we examined SGAs content in leaves and ripe fruit of *S. neorickii* (LA2133) and the cultivated tomato. Notably, very high levels of acetoxytomatine were observed in the leaves of *S. neorickii* compared to leaves of the cultivated tomato (Supplementary Fig. 10a). Moreover, we detected a set of SGAs associated with fruit ripening (i.e., hydroxytomatine, acetoxytomatine, acetoxy-hydroxytomatine and Esculeoside A) in ripe fruit of *S. neorickii* (Supplementary Fig. 10b, c). The results indicate that Esculeoside A biosynthesis indeed takes place in *S. neorickii* (LA2133) fruit that likely contains the corresponding enzyme activities required for its biosynthesis. We next compared *GAME36* expression levels in leaves of cultivated tomato and *S. neorickii*. *GAME36* showed very weak expression in leaf tissue of cultivated tomato (Supplementary Fig. 10d) correlating with trace levels of acetoxytomatine in the leaves of this genotype (Supplementary Fig. 10a). *S. neorickii* displayed very high *GAME36* expression as compared to the cultivated tomato leaves (Supplementary Fig. 10d) which explains the presence of very high acetoxytomatine levels in this accession (Supplementary Fig. 10a).

We next measured *GAME36* expression in different tissues of cultivated tomato and found that it was highly expressed in green fruit and flowers as compared to leaves (Fig. 2e), significantly correlating with acetoxytomatine content in the same tissues (Fig. 2a). Interestingly, *GAME36* was also highly expressed in red, ripe fruit (Fig. 2e), that indeed accumulated very low levels of acetoxytomatine (Fig. 2a). Next, we examined the expression of *GAME36* in four fruit developmental stages of twelve wild tomato accessions (RNA-seq expression data)^12^. *GAME36* displayed predominant expression in immature and mature green stage (skin and flesh together) of fruit development (Supplementary Fig. 11). Overall, *GAME36* expression profile correlated well with acetoxy-SGAs (i.e., acetoxytomatine and acetoxy-dehydrotomatine) levels (Supplementary Fig. 3).

### Knocking out of *GAME36* alters SGA metabolism in tomato

**Fig. 4:**
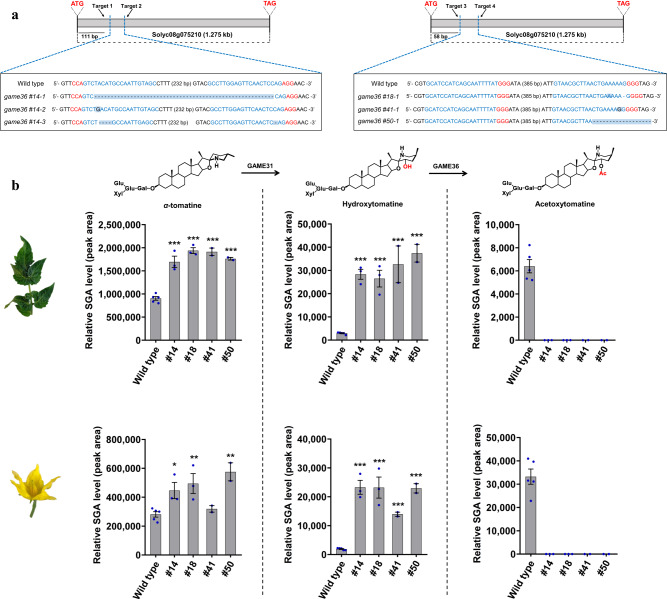
CRISPR-cas9 mediated knockout of GAME36 results in loss of acetoxytomatine in leaves and flowers of tomato. **a** Schematic representation of *GAME36* with location of guide RNA sequences, CRISPR-Cas9 cleavage sites and *game36* mutant sequences. Four independent, homozygous *game36* mutant lines (#14, #18, #41 and #50) were generated in the tomato (*cv*. Micro Tom) background. Protospacer adjacent motifs (PAMs) and inserted bases are shown in red and blue respectively and deleted bases are replaced by a dash. Selected *game36* mutant sequences are presented here. Refer to Supplementary Fig. 12 for additional *game36* mutant sequences obtained in this study. **b** Levels of *α*-tomatine, hydroxytomatine, and acetoxytomatine SGAs in leaves and flowers of *game36* mutant lines as compared to wild type ones. Acetoxytomatine was not detected in the *game36* mutant lines. The values indicate means of biological replicates ± standard error mean (*n* = 4 for wild type, *n* = 3 for #14 and #18 lines, and *n* = 2 for #41 and #50 lines). Asterisks indicate significant changes compared to wild type samples as calculated by a two-tailed Student’s t-test (*P-value < 0.05; **P-value < 0.01; ***P-value < 0.001). LC-MS was used for targeted SGAs analysis. Source data (for panel **b**) are provided as a Source Data file.

The in vivo function of GAME36 was examined by generating loss-of-function mutants in the cultivated tomato using CRISPR-Cas9 genome editing (Fig. 4a and Supplementary Fig. 12). Homozygous *game36* mutant lines were analyzed for the altered SGA profiles. We could not detect acetoxytomatine and acetoxy-dehydrotomatine in leaves (4–6 weeks old) and flowers of the *game36* mutant lines (Fig. 4b and Supplementary Fig. 13). These SGAs were present in both flowers (high levels) and leaves (low levels) of wild type (non-transformed) tomato plants (refer Fig. 2a). Conversely, we observed significant accumulation of acetoxy-SGAs precursors (i.e., hydroxytomatine and hydroxy-dehydrotomatine) and additional upstream SGAs (i.e., *α*-tomatine and dehydrotomatine) in the flowers and leaves tissues compared to wild type ones (Fig. 4b and Supplementary Fig. 13). Moreover, both leaves and flowers of the *game36* mutant lines showed a major increase in hydroxytomatine isomers that are typically present in minor levels in these tissues (Supplementary Fig. 14).

**Fig. 5:**
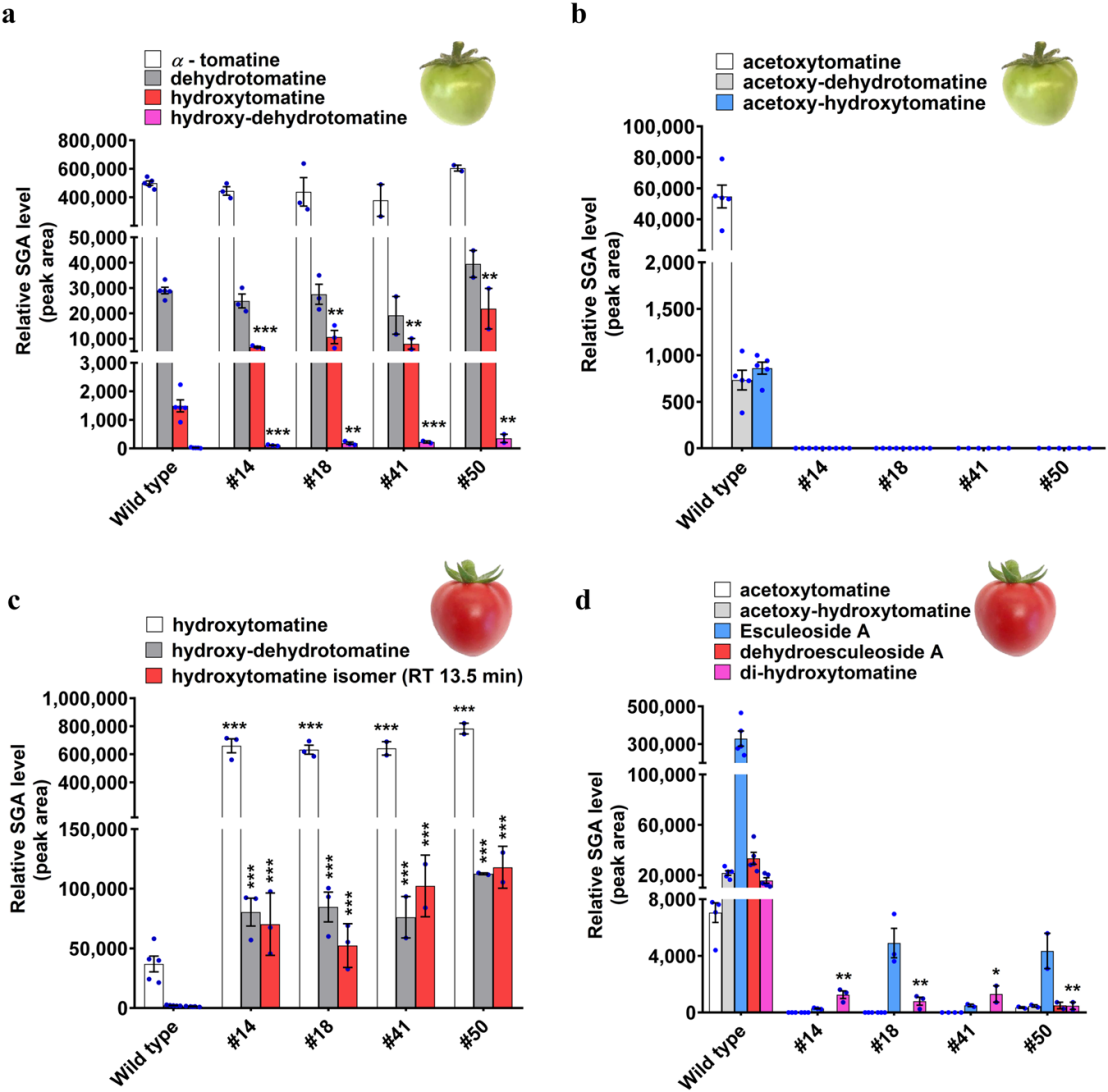
Fruit of game36 mutant tomato lines display significantly altered SGA metabolism. **a**–**d** Levels of SGAs produced upstream of the GAME36 reaction step (**a**, **c**), and downstream of the GAME36 reaction step (**b**, **d**) in green (**a**, **b**) and red (**c**, **d**) fruit of *game36* mutant tomato lines as compared to wild type fruit. Refer Supplementary Fig. 1 for SGA pathway intermediates. Knockout of *GAME36* resulted in significant accumulation of precursor SGAs (i.e., hydroxytomatine, hydroxy-dehydrotomatine, hydroxytomatine isomers) with almost complete loss of downstream SGAs (acetoxytomatine, acetoxy-hydroxytomatine and Esculeoside A) in tomato fruit tissues. Notably, hydroxylated SGAs are the most abundant SGAs in red fruit of *game36* mutant lines compared to the wild type red fruit. The values indicate mean of biological replicates ± standard error mean (*n* = 4 for wild type, *n* = 3 for #14 and #18 lines and *n* = 2 for #41 and #50 lines). Asterisks indicate significant changes compared to wild type samples as calculated by a two-tailed Student’s *t*-test (*P-value < 0.05; **P-value < 0.01; ***P-value < 0.001). LC-MS was used for targeted SGAs profiling. Source data (panel **a**, **c**, **d**) are provided as a Source Data file.

During tomato fruit ripening (from green up to the red ripe stage), *α*-tomatine and dehydrotomatine are converted to Esculeoside A and dehydro-esculeoside A, respectively. To investigate the impact of GAME36 activity on ripening-associated SGA metabolism, we compared the SGA profile of wild type and *game36* tomato fruits. Analysis of green fruit from *game36* mutant lines showed dramatic accumulation of hydroxytomatine and hydroxy-dehydrotomatine compared with wild-type green fruit (Fig. 5a). Furthermore, no major change in *α*-tomatine and dehydrotomatine levels was observed in green fruit of the *game36* mutant lines. In contrast, we did not detect acetoxy-dehydrotomatine, acetoxytomatine, and acetoxy-hydroxytomatine, the latter SGA derived from acetoxytomatine through additional hydroxylation, in *game36* green fruit (Fig. 5b, refer Supplementary Fig. 1 for SGA pathway). Thus, reduced *GAME36* activity in the green fruit resulted in the complete loss of acetoxy-SGAs with concomitant buildup of its precursor SGAs. In red, ripe fruit of the *game36* mutant lines, we detected massive accumulation of hydroxytomatine, hydroxytomatine isomer, and hydroxy-dehydrotomatine SGAs in comparison to wild-type fruit at the same stage (Fig. 5c). In fact, these precursor SGAs were the predominant SGAs in red fruit of the *game36* mutant tomato plants. In parallel, SGAs produced downstream of hydroxytomatine (i.e., acetoxytomatine and acetoxy-hydroxytomatine) towards Esculeoside A were not detected in ripe *game36* fruit (apart from mutant line #50 that showed negligible levels). Yet, we observed minimal levels of Esculeoside A in red ripe fruit of the *game36* mutant lines (Fig. 5d). Furthermore, we detected a substantial decrease in di-hydroxytomatine SGA (a likely derivative of hydroxytomatine) in comparison to wild type ripe fruit (Fig. 5d). Altogether, our in vivo and in vitro results confirmed that tomato GAME36 catalyzes the acetylation of hydroxytomatine to acetoxytomatine in the Esculeoside A biosynthetic pathway.

### In vitro and *in planta* reconstruction of Esculeoside A biosynthesis

**Fig. 6:**
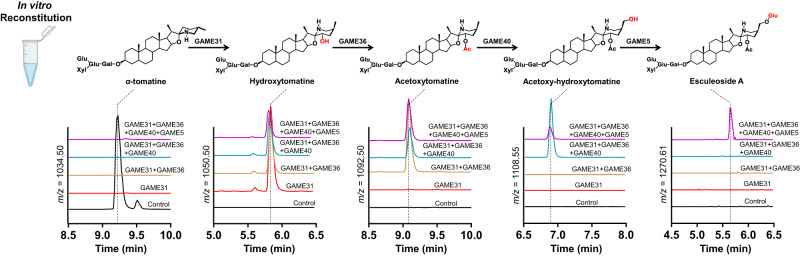
In vitro reconstruction of Esculeoside A biosynthesis. Consecutive activities of the recombinant SlGAME31, SlGAME36, SlGAME40, and SlGAME5 proteins (produced separately in *E. coli* cells) convert *α*-tomatine to Esculeoside A in a single tube reaction. Aligned ion chromatograms from LC-MS (short 15 min. run) are presented. The control reaction (shown in black) was performed with *α*-tomatine as substrate using extracts from *E. coli* cells transformed with an empty pET28 vector. For in vitro production of each intermediate in the Esculeoside A pathway, sequential assays with the corresponding GAME enzymes were performed. Mass to charge (*m/z*) is shown for *α*-tomatine and the assay products (i.e., Esculeoside A pathway intermediates).

The uncovered GAME36 activity together with the previously characterized GAME enzymes (GAME31, GAME40 and GAME5)^11–13^ completes the SGA pathway branch converting *α*-tomatine to Esculeoside A. These GAME proteins belong to three different enzyme families namely, 2-Oxoglutarate-Dependent Dioxygenases [2-ODDs; i.e., GAME31 and GAME40), BAHD acyltransferases (GAME36) and Uridine diphosphate (UDP) glycosyltransferases (UGTs: GAME5). We next asked whether the four recombinant GAME enzymes could metabolize *α*-tomatine to produce Esculeoside A. We reconstructed the Esculeoside A biosynthetic pathway by adding the four purified enzymes expressed in *E. Coli* (i.e., GAME31, GAME36, GAME40, and GAME5), *α*-tomatine and the appropriate co-factors (*α*-ketoglutarate, ascorbate, Fe^2+^, acetyl-CoA and UDP-glucose) in a single tube. We noted that this reaction produced not only the final product, Esculeoside A but also all pathway intermediates (hydroxytomatine, acetoxytomatine, and acetoxy-hydroxytomatine) (Fig. 6) that accumulate in vivo in numerous cultivated and wild tomato species. As the order of reactions of the Esculeoside A pathway is confirmed here as proposed previously^10,16^, stepwise reconstitution experiments were performed by sequentially adding the respective GAME enzymes, *α*-tomatine, and relevant co-factors in a single tube. Sequential assays produced explicitly the expected pathway intermediates (e.g., hydroxytomatine) associated with Esculeoside A pathway (Fig. 6).

Following the successful in vitro reconstruction experiments, we attempted to reconstruct Esculeoside A biosynthesis *in planta* by infiltrating *N. benthamiana* leaves with *Agrobacterium* harboring the respective *GAME* genes and *α*-tomatine as a substrate (*α*-tomatine infiltrated three days post infiltration). Transient co-expression of tomato *GAME31*, *GAME36*, *GAME40* and *GAME5* in *N. benthamiana* leaves supplemented with *α*-tomatine generated Esculeoside A (Supplementary Fig. 15). As observed in our in vitro experiments, co-infiltration of the respective tomato GAME enzymes in a sequential manner (according to the pathway reaction order, see Fig. 1) and subsequent supplementation with *α*-tomatine resulted in formation of the corresponding Esculeoside A pathway intermediates (Supplementary Fig. 15). Furthermore, we designed additional co-infiltration combinations of *GAME* genes in the same transient expression experiment to check whether respective GAME enzymes can act promiscuously on pathway intermediates, and form new SGA derivatives. We did not observe the accumulation of any other SGAs apart from the expected ones in such reconstitution experiments, therefore, eliminating the possibility of cross-reactivity of GAME enzymes on Esculeoside A pathway intermediates. These results validate the sequential function of GAME31, GAME36, GAME40, and GAME5 enzymes in the production of tomato fruit ripening-associated SGAs.

### Phylogenetic analysis suggests functional diversification of GAME36 proteins in tomato

**Fig. 7:**
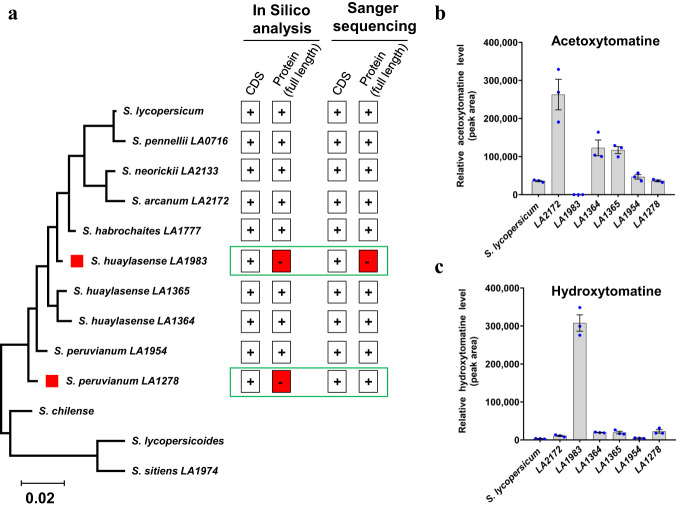
Phylogenetic context of tomato GAME36 proteins. **a** GAME36 genotype variants obtained from cultivated and wild tomato species. ‘+’ (plus) sign indicates presence of genotype (e.g. coding sequence and/or full-length protein) in a particular tomato species. Red square with ‘-’ (negative) sign marks the absence of full-length GAME36 proteins in the *S. huaylasense* LA1983 and *S. peruvianum* LA1278 accessions. The predicted GAME36 genotypes were further confirmed and compared by Sanger sequencing of cloned GAME36 sequences from respective wild tomato species. **b**, **c** LC-MS based profiling of acetoxytomatine (**b**) and hydroxytomatine (**c**) in leaves of cultivated and wild tomato accessions. The values indicate means of biological replicates ± standard error mean (*n* = 3), obtained from three independently grown tomato accession plants. Source data (panel **b** and **c**) are provided as a Source Data file.

We explored the genetic variation in *GAME36* by extracting the coding sequences from cultivated tomato and related wild accessions (from Sol Genomics Network server), representative of tomato clade in the genus *Solanum*^26^. We noted the presence of the *GAME36* gene in all the selected accessions (Fig. 7a). However, in two wild accessions, *S. huaylasense* LA1983 and *S. peruvianum* LA1278, we noticed two nonsense SNPs in *GAME36* (one in each accession) that introduced premature stop codons, therefore likely producing truncated, non-functional GAME36 variant proteins (Supplementary Fig. 16a). We next amplified and sequenced the *GAME36* coding region from several wild tomato accessions and examined the completeness of sequences. Notably, we could only confirm the nonsense mutation in *S. huaylasense* LA1983, but not in *S. peruvianum* LA1278 (Supplementary Fig. 16b & 16c) as predicted. Thus, the nonsense SNP originally identified for *GAME36* in *S. peruvianum* LA1278 was a sequencing discrepancy, which remained unverified by further analysis in the genome of *S. peruvianum* LA1278 accession. The other wild accessions including *S. peruvianum* LA1278 displayed full-length *GAME36* sequences, encoding further full-length GAME36 proteins (Fig. 7a). We further analyzed acetoxytomatine and hydroxytomatine content in leaves of these selected wild tomato accessions. Cultivated tomato (*S. lycopersicum cv*. Moneymaker) accumulate these SGAs in its leaves and possess functional GAME36 protein, thus was included in genomic and metabolite analysis as positive control. While acetoxytomatine was detected in almost all wild tomato species, we could not find it in *S. huaylasense* LA1983 accession (Fig. 7b). Furthermore, very high levels of hydroxytomatine (precursor of acetoxytomatine) were observed in *S. huaylasense* LA1983 accession compared to other wild species (Fig. 7c). These results suggest that the non-functional GAME36 enzyme in *S. huaylasense* LA1983 prevents the formation of acetoxytomatine in this accession.

**Fig. 8:**
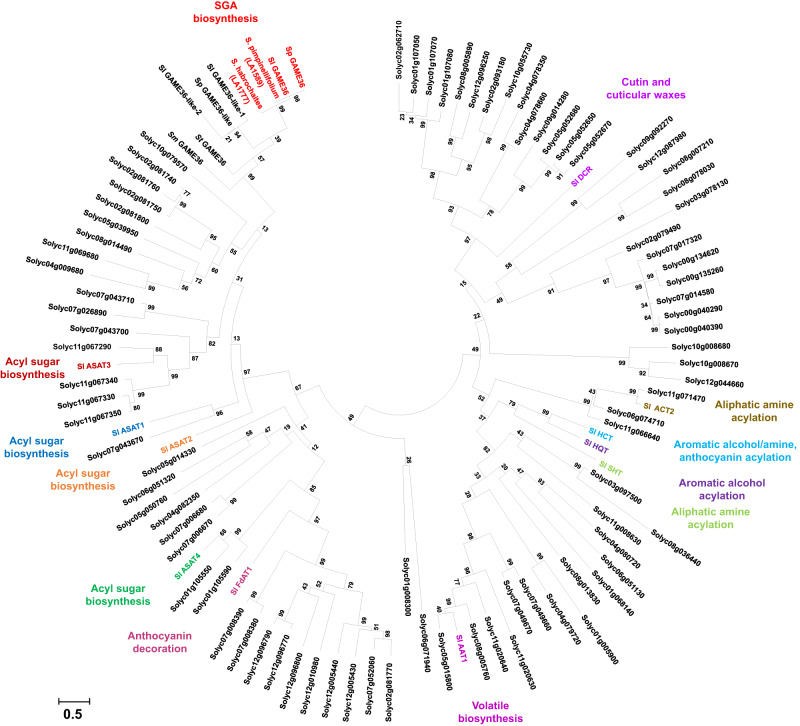
Evolutionarily distinct BAHD acyltransferase activity completes the detoxification pathway of bitter steroidal glycoalkaloids (SGAs) in tomato. GAME36 proteins performing acetylation reactions in SGA biosynthesis form a distinct monophyletic clade (marked in red) in phylogenetic analysis comprising all tomato BAHDs. Functionally characterized BAHD proteins appearing in separate clades according to their functional diversity in tomato are marked in different colors in phylogenetic tree. Amino acid sequences used in the phylogenetic analysis are provided in Supplementary Data 2. The evolutionary history was inferred using the maximum-likelihood method in MEGAX using 1000 bootstrap replications. Sequences from the following species were represented: cultivated tomato [*S. lycopersicum*, (Sl)], cultivated potato [*S. tuberosum*, (St)], wild tomato [*S. pennellii*, (Sp)] and cultivated eggplant [*S. melongena*, (Sm)]. ASAT, acylsugar acyltransferase; HCT; hydroxycinnamoyl-CoA shikimate hydroxycinnamoyltransferase; DCR; defective in cuticular ridges; HQT, hydroxycinnamoyl CoA quinate hydroxycinnamoyltransferase; SHT, spermidine N-hydroxycinnamoyltransferases; AAT1, alcohol acetyltransferase 1; ACT2, aliphatic amine acyltransferase 2; FdAT1, Flavonoid-3-O-rutinoside-4”‘-phenylacyltransferase 1 (FdAT1); GAME36, glycoalkaloid metabolism36.

Phylogenetic analysis of all tomato BAHD proteins including GAME36, and its homologous sequences from other *Solanum* species showed that GAME36 from cultivated and wild tomato species are clearly separated from the rest of the BAHD proteins (Fig. 8, in red). These proteins formed a distinct clade, supporting the unique divergent function of this BAHD in SGA biosynthesis. Interestingly, we detected three clustered BAHD acyltransferase genes in the cultivated tomato chromosome 8 spanning ~13 Kbp region that includes a single *GAME36* and two truncated *GAME36-like* homologs (Supplementary Fig. 17). Both of these truncated proteins lack either HKIDAG (in case of *GAME36-like-1*) or DFGWG (in case of *GAME36-like-2*) motifs in their sequences. In wild tomato *S. pennellii*, we noticed the presence of GAME36 and full-length GAME36-like homolog (67% homology at amino acid level) on chromosome 8 within ~13 Kbp genomic region (Supplementary Fig. 17). Notably, recombinant *S. pennellii* GAME36-like protein did not exhibit acyltransferase activity when tested with hydroxytomatine or hydroxysolamargine substrates. The GAME36-like proteins from cultivated and wild tomato are located next to GAME36 clade in the phylogeny.

Despite 60 to 75% homology (at amino acid level) with GAME36 proteins, coupled recombinant assays of the GAME36 enzymes either from cultivated potato (present on chromosome 11) or eggplant (chromosome 8) together with GAME31 did not show any acyltransferase activity with various hydroxylated SGAs substrates (products of the GAME31 reaction). Moreover, there is no GAME36-like homolog present in the cultivated potato and eggplant genome (Supplementary Fig. 17). The presence or absence of *GAME36-like* genes is evident both in homology searches as well as in comparisons of syntenic regions between cultivated tomato, cultivated eggplant, and wild *S. pennellii* (Supplementary Fig. 18). In tomato, apparently very few (11 out of the 100 BAHDs) have been experimentally characterized. ASAT1 to ASAT4 proteins involved in acyl sugar biosynthesis^23,24,27^ form separate clades based on their role in the acylation of different sugar and acyl-CoA substrates (Fig. 8). DCR, AAT1, and FdAT1 proteins involved in cutin and wax metabolism^28^, volatile biosynthesis^29^ and anthocyanin decoration^30^ respectively form distinct sub-clades. Four BAHD proteins (ACT, HCT, SHT, and HQT) proteins participating in the acylation of primary alcohol/amines and anthocyanins^20,31^ are also clearly separated from the rest of the BAHD proteins (Fig. 8). Thus, the versatile roles displayed by tomato BAHD proteins including GAME36 are reflected in the phylogenetic analysis, which suggests that the evolution of BAHD family members drive the expansion of metabolic diversification in tomato.

## Discussion

The drop in bitter and toxic *α*-tomatine levels during tomato fruit maturation has been known for decades^32–34^. Yet, only two reports by Mintz-Oron et al. (2008)^16^ and Iijima et al. (2013)^17^ proposed a putative metabolic pathway that converts *α*-tomatine to Esculeoside A. Prediction of the Esculeoside A pathway was based on metabolomics experiments obtained from fruit tissues at several developmental stages up to ripening. Both reports suggested that as fruit matures the entire pool of *α*-tomatine is converted through four core reactions to its hydroxylated, acylated, and glycosylated derivatives (e.g., Esculeoside A). The enzymes and order of reactions in the *α*-tomatine catabolic pathway were unknown at that time. Here we report the discovery of GAME36, an acyltransferase of the BAHD protein family that completes the identification of genes and enzymes predicted in the Mintz-Oron et al. (2008)^16^ and Iijima et al. (2013)^17^ pathway. Consequently, this allowed us to determine the order of reactions in the pathway to Esculeoside A. The main tomato SGA *α*-tomatine starts accumulating at fruit set reaching its maximum levels in mature green fruit. At this stage of development, the GAME31 dioxygenase hydroxylates *α*-tomatine at the C-23 position forming hydroxytomatine^11^. GAME36, identified in this study next acetylates hydroxytomatine at the same position forming acetoxytomatine, which is then subjected for a second hydroxylation in the pathway (at position C-27) by GAME40, generating acetoxy-hydroxytomatine. The last step of the pathway is carried out by GAME5, glycosylating acetoxy-hydroxytomatine (at the 27-O position) to form Esculeoside A which accumulates to high levels in red, ripe fruit. Interestingly, unlike other reported BAHD enzymes (e.g., ASATs involved in acylsugar biosynthesis in tomato), GAME36 does not catalyze freely reversible reactions on pathway intermediates, thus ensuring these intermediates are available for further downstream GAME enzymes in the Esculeoside A pathway. Furthermore, in vivo characterization of *GAME36* by knockout experiments revealed the possibility of an alternative, yet minor biosynthetic route to Esculeoside A in tomato fruit. Apart from the typical three intermediates (that is hydroxytomatine, acetoxytomatine and acetoxy-hydroxytomatine), previous studies proposed di-hydroxytomatine as an additional intermediate in the Esculeoside A pathway^10^. Di-hydroxytomatine accumulates mainly in the late stages of tomato fruit ripening such as orange and red ripe fruits. This SGA was predicted to be synthesized from hydroxytomatine via additional hydroxylation, and further hypothesized to be converted to acetoxy-hydroxytomatine via acetylation reaction (Supplementary Fig. 19 for alternative Esculeoside A pathway). In this study, despite the complete absence of acetoxytomatine in green and red fruit of *game36* mutant lines, we observed very low levels of Esculeoside A and its precursor acetoxy-hydroxytomatine (e.g., line#50 in Fig. 5D) in the mutant red fruits. This indicates that both these SGAs are formed in red fruit and specifically; there is an alternative route for acetoxy-hydroxytomatine biosynthesis likely from di-hydroxytomatine as predicted. This claim is further supported by reduced levels of di-hydroxytomatine in *game36* mutant red fruits compared to wild-type ones, which could be due to channeling towards acetoxy-hydroxytomatine and Esculeoside A biosynthesis. We believe that this minor pathway for Esculeoside A biosynthesis involving di-hydroxytomatine operates exclusively in late stages of fruit ripening like red ripe, and involves yet unknown ripening stage specific hydroxylase and acyltransferase enzymes to form di-hydroxytomatine and its downstream derivative, acetoxy-hydroxytomatine respectively (Supplementary Fig. 19).

The ripening-associated changes in SGA metabolism (conversion of *α*-tomatine to Esculeoside A) are controlled by the ripening-inducing phytohormone ethylene, and exogenous ethylene treatments of tomato fruit accelerated this chemical shift (higher Esculeoside A and lower *α*-tomatine)^35^. On the contrary, ripening-impaired mutants such as *ripening-inhibitor* (*rin*), *non-ripening* (*nor*) and *Never-ripe* (*Nr*) showed reduced levels of Esculeoside A, but accumulated its pathway intermediates (e.g., hydroxytomatine, acetoxytomatine) compared to wild type fruit^35^. These results in the tomato mutant lines suggest that the late steps in Esculeoside A pathway depend on ethylene during ripening. In fact, GAME40 (also known as E8) catalyzing the penultimate step in Esculeoside A pathway is a known ethylene-responsive gene during fruit ripening and regulated by ethylene and several other transcription factors involved in fruit ripening^18,36^. Moreover, the expression of GAME5 transcript (associated with last step in Esculeoside A pathway) was severely affected in ripening mutants compared to wild type fruit, where it is highly upregulated at the breaker stage (Supplementary Fig. 20). The earlier steps in the Esculeoside A pathway carried out by GAME31 and GAME36 appear to be ethylene-independent as expression of these genes was not affected in ripening impaired tomato mutants. This ripening associated chemical shift in SGAs metabolism was likely selected during tomato fruit domestication and breeding to prevent the accumulation of *α*-tomatine in ripe fruit that results in bitter flavor. Evolutionary wise, reducing the toxicity of fruit could facilitate feeding by frugivores and consequently promote seed dispersal.

Besides their role in defense in plants and anti-nutritional effects to humans, several SGAs from tomato (*α*-tomatine), potato (*α*-chaconine and *α*-solanine) and eggplant (*α*-solasonine and *α*-solamargine) are known for their anti-carcinogenic potential^37,38^. Several studies reported multiple therapeutic applications of Esculeoside A in human health^39–42^. For example, Fujiwara et al.^39,40^ isolated Esculeoside A from ripe tomato fruits, and showed its potent cytotoxic activity against MCF7 (human breast cancer cell) and B16F2F (mouse melanoma cell) cells. The GI_50_ values of Esculeoside A were reported to be 13.3 and 7.9 µM against MCF7 and B16F2F, respectively. Moreover, Yang et al.^41^ proposed a possible application of Esculeoside A as a functional supplement for diabetes treatment using mice model. Another study by Zhou et al.^42^ showed the role of Esculeoside A in ameliorating atopic dermatitis in mice (IC_50_ 9 µM) via blocking hyaluronidase activity in vitro and in vivo.

It is now established that *α*-tomatine provides a bitter taste as exemplified nicely by the early work of Charles Rick and co-workers as well as ours on the *gorky* mutant^15^. During tomato fruit maturation and ripening, the bitter-tasting *α*-tomatine is metabolized to the non-bitter Esculeoside A. Some commercial cultivars contain relatively substantial *α*-tomatine levels in ripe fruit. This might be a source of bitterness in commercial tomato varieties. Thus, the understanding Esculeoside A biosynthesis could facilitate the development of genetic markers or genome modifications that will maximize the conversion of *α*-tomatine to Esculeoside A and consequently increase fruit sweetness. Moreover, as SGAs and particularly Esculeoside A, have been reported to possess health-promoting properties, results of our study could be applicable for the production of high-value products through metabolic engineering approaches.

## Methods

### Plant material

Cultivated tomato (*Solanum lycopersicum cv*. Micro Tom and *cv*. M82), wild tomato accessions [*S. habrochaites* (LA1777 and LA0407), *S. chmielewskii* (LA1318, 732 and LA1028), *S. peruvianum* (PI126431 and PI126926), *S. pennellii* (LA0716), *S. pimpinellifolium* (LA1589 and LA1586), *S. cheesmaniae* (LA1412 and LA1306) and *S. neorickii* (LA2133)] and cultivated eggplant (*S. melongena*) plants were grown in a climate-controlled greenhouse at 24 °C during the day and 18 °C during night, with natural light. *Nicotiana benthamiana* plants were grown in a growth room maintained at 23 ± 2 °C with 16-h day/8-h night light regime.

### Analytical standards

Analytical standards including tomatidine (Sigma-Aldrich, contains dehydrotomatidine as impurity), *α*-tomatine (Carbosynth USA, contains dehydrotomatine as impurity), *α*-solamargine (ChemFaces) were dissolved in methanol at a concentration of 1 mg/ml. Hydroxytomatine used as substrate in recombinant assays during screening of BAHD candidates (Fig. 2b) was isolated and purified from leaves of *GAME31*-overexpressing transgenic tomato plants as described earlier^11^.

### Plant extracts preparation and LC-MS based SGA analysis

Preparation of extracts and SGAs profiling in various tissues (leaves, stem, flowers, green and red fruit) of cultivated and wild tomato varieties were performed with the same methods described earlier^7,10,43^. Briefly, 100 mg of frozen powder fruit tissue was extracted with 80% methanol and 0.1% formic acid, briefly vortexed and then sonicated for 20 min at room temperature. Finally, the extracts were centrifuged for 15 min at 20,000 × *g* and filtered through 0.22 μm filters. Samples were analyzed using a high-resolution UPLC/qTOF system comprised of a UPLC (Waters Acquity) connected to a qTOF detector (tandem quadrupole/time-of-flight mass spectrometer, Waters) with the standard (long gradient, 40 min, positive mode) run conditions as follows: from 100 to 72% phase A over 22 min, from 72 to 0% phase A over 14 min, then held at 100% phase B for 2 min; and then returned to the initial conditions (100% phase A) within 0.5 min and conditioning at 100% phase A for 1.5 min. The mobile phase consisted of 0.1% formic acid in acetonitrile:water (5:95, v/v; phase A) and 0.1% formic acid in acetonitrile (phase B). SGAs were identified by comparing the retention time and mass spectra of authentic standards analyzed on the same instrument (see above). When the corresponding standards were not available, SGA metabolites were putatively identified by comparing their retention times, elemental composition, and mass fragmentation pattern with those described in the literature^10,17,44^. Relative quantification of the SGA metabolites was carried out using the TargetLynx (Waters) program.

### Generation of CRISPR/Cas9 edited *game36* mutant tomato lines

To generate the CRISPR/Cas9 engineered *SlGAME36* (cultivated tomato *cv*. Micro Tom) mutant plants, four guide RNAs (gRNAs) were selected using the CRISPR-P 2.0 web tool (http://cbi.hzau.edu.cn/CRISPR2/)^45^. gRNA expression cassettes were prepared and assembled into CRISPR/Cas9 vector as described previously^46^. The final CRISPR/Cas9-gRNA construct was introduced into *A. tumefaciens* (GV3101) electrocompetent cells, and further transformed into tomato (*cv*. Micro Tom) as described previously^11^. Briefly, cotyledon explants were excised from the 7-day-old in vitro grown tomato seedling and cut near the petiole and tip, placed on a plate containing appropriate co-cultivation media, and preincubated for 24 h under dark conditions. Co-cultivation of excised explants with *Agrobacterium* (OD_600_ = 0.4) was carried out for 48 h under dark conditions. After co-cultivation period, explants were transferred to shoot induction medium containing zeatin (2 μg ml^−1^), indole-3-acetic acid (IAA; 0.2 μg ml^−1^), kanamycin (50 μg ml^−1^), and ticarcillin (250 μg ml^−1^) for 3–4 weeks, and then transferred on shoot elongation medium containing zeatin (1 μg ml^−1^), zeatin riboside (1 μg ml^−1^), IAA (0.2 μg ml^−1^), kanamycin (50 μg ml^−1^), and ticarcillin (100 μg ml^−1^). Subsequently, well-developed shoots were excised and transferred to rooting medium containing indole-3-butyric acid (IBA; 2 μg ml^−1^), kanamycin (50 μg ml^−1^), and ticarcillin (100 μg ml^−1^). After 3 to 4 weeks, plantlets with roots were transferred to greenhouse and maintained for further analysis.

### CRISPR-Cas9 mutant identification and genotyping

CRISPR/Cas9 induced mutations were genotyped in T_1_ plants using DNA sequencing. Briefly, the genomic DNA was prepared from fresh frozen T_1_ leaves (80 to 100 mg) using a SDS-based DNA extraction method. PCR amplicons were obtained using *GAME36* target site-specific oligonucleotide pairs. These PCR products were purified using a gel extraction kit and sequenced with same primers used above by Sanger sequencing. Superimposed sequenced chromatograms were decoded by using the web tool Snapgene. Oligonucleotides used in this study are presented in Supplementary Data 4.

### Gene expression analysis by quantitative real-Time PCR

Targeted gene expression analysis was performed with three or more biological replicates (n ≥ 3) for each genotype by qRT-PCR. Total RNA was isolated using the Trizol method (Sigma-Aldrich). DNase I (Sigma-Aldrich)-treated RNA was reverse transcribed using a high-capacity cDNA reverse transcription kit (Applied Biosystems). Gene-specific oligonucleotides were designed with Primer BLAST software (NCBI). The *TIP41* gene was used as an endogenous control in gene expression analysis^47^.

### *E. coli* expression and in vitro enzyme assays

Eleven BAHD family candidate genes [from cultivated tomato *cv*. Micro-Tom; listed in Supplementary Fig. 4] were cloned separately into the pET28 vector and expressed in *E. coli* BL21 (DE3) cells. Briefly, bacterial cells were grown in LB medium at 37 °C. When cultures reached OD_600_ = 0.6, protein expression was induced with 200 μM of IPTG at 15 °C, for 20 h. Bacterial cells were lysed by sonication in 50 mM Tris-HCl pH 8.0, 300 mM NaCl, 10% glycerol and SIGMAFAST protease inhibitor tablets (Sigma-Aldrich). Each BAHD soluble protein was purified on Ni-NTA agarose beads (Adar Biotech) and eluted with 250 mM imidazole in a buffer containing 50 mM NaH_2_PO_4_ (pH 7.5) and 150 mM NaCl. Standard BAHD in vitro enzyme assay was performed in 100 µL reaction mix containing 5 µg hydroxytomatine substrate, 50 mM potassium phosphate buffer (pH 7.5), acetyl CoA (200 µM) and purified BAHD enzyme. The reaction mixture was incubated at 30 °C for 2 h. 250 µL methanol containing 0.1% formic acid was added to stop the reaction. After centrifugation for 15 min at 20,000 **×** *g* and filtering through 0.22 μm filters, analysis of enzyme assay products was performed in MRM positive mode using a UPLC-TQ-MS (Waters). Metabolite separation was done on a 100 × 2.1-mm i.d., 1.7-µm UPLC BEH C18 column (Waters Acquity). The mobile phase consisted of 0.1% formic acid in acetonitrile:water (5:95, v/v; phase A) and 0.1% formic acid in acetonitrile (phase B). The following linear gradient was used: from 90 to 70% phase A over 11.5 min, from 70 to 0% phase A over 0.5 min, then held at 100% phase B for 1.5 min; and then returned to the initial conditions (90% phase A) within 0.2 min and conditioning at 90% phase A for 1.3 min. The following MS parameters were applied: capillary voltage 3.0 kV, cone-50 V. MRM transitions used: hydroxytomatine (1050.50 > 414.30, 1050.50 > 594.40; collision for both 50 eV) and acetoxytomatine (1092.50 > 414.30 collision 50 eV, 1092.50 > 798.40 collision 25 eV).

For the Coupled in vitro GAME31 and GAME36 enzyme assays, *SlGAME31* and *SlGAME36* (from cultivated tomato *cv*. Micro Tom) and *SpGAME36* (from *S. pennellii*), were cloned separately into the pET28b vector and expressed in *E. coli* BL21 (DE3) cells. As we could not manage to amplify *SmGAME36* from any tissues of cultivated eggplant, a synthetic gene was obtained from Twist Bioscience and, further used for protein expression. Three steroidal alkaloids; tomatidine, *α*-tomatine and *α*-solamargine were used as substrates in enzyme assays. The recombinant SlGAME31 enzyme activity assay was performed according to Cárdenas et al.^11^ without any modifications. Briefly, the standard full reaction (100 µL) consisted of 10 mM L-ascorbic acid, 10 mM *α*-ketoglutaric acid, 500 µM FeSO_4_, 5 µg substrate, 50 mM potassium phosphate buffer (pH 7.5) and purified SlGAME31 enzyme. The reaction mixture was incubated 30 °C for 3 h. While performing coupled enzyme assay that includes GAME36, acetyl CoA (200 µM) was added to standard assay mixture as acyl donor. After incubation, the reaction was mixed with 250 µL methanol containing 0.1% formic acid, extracted, and analyzed by UPLC-qTOF-MS using standard (40 min.) linear gradient conditions as above. Individual or coupled enzyme assay products were identified based on accurate mass-derived elemental composition and MS/MS fragmentation pattern generated in this study (Supplementary Data 3). Protein extracts obtained from empty pET28 vector-transformed *E. coli* BL21 (DE3) cells were used in control reactions.

### In vitro reconstruction of the Esculeoside A biosynthetic pathway

*E. coli* expression of SlGAME40 and SlGAME5 (from cultivated tomato *cv*. Micro Tom) was performed in a similar way to the one described above for other GAME enzymes. One tube assay of recombinant GAME31, GAME36, GAME40, GAME5 enzymes with *α*-tomatine was carried out under standard assay conditions, as described above with the addition of UDP-glucose to the standard assay mixture. Sequential reconstitution assays were performed using the respective GAME enzymes, cofactors and *α*-tomatine substrate. LC-MS analysis of assay products (Esculeoside A pathway intermediates) was carried out in MRM positive mode using a UPLC-TQ-MS (Waters) similarly as described above. The following MRM transitions were used: *α*-tomatine (1034.50 > 416.40, 1034.50 > 578.40; collision for both 50 eV), hydroxytomatine (1050.50 > 414.30, 1050.50 > 594.40; collision for both 50 eV), acetoxytomatine (1092.50 > 414.30 collision 50 eV, 1092.50 > 798.40 collision 25 eV), acetoxy-hydroxytomatine (1108.55 > 430.33 collision 50 eV, 1108.55 > 490.35 collision 30 eV), Esculeoside A (1270.61 > 754.44, 1270.61 > 814.46; collision for both 60 eV). Relative quantification was done using the TargetLynx program (Waters), using the sum of two MRM transitions.

### Reconstruction of Esculeoside A biosynthesis in *N. benthamiana*

For transient overexpression, *SlGAME31*, *SlGAME36*, *SlGAME40*, and *SlGAME5* were cloned into destination vector 3Ω1 using Goldenbraid cloning^48^ and transformed into *Agrobacterium tumefaciens* (GV3101) by electroporation. Single clones with each target construct were inoculated into 10 ml of LB medium supplemented with antibiotics (250 μg ml^−1^ spectinomycin and 50 μg ml^−1^ gentamicin) and cultures were grown overnight at 28 °C with shaking (200 rpm). Cells were centrifuged at 2,000 **×** *g* for 15 min and the pellet was resuspended in 5 ml of infiltration buffer [100 mM MES buffer (pH 5.6), 10 mM MgCl_2_, 100 μM acetosyringone]. After another round of centrifugation, the pellet was resuspended again in 10 ml of infiltration buffer and incubated at room temperature for 2 h. Agrobacterium suspensions (OD_600_ = 0.3 for each strain) were infiltrated into 4–6-week-old *N. benthamiana* leaves. After 3 days post infiltration, the substrate *α*-tomatine (50 µg ml^−1^) was injected into infiltrated leaves. After 48 h, leaves were collected for further LC-MS based SGA analysis. Biological replicates consisted of several leaves collected from different infiltrated plants. Analysis of SGAs was performed in UPLC-qTOF-MS using standard (40 min.) linear gradient conditions as described above. Various *GAME* genes combinations (as shown in Supplementary Fig. 15) were included in the transient expression experiments to test the promiscuity of GAME enzymes on Esculeoside A pathway intermediates.

### Phylogenetic and synteny analysis

Putative BAHDs sequences from tomatoes including GAME36, related GAME36 sequences from wild tomato species and other *Solanum* species were obtained from NCBI and public databases. Sequence alignments were performed using ClustalOmega^49^. The Maximum Likelihood tree was inferred in MEGAX^50^ with the following parameters: 1000 bootstrap replications, Poisson model, discrete gamma distribution (five categories), and partial deletion. Evolutionary distances are in units of a number of amino acid substitutions per site. Amino acid sequences used in the phylogenetic analysis are provided in Supplementary Data 2. To identify the syntenic relationship between tomato, eggplant, and *S. pennellii* genomes, we used MCscan tool from the JCVI utility library^51^ (https://github.com/tanghaibao/jcvi/wiki/MCscan-(Python-version). Regions corresponding to *GAME36* and *GAME36-like* from chromosome 8 were used to prepare Supplementary Fig. 18.

### Biochemical characterization of tomato GAME36

In order to continue biochemical assays and kinetics experiments, we needed larger quantities of hydroxytomatine and hydroxysolamargine substrates. To generate these substrates, we used SlGAME31 enzyme that convert *α*-tomatine and *α*-solamargine to hydroxytomatine and hydroxysolamargine respectively, as described in “*E. coli* expression and in vitro enzyme assays” section. Briefly, each assay reaction containing approximately 1 mg of the SGA substrate (i.e. *α*-tomatine or *α*-solamargine) was carried out for 10 minutes using purified tomato GAME31 enzyme and required co-factors, after that it was stopped with two volumes of acetonitrile, frozen in liquid nitrogen and lyophilized. Dry residues were resuspended in 60% methanol, analyzed on LC-MS and quantified based on calibration curves for *α*-tomatine and *α*-solamargine. Respective samples were diluted with 60% methanol to final concentration of 100 µg ml^−1^.

To test the SlGAME36 activity with various acyl-CoAs, two sets of enzyme assays were performed. First coupled enzyme assays with GAME36 and GAME31 were carried out using *α*-tomatine as substrate as described above, where each acyl CoA (0.2 mM) was added to the standard assay mixture as an acyl donor. After incubation, the reaction was mixed with 250 µl methanol containing 0.1% formic acid, extracted, and analyzed by UPLC-qTOF-MS using standard (40 min) linear gradient conditions as above. In the second experiment, an equimolar mixture of acyl-CoAs (acetyl-, butyryl-, isovalyryl-, hexanoyl-, and octanoyl-) each at 0.2 mM were included in the 100 µl enzymatic reaction with either substrate (hydroxytomatine or hydroxysolamargine) and 2 µl of recombinant SlGAME36 (0.2 µg µl^−1^). Each assay reaction was monitored at five-time points (5, 10, 20, 40, 60 min). Assay conditions and further assay product analysis by LC-MS were described in “*E. coli* expression and in vitro enzyme assays” section. Peak areas of each acylated product were normalized to the sum of all detected acylated SGA metabolites at given time point of assay reaction, and plotted as a percentage.

For kinetic analysis of GAME36, we tested two different acyl donors (acetyl and butyryl CoA) and two acceptors (hydroxytomatine and hydroxysolamargine). To measure kinetic parameters for acyl-CoAs for GAME36, 100 µM hydroxytomatine was used as the acyl acceptor substrate, with variable concentrations of acyl-CoAs (0–1000 µM). To measure kinetics parameters for acyl acceptors, 500 µM acetyl CoA was used as a donor with varying hydroxytomatine or hydroxysolamargine substrate concentrations (0–100 µM). Each reaction (50 µl) included 1 µl of recombinant SlGAME36 (0.2 µg µl^−1^), appropriate amount of acceptor dissolved in 2.5 µl of methanol and other assay components as described in ‘*E. coli* expression and in vitro enzyme assays’ section. The reactions were run at 30 °C, and stopped by the addition of two volumes of acetonitrile. Samples were centrifuged for 10 minutes at 20,000 × *g*, supernatant was placed in a LC vial. All reactions were done in triplicate and assay products were analyzed by LC-MS using standard (40 min) gradient. Kinetics parameters (*K*_m_, *k*_cat_, *V*_max_) were calculated using the non-linear regression model in GraphPad Prism 8.0 software.

For test of reversibility of reactions catalyzed by tomato GAME36, first we generated acetoxytomatine in a 450 μl reaction with 1.8 µg SlGAME36 purified enzyme in 50 mM potassium phosphate buffer, pH 7.5, 50 μM acetyl CoA and 270 ng hydroxytomatine incubated for 2 h at 30 °C. Reaction was stopped with two volumes of methanol, and purified on SPE column (Strata-X 33 u polymeric reversed phase, Phenomenex). Acetoxytomatine purity was confirmed using LC-MS and then used in reversible reaction assays (each 50 μl) with 0.2 µg of SlGAME36 enzyme in 50 mM potassium phosphate buffer, pH 7.5 supplemented with 50 μM CoA. Reactions were set in triplicate with incubation for 2 h at 30 °C and stopped by addition of two volumes of methanol with 0.1% formic acid. Negative control reaction was performed without SlGAME36 enzyme. Samples were analyzed by LC-MS. Conversion of acetoxytomatine to hydroxytomatine in the assay was assessed by measuring the increase of hydroxytomatine accumulation. As acetoxytomatine used in the ‘reversible’ reaction assay contained traces of hydroxytomatine, fold change of hydroxytomatine peak area between negative control and assay with GAME36 is presented.

### Wild tomato accession analysis

Seeds of *S. arcanum* (LA2172) and *S. huaylasense* (LA1364, LA1365, LA1983) were kindly provided by Prof. Antonio Granell Richart. Seeds of *S. peruvianum* (LA1278, LA1954) were obtained from Tomato Genetics Resource Centre (University of California, Davis). Wild tomato accessions and cultivated tomato (*Solanum lycopersicum cv*. Moneymaker) were grown in a climate-controlled greenhouse (at Max Planck Institute for Chemical Ecology, Jena, Germany) at 24 °C during the day and 18 °C during night, with natural light. GAME36 coding sequences from different wild accessions were amplified using cDNA reverse transcribed from RNA (extracted from 6–8 week-old young leaves). The primers used for amplification are provided in Supplementary Data 4. The resulting PCR products were cloned into binary 3Ω1 vector using Goldenbraid cloning^48^. *GAME36* gene sequences were obtained by Sanger sequencing of several 3Ω1 clones for each wild tomato accession. Sequencing chromatograms from each wild accession were compared to cultivated tomato (*S. lycopersicum cv*. Moneymaker) ones. Hydroxytomatine and acetoxytomatine SGAs extraction from leaves of different wild accessions was performed with the same methods described above (see “Plant extracts preparation and LC-MS based SGA analysis”). LC-MS analysis for this set of samples was done on different instruments as described here: samples were analyzed using a Thermo Scientific UltiMate 3000 ultra-high performance liquid chromatography (UHPLC) system coupled to an Impact II UHR-Q-ToF (Ultra-High Resolution Quadrupole-Time-of-Flight) mass spectrometer (Bruker Daltonics) with the standard (43 min, positive mode) run conditions as follows: 5% B for 1 min; 5% B to 28% B in 22 min; then changing to 100% B in 14 min and further at 100% B for 3 min, and finally returned to the initial conditions (5% phase B) within 0.5 min. The column was equilibrated with 5% B for another 2.5 min before the next injection. Separation of metabolites was performed on a 100 × 2.1-mm, 1.7-µm Acquity UPLC C18 column (Waters, MA, USA). The mobile phase consisted of 0.1% formic acid in water (phase A) and acetonitrile (phase B). The flow rate was 0.3 ml min^−1^, and the column temperature was kept at 35 °C. Mass spectrometry was performed in positive electrospray ionization mode (capillary voltage = 4000 V; end plate offset = 500 V; nebulizer pressure = 2.2 bar; drying gas: nitrogen at 250 °C and 10 L min^−1^). Mass spectrometry data were recorded at 12 Hz ranging from 100 to 1200 *m/z* in auto MS/MS mode with an active exclusion window of 0.2 min. Fragmentation was triggered on an absolute threshold of 400 and restricted to a total cycle time range of 0.5 s, with dynamic collision energy (20-50 eV). To calibrate MS spectrum recording, each run was initiated with the direct source infusion of a sodium formate-isopropanol calibration solution (using an external syringe pump at 0.18 ml h^−1^). The initial 1 min of the chromatographic gradient was directed towards the waste. SGA metabolites were putatively identified by comparing their retention times, elemental composition, and mass fragmentation pattern with those described in the literature^10,12,13^. Relative quantification of the SGAs was carried out using Bruker Compass Data Analysis (Version 5.3) software.

### Statistics and reproducibility

Microsoft Excel 2016 and GraphPad Prism 8 software were used for regular statistical analysis and enzyme kinetic analysis. A two-tailed Student’s t-test was used to calculate significant differences among samples or genotypes. Details of biological replicates used in various experiments are provided in the Methods section as well as in Main Figures and Supplementary Figures legends, wherever necessary.

### Reporting summary

Further information on research design is available in the Nature Portfolio Reporting Summary linked to this article.

## Supplementary information

Supplementary Information

Description of Additional Supplementary Files

Supplementray Data 1

Supplementary Data 2

Supplementary Data 3

Supplementary Data 4

Reporting Summary

## Data Availability

Data supporting the findings of this work are available within the paper and its Supplementary Information files. Publicly available RNA-seq data used in our manuscript was retrieved from NCBI Sequence Read Archive with BioProject ID PRJNA307656 and PRJNA798612. Correspondence and requests for materials should be addressed to P.D.S. or A.A. Source data are provided with this paper.

## Source data

Source Data
