# Supplementary material for: A BAHD-type acyltransferase concludes the biosynthetic pathway of non-bitter glycoalkaloids in ripe tomato fruit
Source: Nat Commun. 2023 Jul 27;14:4540. doi: 10.1038/s41467-023-40092-5 (PMC10374582; doi:10.1038/s41467-023-40092-5)
Supplement: Supplementary file 1 — Supplementary Information [file 41467_2023_40092_MOESM1_ESM.pdf]

A BAHD-type Acyltransferase Concludes the Biosynthetic Pathway of Non-bitter Glycoalkaloids in Ripe Tomato Fruit

Sonawane et al.

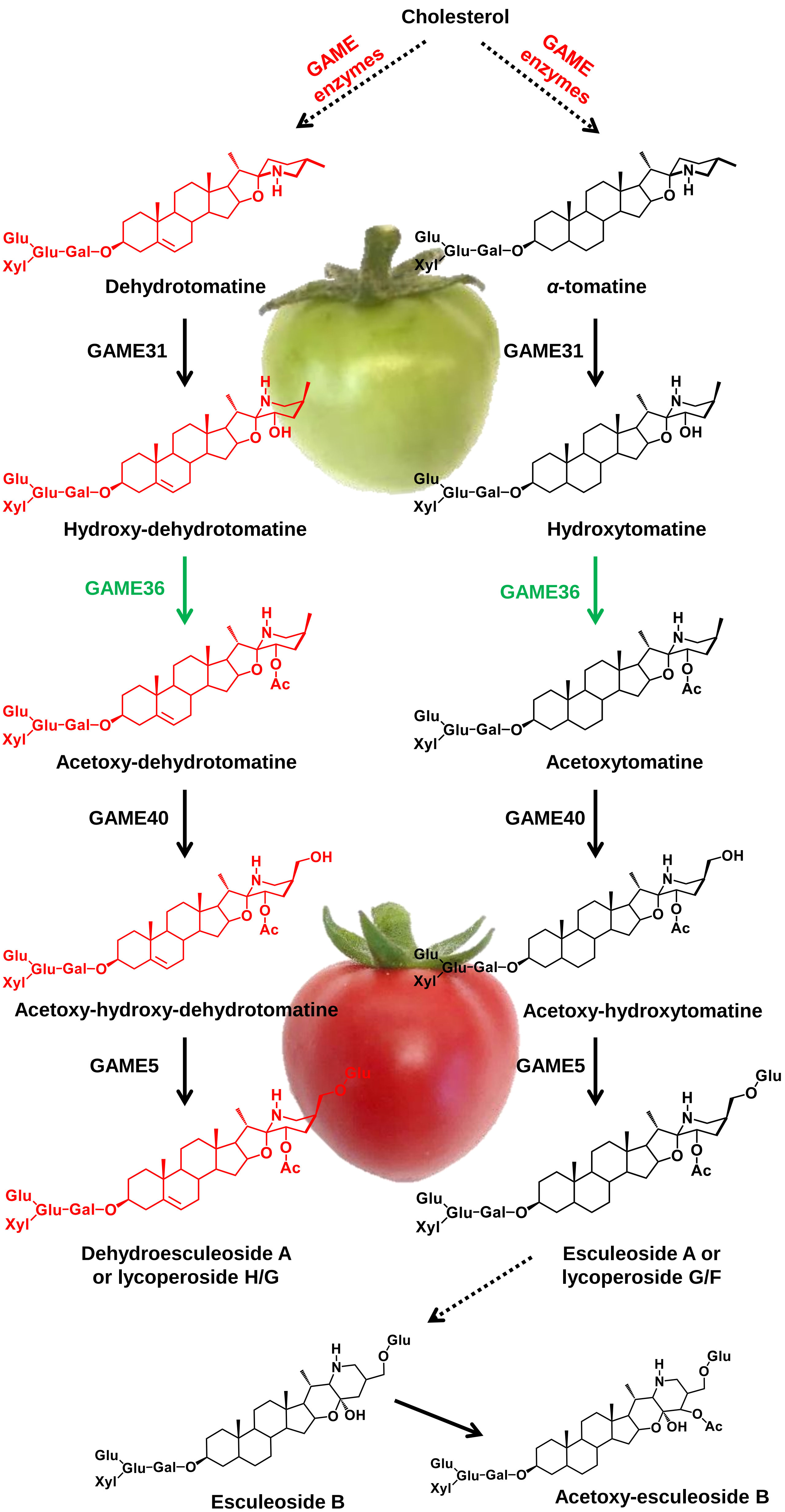

Cholesterol
GAME enzymes
GAME enzymes
Dehydrotomatine
α-tomatine
GAME31
GAME31
Hydroxy-dehydrotomatine
Hydroxytomatine
GAME36
GAME36
Acetoxy-dehydrotomatine
Acetoxytomatine
GAME40
GAME40
Acetoxy-hydroxy-dehydrotomatine
Acetoxy-hydroxytomatine
GAME5
GAME5
Dehydroesculeoside A or lycoperoside H/G
Esculeoside A or lycoperoside G/F
Esculeoside B
Acetoxy-esculeoside B

Supplementary Figure 1: Steroidal glycoalkaloids (SGAs) metabolism during tomato fruit development and ripening. In cultivated tomato, the ripening associated chemical shift in SGAs metabolism involves conversion of *α*-tomatine and dehydrotomatine (major SGAs in green fruit) to Esculeoside A and dehydroesculeoside A (major SGAs in red ripe fruit), respectively. The known SGA biosynthetic enzymes (i.e. GAME31, GAME40 and GAME5) in the Esculeoside A pathway are marked in black. GAME36 enzymatic step discovered in this study is shown in green. Throughout tomato fruit development and ripening, *α*-tomatine derived SGAs (shown in black) are highly abundant as compared to the dehydrotomatine-derived SGAs (marked in red). Dashed arrows represent multiple biosynthetic reactions whereas solid arrows represent a single step. GAME: GLYCOALKALOID METABOLISM; Glu: Glucose; Gal: Galactose; Xyl: Xylose; Rha: Rhamnose and Ac: Acetoxy.

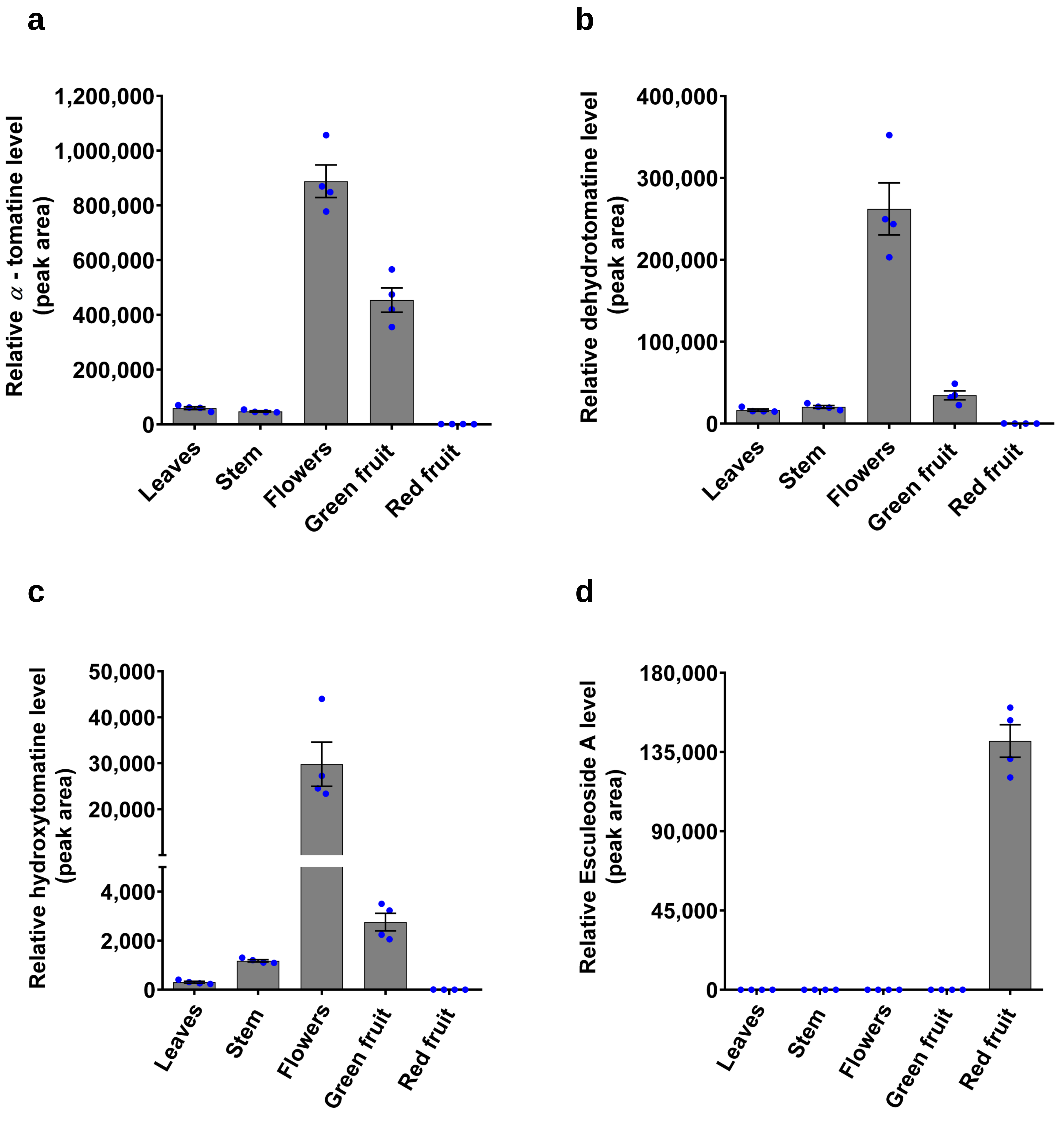

a
Relative α - tomatine level (peak area)
1,200,000
1,000,000
800,000
600,000
400,000
200,000
0
Leaves
Stem
Flowers
Green fruit
Red fruit
b
Relative dehydrotomatine level (peak area)
400,000
300,000
200,000
100,000
0
Leaves
Stem
Flowers
Green fruit
Red fruit
c
Relative hydroxytomatine level (peak area)
50,000
40,000
30,000
20,000
4,000
2,000
0
Leaves
Stem
Flowers
Green fruit
Red fruit
d
Relative Esculeoside A level (peak area)
180,000
135,000
90,000
45,000
0
Leaves
Stem
Flowers
Green fruit
Red fruit

Supplementary Figure 2: Selected SGAs profiling in different tissues of cultivated tomato (*S. lycopersicum cv.* Micro Tom). (a-d) *α*-tomatine (a), dehydrotomatine (b), hydroxytomatine (c) and Esculeoside A (d) content was determined by LC-MS analysis. The peak areas for each SGA were determined using the TargetLynx software. The values indicate means of biological replicates ± standard error mean (n=4).

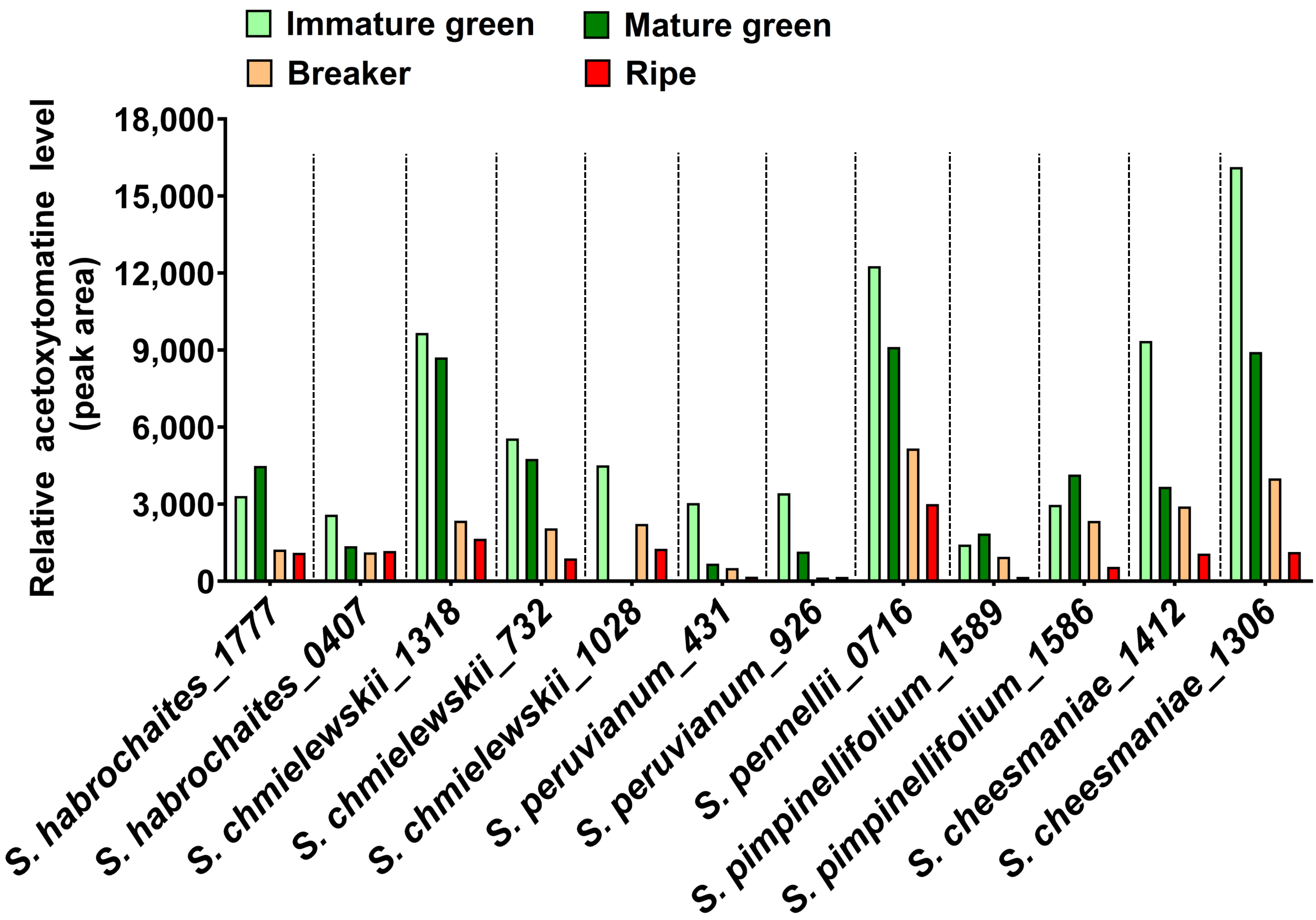
Immature green
Mature green
Breaker
Ripe
Relative acetoxytomatine level (peak area)
18,000
15,000
12,000
9,000
6,000
3,000
0
S. habrochaites_1777
S. habrochaites_0407
S. chmielewskii_1318
S. chmielewskii_732
S. chmielewskii_1028
S. peruvianum_431
S. peruvianum_926
S. pennellii_0716
S. pimpinellifolium_1589
S. pimpinellifolium_1586
S. cheesmaniae_1412
S. cheesmaniae_1306

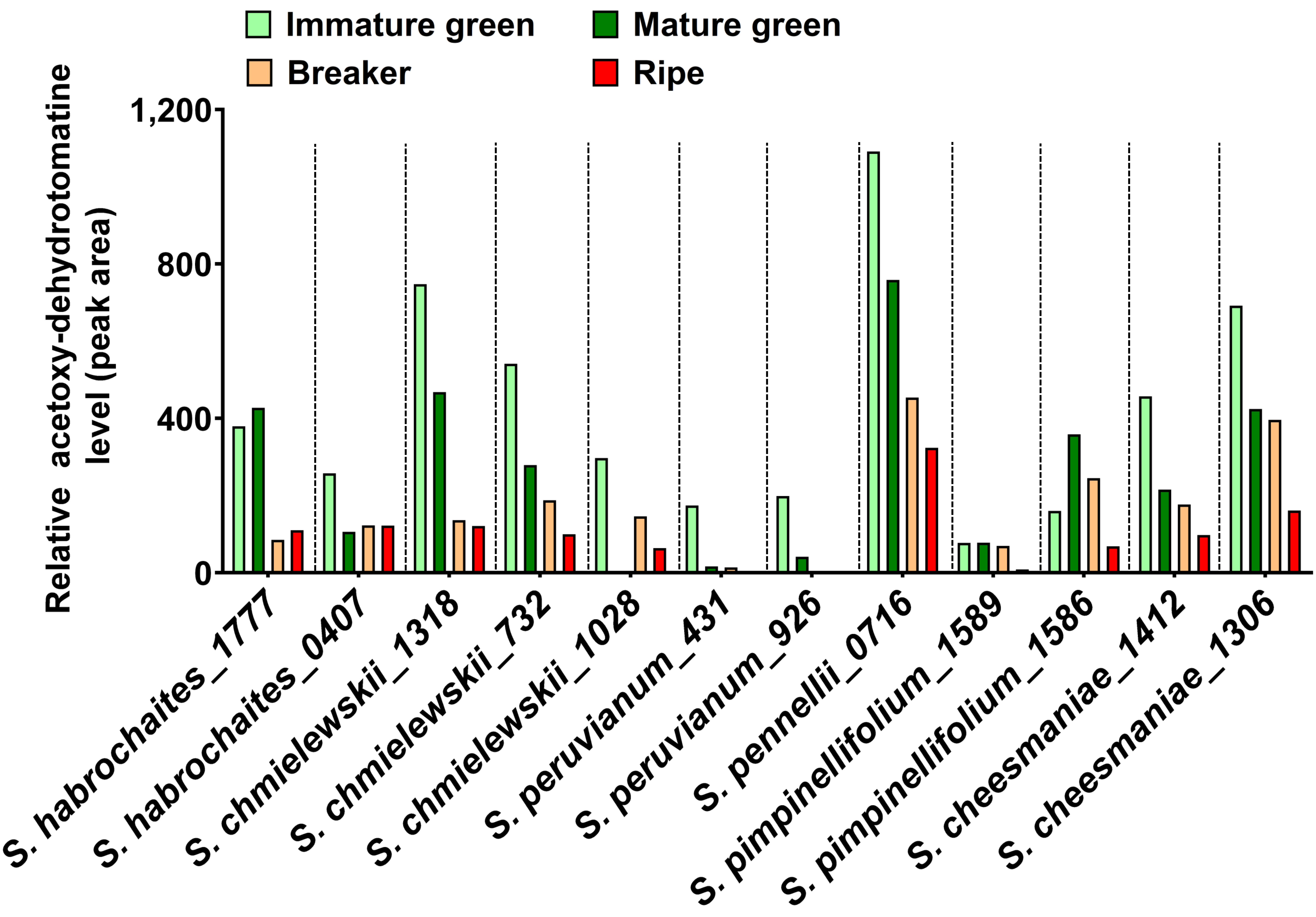
Immature green
Mature green
Breaker
Ripe
Relative acetoxy-dehydrotomatine level (peak area)
1,200
800
400
0
S. habrochaites_1777
S. habrochaites_0407
S. chmielewskii_1318
S. chmielewskii_732
S. chmielewskii_1028
S. peruvianum_431
S. peruvianum_926
S. pennellii_0716
S. pimpinellifolium_1589
S. pimpinellifolium_1586
S. cheesmaniae_1412
S. cheesmaniae_1306

Supplementary Figure 3: Acetoxytomatine and acetoxy-dehydrotomatine levels in wild tomato accessions. SGA analysis was performed in four fruit developmental stages (i.e. immature green, mature green, breaker and ripe) of 12 wild tomato accessions by LC-MS (n=1, single replicate for each fruit ripening stage obtained by extracting several fruits of an individual wild tomato accession). The peak areas were determined using the TargetLynx software.

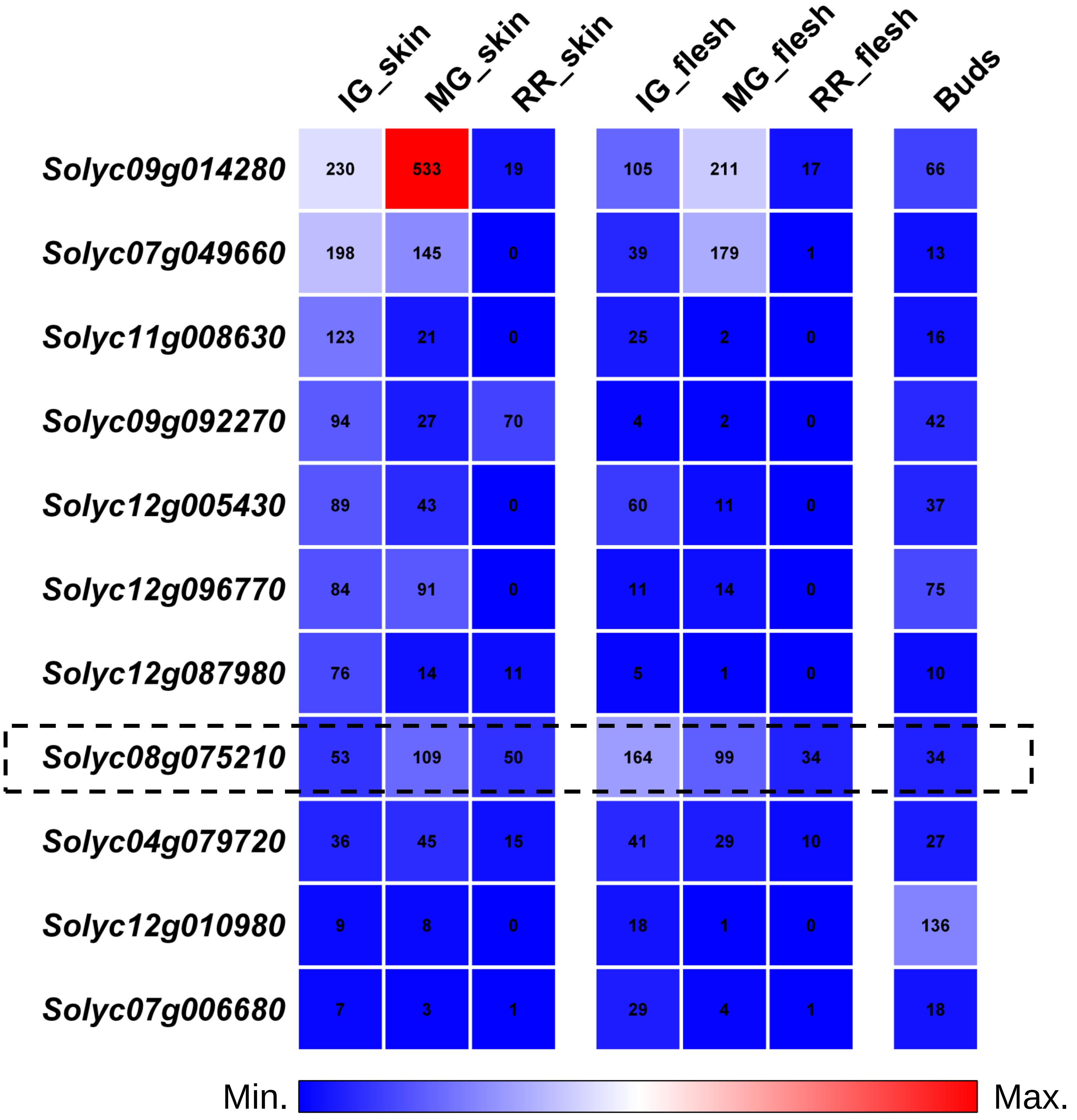
IG_skin
MG_skin
RR_skin
IG_flesh
MG_flesh
RR_flesh
Buds
Solyc09g014280 230 533 19 105 211 17 66
Solyc07g049660 198 145 0 39 179 1 13
Solyc11g008630 123 21 0 25 2 0 16
Solyc09g092270 94 27 70 4 2 0 42
Solyc12g005430 89 43 0 60 11 0 37
Solyc12g096770 84 91 0 11 14 0 75
Solyc12g087980 76 14 11 5 1 0 10
Solyc08g075210 53 109 50 164 99 34 34
Solyc04g079720 36 45 15 41 29 10 27
Solyc12g010980 9 8 0 18 1 0 136
Solyc07g006680 7 3 1 29 4 1 18
Min.
Max.

Supplementary Figure 4: Expression profile of BAHD candidate genes in cultivated tomato (*cv.* Micro Tom) tissue types and fruit developmental stages (RNA-seq expression data). Normalized FPKM (Fragments Per Kilobase of transcript per Million mapped reads) values were used to infer the expression profile. Fruit developmental stages- IG: immature green; MG: mature green; RR: red ripe. The BAHD candidate Solyc08g075210 (GAME36) characterized further in this study is marked in dashed black rectangle.

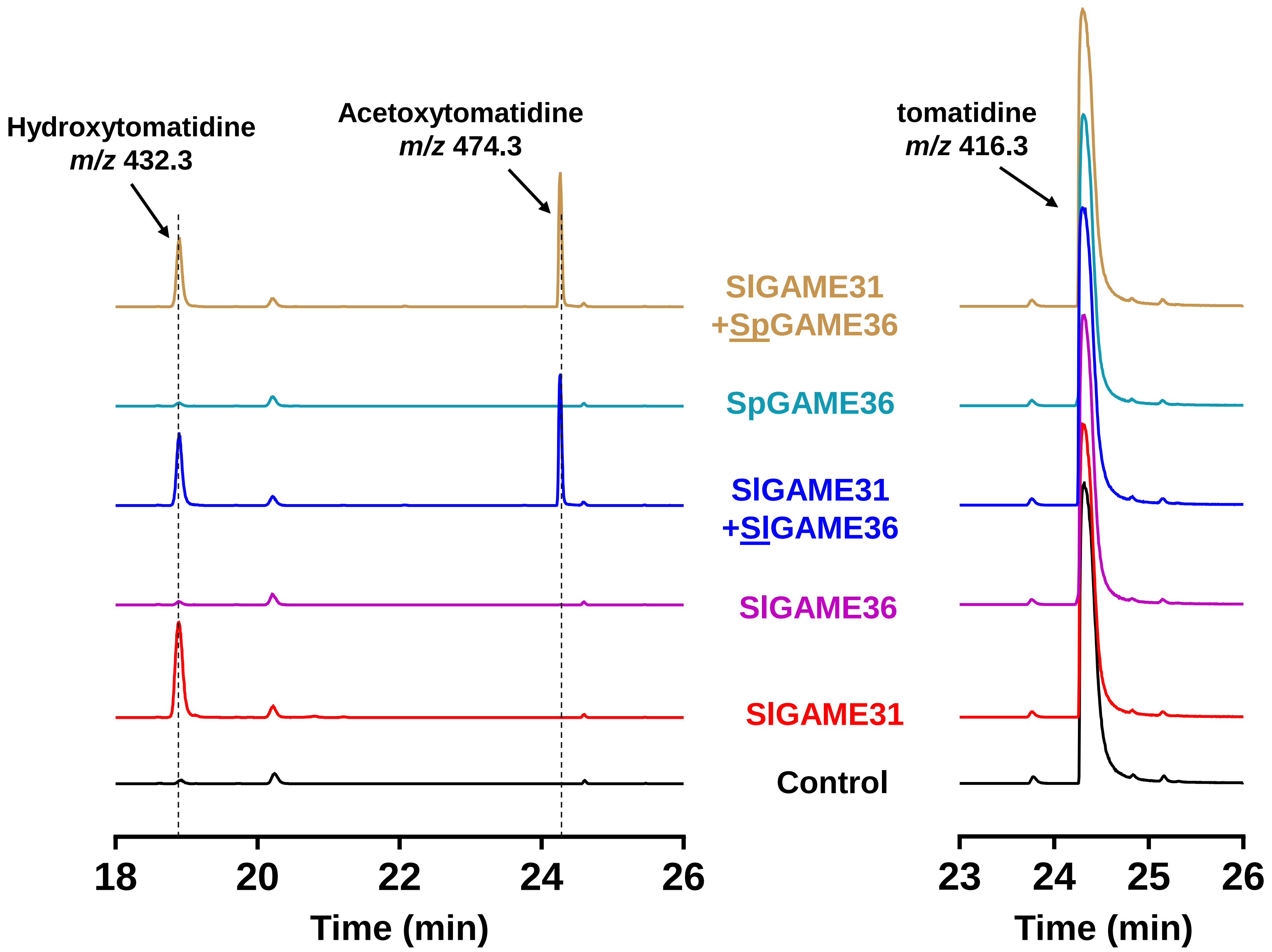
Hydroxytomatidine
m/z 432.3
Acetoxytomatidine
m/z 474.3
tomatidine
m/z 416.3
SlGAME31
+SpGAME36
SpGAME36
SlGAME31
+SlGAME36
SlGAME36
SlGAME31
Control
18
20
22
24
26
Time (min)
23
24
25
26
Time (min)

Supplementary Figure 5: LC-MS analysis of recombinant GAME31 and GAME36 enzyme assay with tomatidine substrate. Recombinant SlGAME36 [*S. lycopersicum* (*cv.* Micro Tom); shown in blue] and SpGAME36 (*S. pennellii*, in tan brown) enzymes convert tomatidine to acetoxytomatidine when assayed together with SlGAME31 (each enzyme produced separately in *E. coli* cells). Control reaction with tomatidine (shown in black) was performed using the protein extracts from *E. coli* cells transformed with empty vector. Individual assays of SlGAME31, SlGAME36 and SpGAME36 recombinant enzymes are shown in red, dark purple and turquoise colors, respectively. Mass to charge (*m/z*) is shown for substrate and assay products. MS/MS analysis and identification of assay products is provided in Supplementary Data 3.

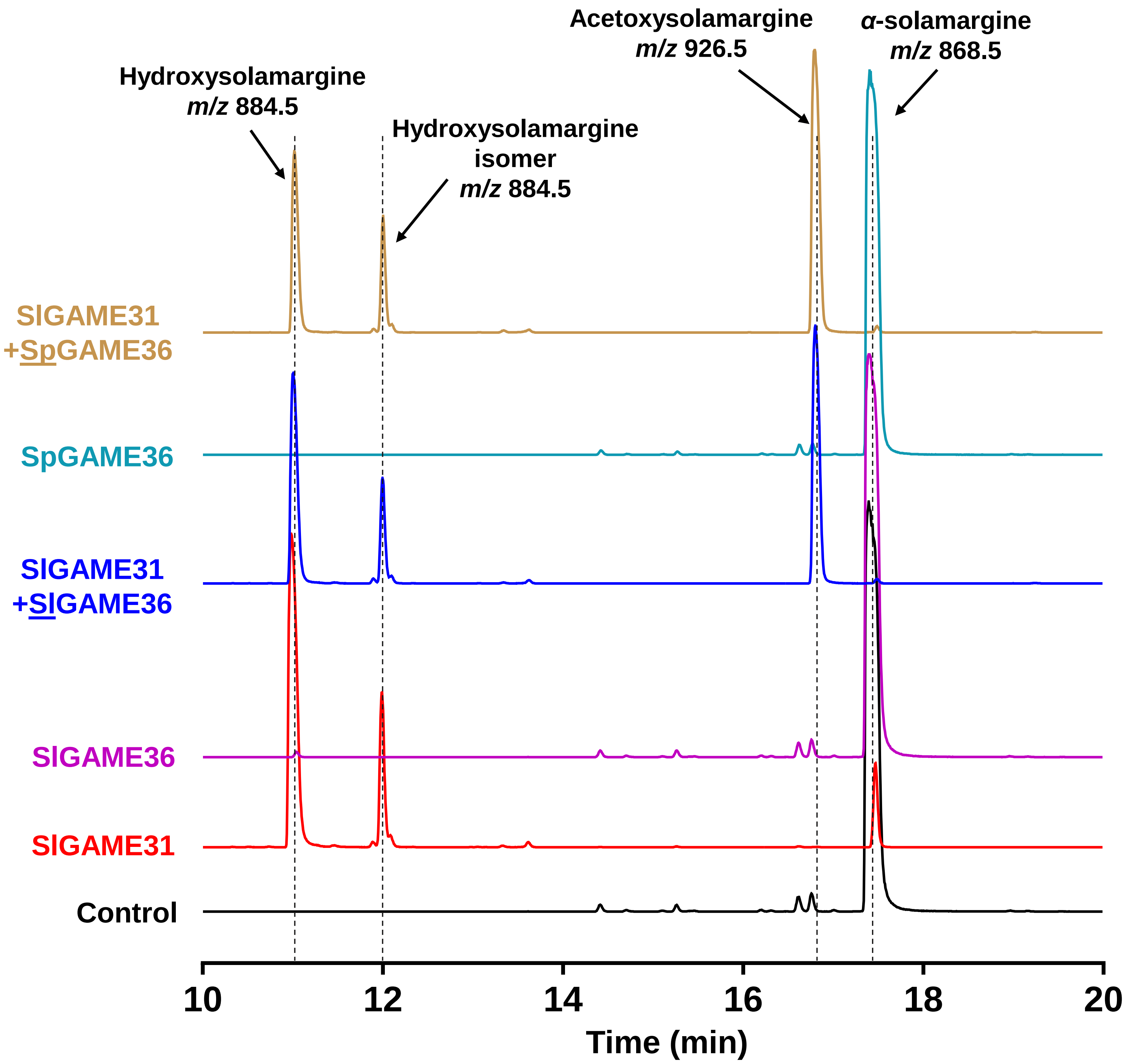

Hydroxysolamargine
m/z 884.5
Hydroxysolamargine
isomer
m/z 884.5
Acetoxysolamargine
m/z 926.5
α-solamargine
m/z 868.5
SlGAME31
+SpGAME36
SpGAME36
SlGAME31
+SlGAME36
SlGAME36
SlGAME31
Control
10
12
14
16
18
20
Time (min)

Supplementary Figure 6: In vitro coupled enzyme assays using recombinant GAME31 and GAME36 proteins. Aligned chromatograms (extracted ion) of assay products (i.e. hydroxysolamargine and acetoxysolamargine) and substrate (*α*-solamargine) are presented here. Blue: Recombinant SlGAME36 and SlGAME31 enzyme assay; Tan brown: Recombinant SpGAME36 and SlGAME31 enzyme assay; Black: control reaction using protein extracts from empty vector transformed *E. coli* cells; other (red, dark purple and turquoise): individual assays of SlGAME31, SlGAME36 and SpGAME36 recombinant enzymes with *α*-solamargine substrate. The recombinant SlGAME31, SlGAME36, SpGAME36 enzymes were produced separately in *E. coli* cells. Enzyme assays analysis was carried out by LC-MS (40 min. run). Mass to charge (*m/z*) is shown for substrate and assay products. MS/MS analysis and identification of assay products is provided in Supplementary Data 3.

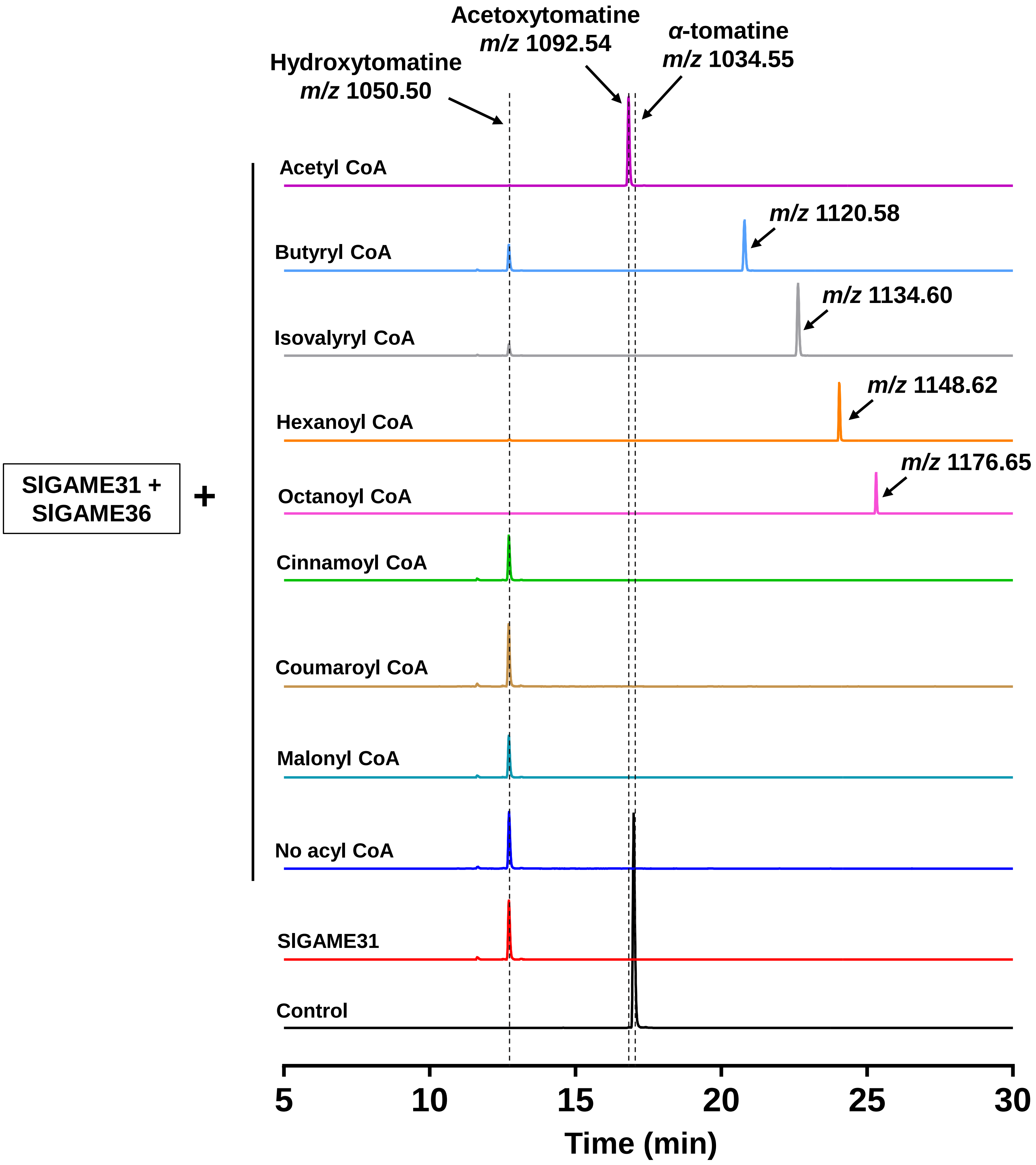
Hydroxytomatine
m/z 1050.50
Acetoxytomatine
m/z 1092.54
α-tomatine
m/z 1034.55
SlGAME31 +
SlGAME36
+
Acetyl CoA
Butyryl CoA
m/z 1120.58
Isovalyryl CoA
m/z 1134.60
Hexanoyl CoA
m/z 1148.62
Octanoyl CoA
m/z 1176.65
Cinnamoyl CoA
Coumaroyl CoA
Malonyl CoA
No acyl CoA
SlGAME31
Control
5
10
15
20
25
30
Time (min)

Supplementary Figure 7: *In vitro* coupled enzyme assays of recombinant GAME31 and GAME36 proteins using different CoA thioesters (acyl donors) as substrates. Aligned chromatograms (extracted ion) of assay products (i.e. acylated SGAs) and substrates (*α*-tomatine, hydroxytomatine) are presented here. Control reaction (in black) was performed using protein extracts from empty vector transformed *E. coli* cells. Enzyme assays analysis was carried out by LC-MS (40 min. run). Mass to charge (*m/z*) is shown for substrate and assay products.

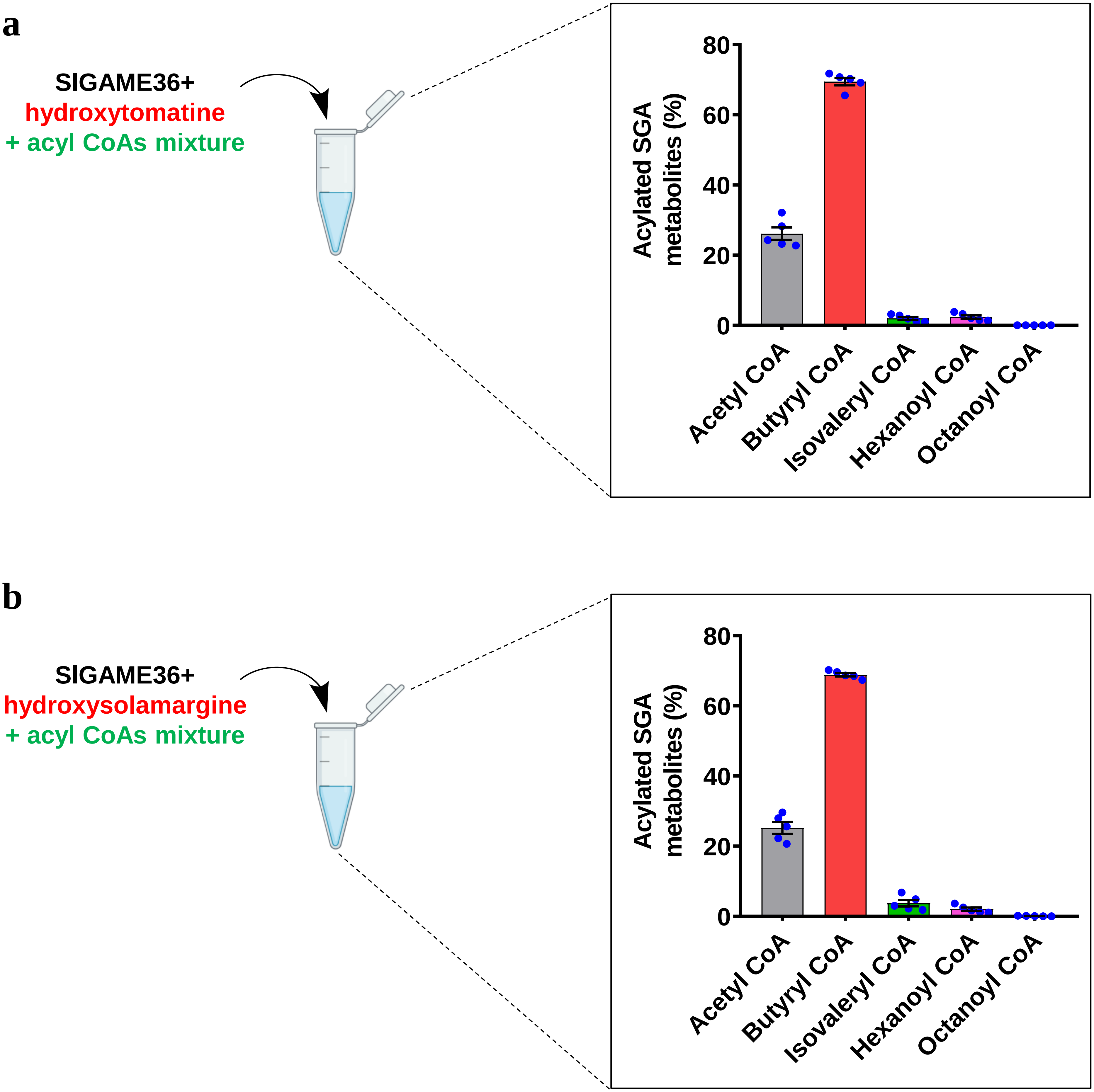
a
SlGAME36+
hydroxytomatine
+ acyl CoAs mixture
Acylated SGA metabolites (%)
0
20
40
60
80
Acetyl CoA
Butyryl CoA
Isovaleryl CoA
Hexanoyl CoA
Octanoyl CoA
b
SlGAME36+
hydroxysolamargine
+ acyl CoAs mixture
Acylated SGA metabolites (%)
0
20
40
60
80
Acetyl CoA
Butyryl CoA
Isovaleryl CoA
Hexanoyl CoA
Octanoyl CoA

Supplementary Figure 8: *In vitro* production of acylated SGAs by GAME36 using different acyl-CoA donors with hydroxytomatine (a) and hydroxysolamargine (b) substrates. Equimolar mixture of acyl-CoAs (acetyl, butyryl, isovalyryl, hexanoyl, and octanoyl) each at 0.2 mM were included in the enzymatic reaction with either substrate (hydroxytomatine and hydroxysolamargine). Each assay reaction was monitored at five different time points (5, 10, 20, 40 and 60 min.), then terminated and extracted after completion of given time point. Peak areas of each acylated product were normalized to the sum of all detected acylated SGA metabolites at given time point of assay reaction, and plotted as a percentage. Values indicates means ± standard error mean (n=5, obtained for each assay reaction from five different time points). Enzyme assays analysis was carried out by LC-MS.

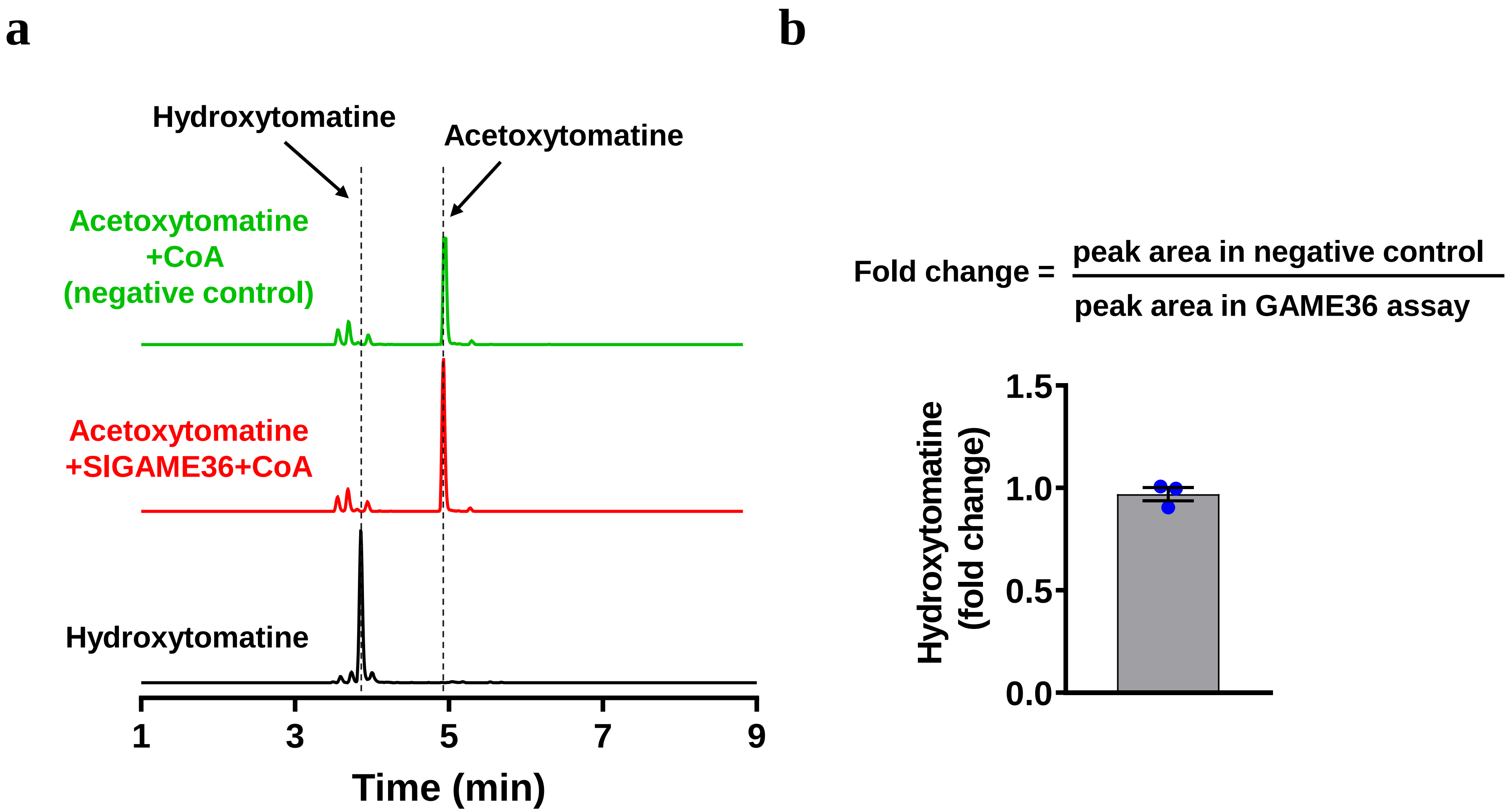
a
Hydroxytomatine
Acetoxytomatine
Acetoxytomatine +CoA (negative control)
Acetoxytomatine +SlGAME36+CoA
Hydroxytomatine
1
3
5
7
9
Time (min)
b
Fold change = peak area in negative control / peak area in GAME36 assay
Hydroxytomatine (fold change)
1.5
1.0
0.5
0.0

Supplementary Figure 9: Reversible reactions test catalyzed by SlGAME36. Recombinant SlGAME36 enzyme did not catalyze reversible reaction when assayed together with acetoxytomatine and CoA. **(a)** Aligned extracted ion chromatograms of expected assay product (hydroxytomatine) and substrate (acetoxytomatine) are presented here. Black: hydroxytomatine; Red: GAME36 assay reaction; Green: negative control reaction using acetoxytomatine and CoA, but without GAME36 enzyme. Enzyme assays analysis was carried out by LC-MS (15 min. run). Mass to charge (*m/z*) is shown for metabolites. **(b)** Hydroxytomatine fold change between negative control and GAME36 enzyme assay. As acetoxytomatine used in the 'reversible' reaction assay contained traces of hydroxytomatine, fold change of hydroxytomatine peak area between negative control and assay with GAME36 is presented. Values in panel b indicates means of fold change ± standard error mean (n=3) obtained from each three independent negative control and GAME36 reactions.

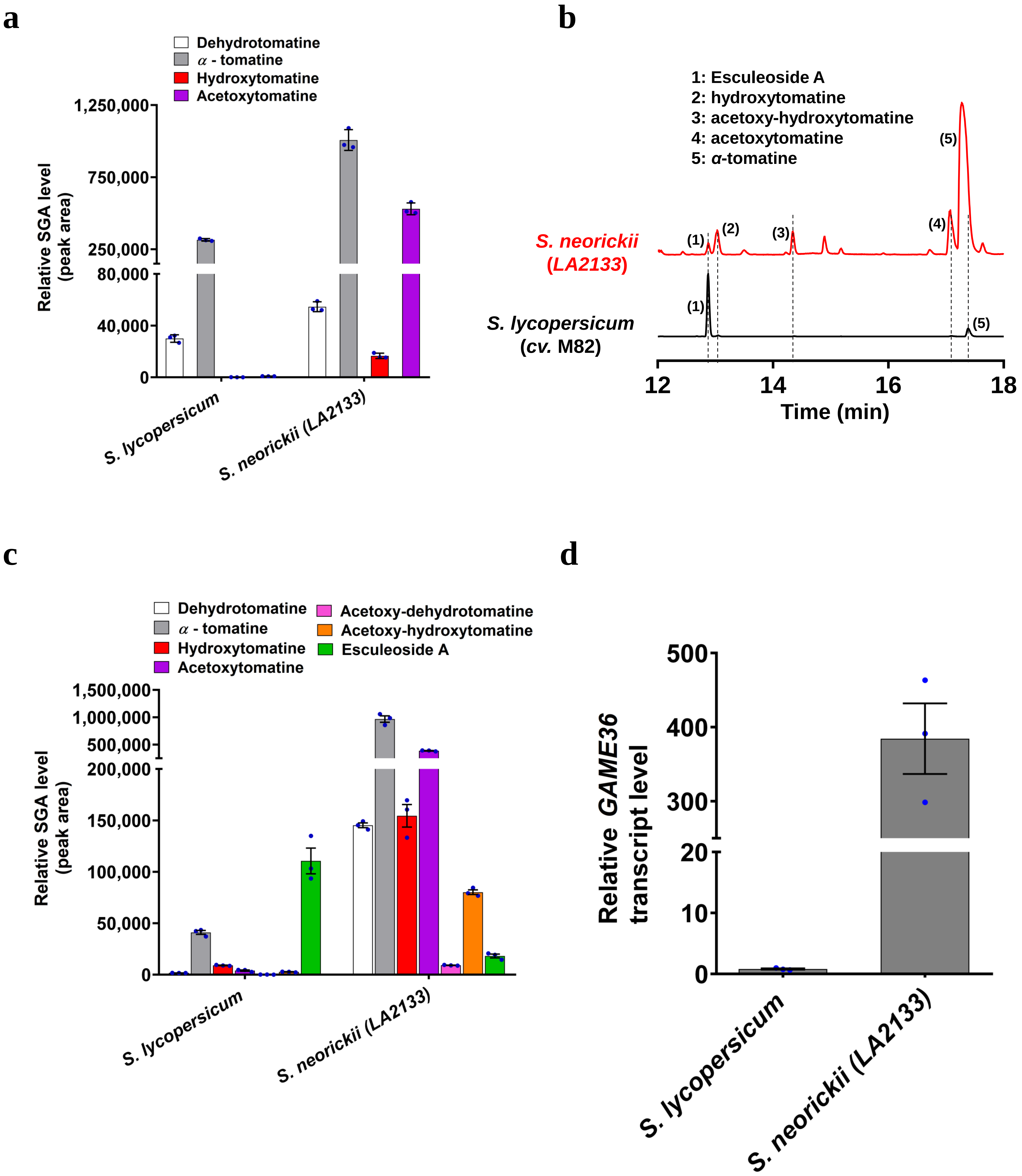
a
Dehydrotomatine
α - tomatine
Hydroxytomatine
Acetoxytomatine
Relative SGA level (peak area)
1,250,000
750,000
250,000
80,000
40,000
0
S. lycopersicum
S. neorickii (LA2133)
b
1: Esculeoside A
2: hydroxytomatine
3: acetoxy-hydroxytomatine
4: acetoxytomatine
5: α-tomatine
S. neorickii (LA2133)
S. lycopersicum (cv. M82)
(1)
(2)
(3)
(4)
(5)
12
14
16
18
Time (min)
c
Dehydrotomatine
α - tomatine
Hydroxytomatine
Acetoxytomatine
Acetoxy-dehydrotomatine
Acetoxy-hydroxytomatine
Esculeoside A
Relative SGA level (peak area)
1,500,000
1,000,000
500,000
200,000
150,000
100,000
50,000
0
S. lycopersicum
S. neorickii (LA2133)
d
Relative GAME36 transcript level
500
400
300
20
10
0
S. lycopersicum
S. neorickii (LA2133)

Supplementary Figure 10: LC-MS based profiling of SGAs and *GAME36* gene expression in wild tomato *S. neorickii* LA2133. (a) *α*-tomatine and downstream SGAs (e.g. hydroxytomatine, acetoxytomatine etc.) content in leaves of *S. neorickii* LA2133 accession and cultivated tomato (*S. lycopersicum cv.* M82). **(b)** Comparison of ripening associated SGA profiles between cultivated tomato and *S. neorickii* LA2133 accession. Representative aligned extracted ion chromatograms for ripe fruit SGAs are shown. **(c)** Ripe fruit SGAs repertoire in cultivated tomato and *S. neorickii* LA2133 accession. Not as previously reported, *S. neorickii* LA2133 accumulates acetoxytomatine and downstream intermediates in the Esculeoside A pathway. LC-MS was used for targeted SGA profiling. **(d)** *GAME36* gene expression in leaves of cultivated tomato (*cv.* M82) and wild tomato *S. neorickii* LA2133 accession as determined by qRT-PCR. Values in panels (a, c and d) represent the means of three biological replicates ± standard error mean (n=3).

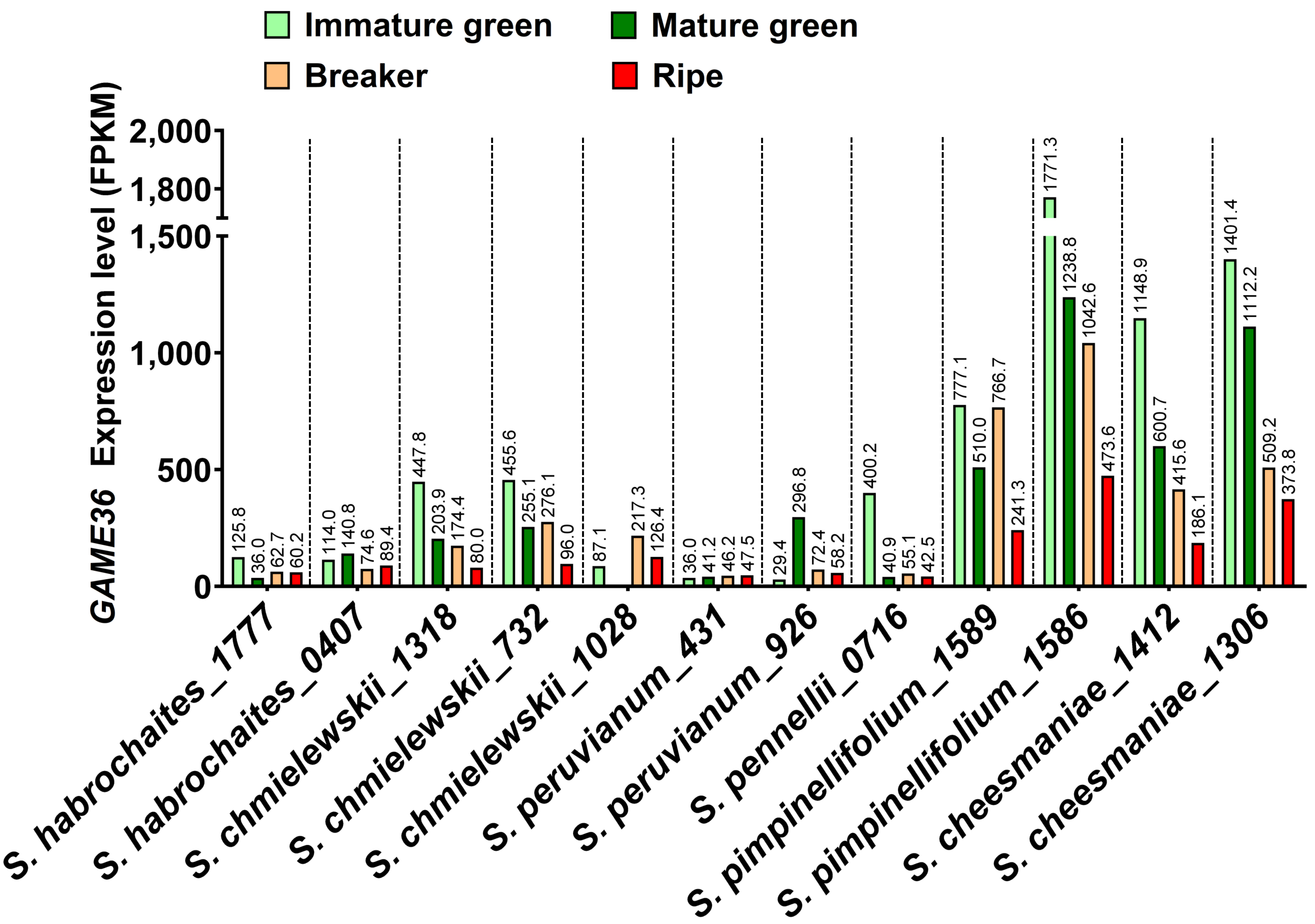

Immature green
Mature green
Breaker
Ripe
GAME36 Expression level (FPKM)
2,000
1,800
1,500
1,000
500
0
125.8
36.0
62.7
60.2
114.0
140.8
74.6
89.4
447.8
203.9
174.4
80.0
455.6
255.1
276.1
96.0
87.1
217.3
126.4
36.0
41.2
46.2
47.5
29.4
296.8
72.4
58.2
400.2
40.9
55.1
42.5
777.1
510.0
766.7
241.3
1771.3
1238.8
1042.6
473.6
1148.9
600.7
415.6
186.1
1401.4
1112.2
509.2
373.8
S. habrochaites_1777
S. habrochaites_0407
S. chmielewskii_1318
S. chmielewskii_732
S. chmielewskii_1028
S. peruvianum_431
S. peruvianum_926
S. pennellii_0716
S. pimpinellifolium_1589
S. pimpinellifolium_1586
S. cheesmaniae_1412
S. cheesmaniae_1306

Supplementary Figure 11: *GAME36* expression levels in four fruit developmental stages of wild tomato accessions (normalized RNA-seq data). FPKM: Fragments Per Kilobase of transcript per Million mapped reads.

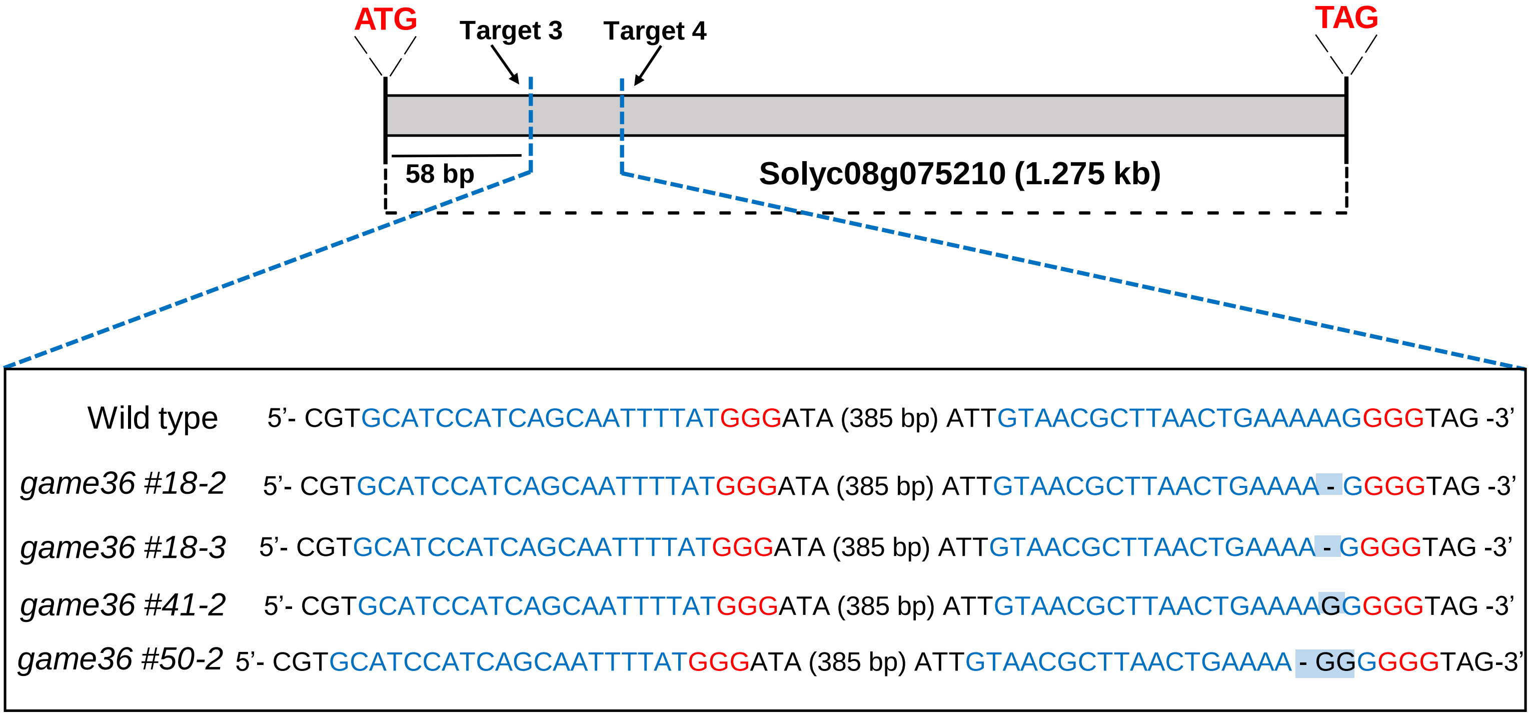

ATG
Target 3
Target 4
TAG
58 bp
Solyc08g075210 (1.275 kb)
Wild type 5'- CGTGCATCCATCAGCAATTTTATGGGATA (385 bp) ATTGTAACGCTTAACTGAAAAAGGGGTAG -3'
game36 #18-2 5'- CGTGCATCCATCAGCAATTTTATGGGATA (385 bp) ATTGTAACGCTTAACTGAAAA - GGGGTAG -3'
game36 #18-3 5'- CGTGCATCCATCAGCAATTTTATGGGATA (385 bp) ATTGTAACGCTTAACTGAAAA - GGGGTAG -3'
game36 #41-2 5'- CGTGCATCCATCAGCAATTTTATGGGATA (385 bp) ATTGTAACGCTTAACTGAAAAGGGGGTAG -3'
game36 #50-2 5'- CGTGCATCCATCAGCAATTTTATGGGATA (385 bp) ATTGTAACGCTTAACTGAAAA - GGGGGTAG-3'

Supplementary Figure 12: Schematic representation of *GAME36* with location of guide RNA sequences, CRISPR-Cas9 cleavage sites and *game36* mutant sequences. PAMs and inserted bases are shown in red and blue respectively and deleted bases are replaced by a dash. Four independent (#14, #18, #41 and #50) homozygous *game36* mutant lines were generated in tomato (*cv.* Micro Tom) background.

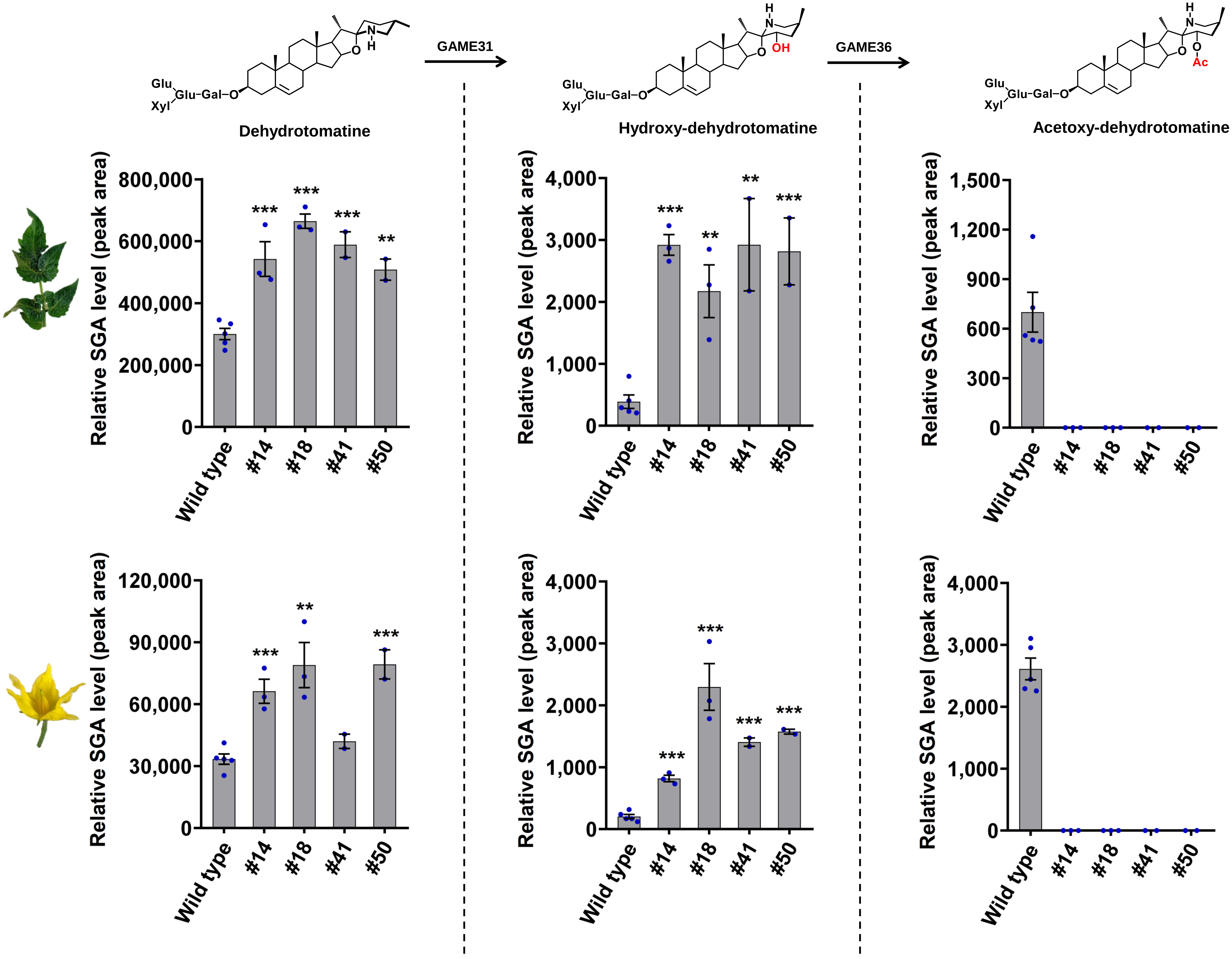

GAME31
GAME36
Dehydrotomatine
Hydroxy-dehydrotomatine
Acetoxy-dehydrotomatine
Relative SGA level (peak area)
Wild type
#14
#18
#41
#50

Supplementary Figure 13: Levels of dehydrotomatine-derived SGAs detected in leaves and flowers of *game36* mutant lines as compared to wild type ones. Acetoxy-dehydrotomatine was not detected in the *game36* mutant lines. Four independent (#14, #18, #41 and #50) homozygous *game36* mutant lines were generated in tomato (cv. Micro Tom) background. The values indicate means of biological replicates ± standard error mean (n=5 for wild type, n=3 for #14 and #18 lines, and n=2 for #41 and #50 lines genotype). Asterisks indicate significant changes compared to wild type samples as calculated by two tailed Student's t-test (*P-value < 0.05; **P-value < 0.01; ***P-value < 0.001). LC-MS was used for targeted profiling of SGAs.

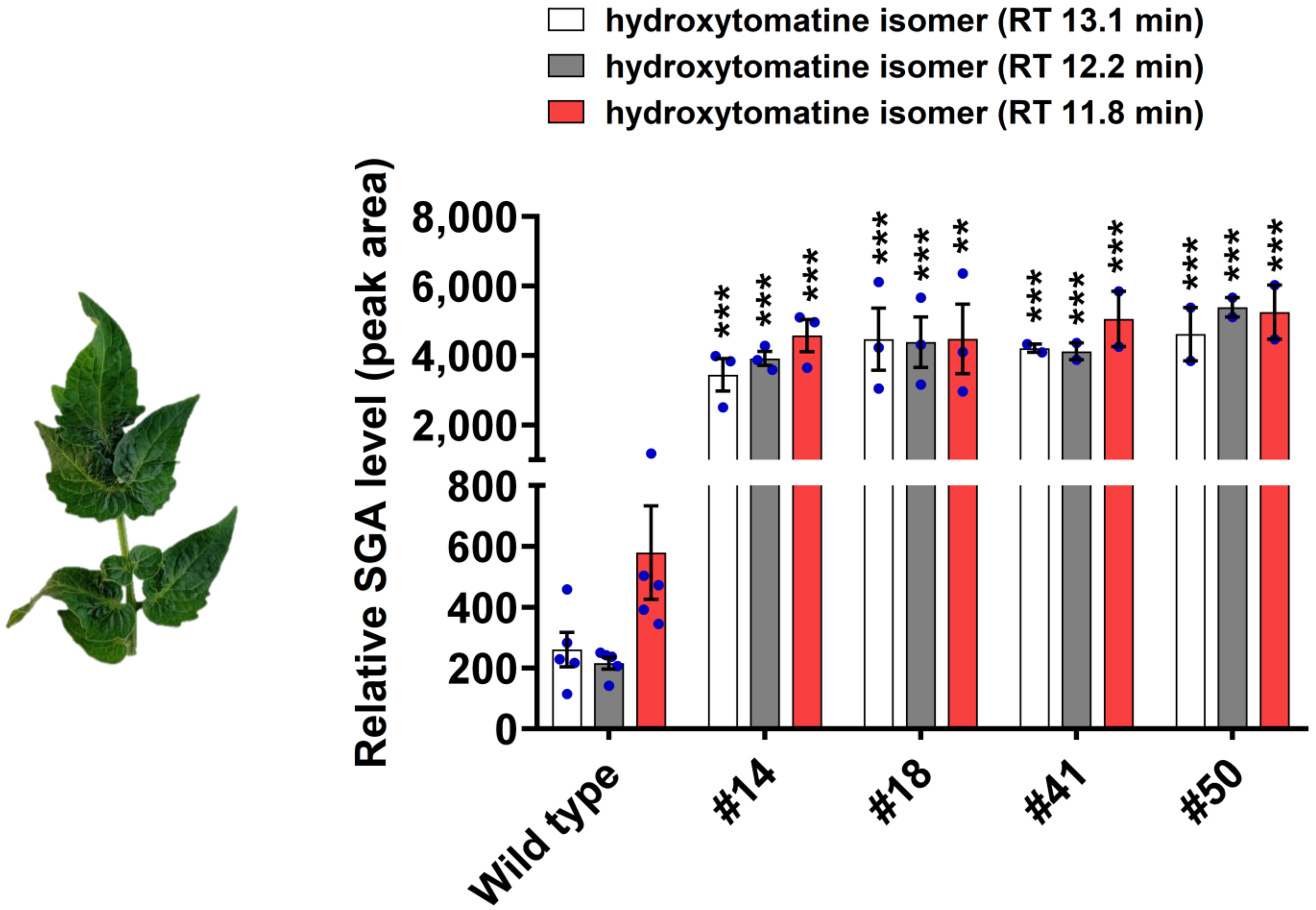
hydroxytomatine isomer (RT 13.1 min)
hydroxytomatine isomer (RT 12.2 min)
hydroxytomatine isomer (RT 11.8 min)
Relative SGA level (peak area)
8,000
6,000
4,000
2,000
800
600
400
200
0
Wild type
#14
#18
#41
#50

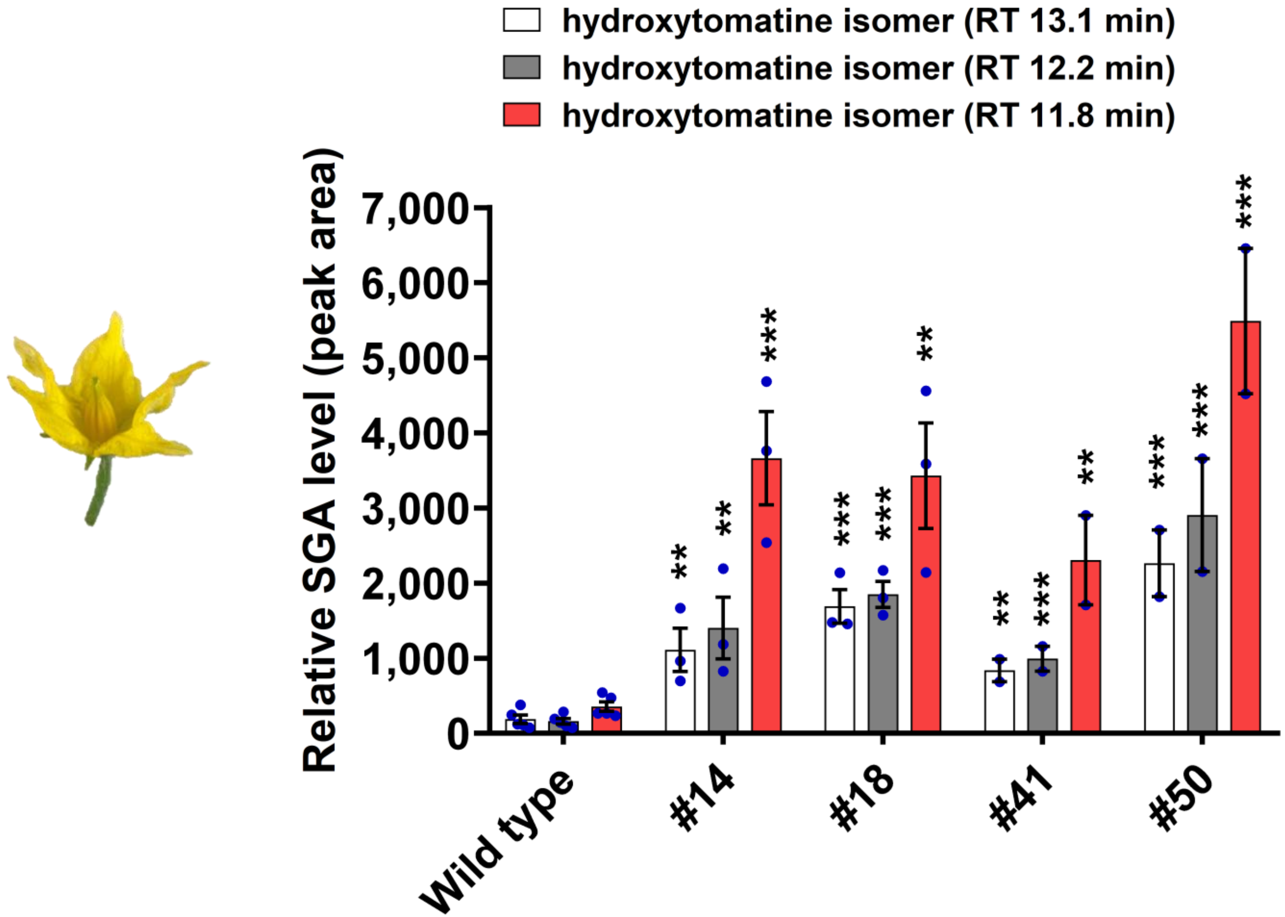
hydroxytomatine isomer (RT 13.1 min)
hydroxytomatine isomer (RT 12.2 min)
hydroxytomatine isomer (RT 11.8 min)
Relative SGA level (peak area)
7,000
6,000
5,000
4,000
3,000
2,000
1,000
0
Wild type
#14
#18
#41
#50

Supplementary Figure 14: Levels of hydroxytomatine isomers in *game36* mutant tomato leaves (upper panel) and flowers (lower panel). Knockout of *game36* in cultivated tomato resulted in significant accumulation of hydroxytomatine and its isomers. These isomers are typically present in minor quantities in wild type tissues. Four independent (#14, #18, #41 and #50) homozygous *game36* mutant lines were generated in tomato (*cv.* Micro Tom) background. The values indicate means of biological replicates ± standard error mean (n=5 for wild type, n=3 for #14 and #18 lines, and n=2 for #41 and #50 lines genotype). Asterisks indicate significant changes compared to wild type samples as calculated by two tailed Student's t-test (*P-value < 0.05; **P-value < 0.01; ***P-value < 0.001). LC-MS was used for SGAs analysis.

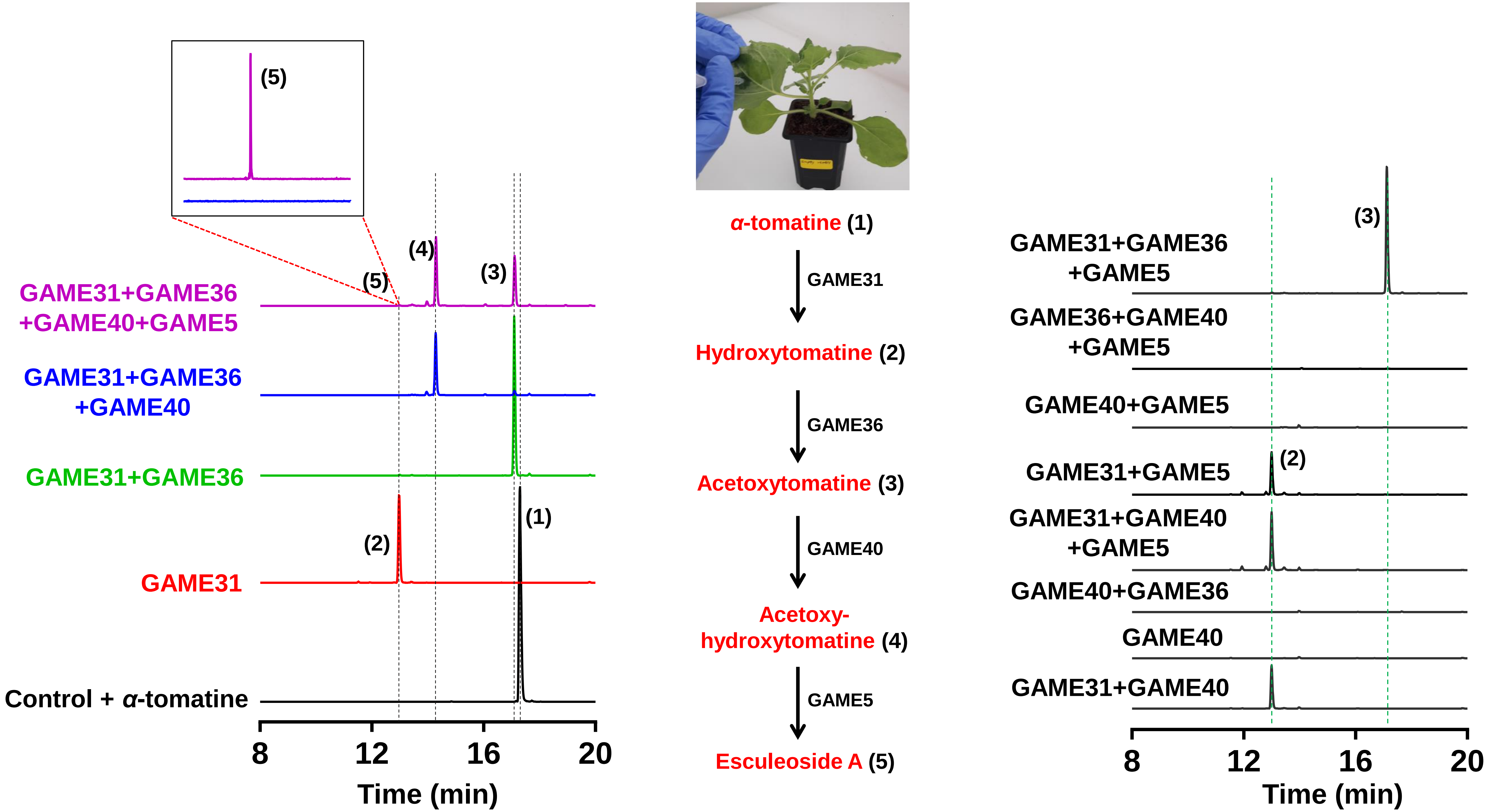
(5)
(4)
(3)
GAME31+GAME36
+GAME40+GAME5
GAME31+GAME36
+GAME40
GAME31+GAME36
(1)
(2)
GAME31
Control + α-tomatine
8
12
16
20
Time (min)
α-tomatine (1)
GAME31
Hydroxytomatine (2)
GAME36
Acetoxytomatine (3)
GAME40
Acetoxy-
hydroxytomatine (4)
GAME5
Esculeoside A (5)
(3)
GAME31+GAME36
+GAME5
GAME36+GAME40
+GAME5
GAME40+GAME5
(2)
GAME31+GAME5
GAME31+GAME40
+GAME5
GAME40+GAME36
GAME40
GAME31+GAME40
8
12
16
20
Time (min)

Supplementary Figure 15: Transient co-expression of four tomato *GAME* genes (SlGAME31, SlGAME36, SlGAME40 and SlGAME5) in *N. benthamiana* enables step-by-step conversion of co-infiltrated *α*-tomatine substrate into Esculeoside A. Esculeoside A pathway intermediates were observed upon the transient expression of corresponding *GAME* genes (left panel). Extracted ion chromatograms from LC-MS are shown. Various *GAME* genes combinations (right panel, shown in black) were included in the transient expression experiments to test the promiscuity of GAME enzymes on Esculeoside A pathway intermediates. Leaves co-infiltrated with empty vector and *α*-tomatine were used as control (shown in black, left panel). *m/z*: mass to charge.

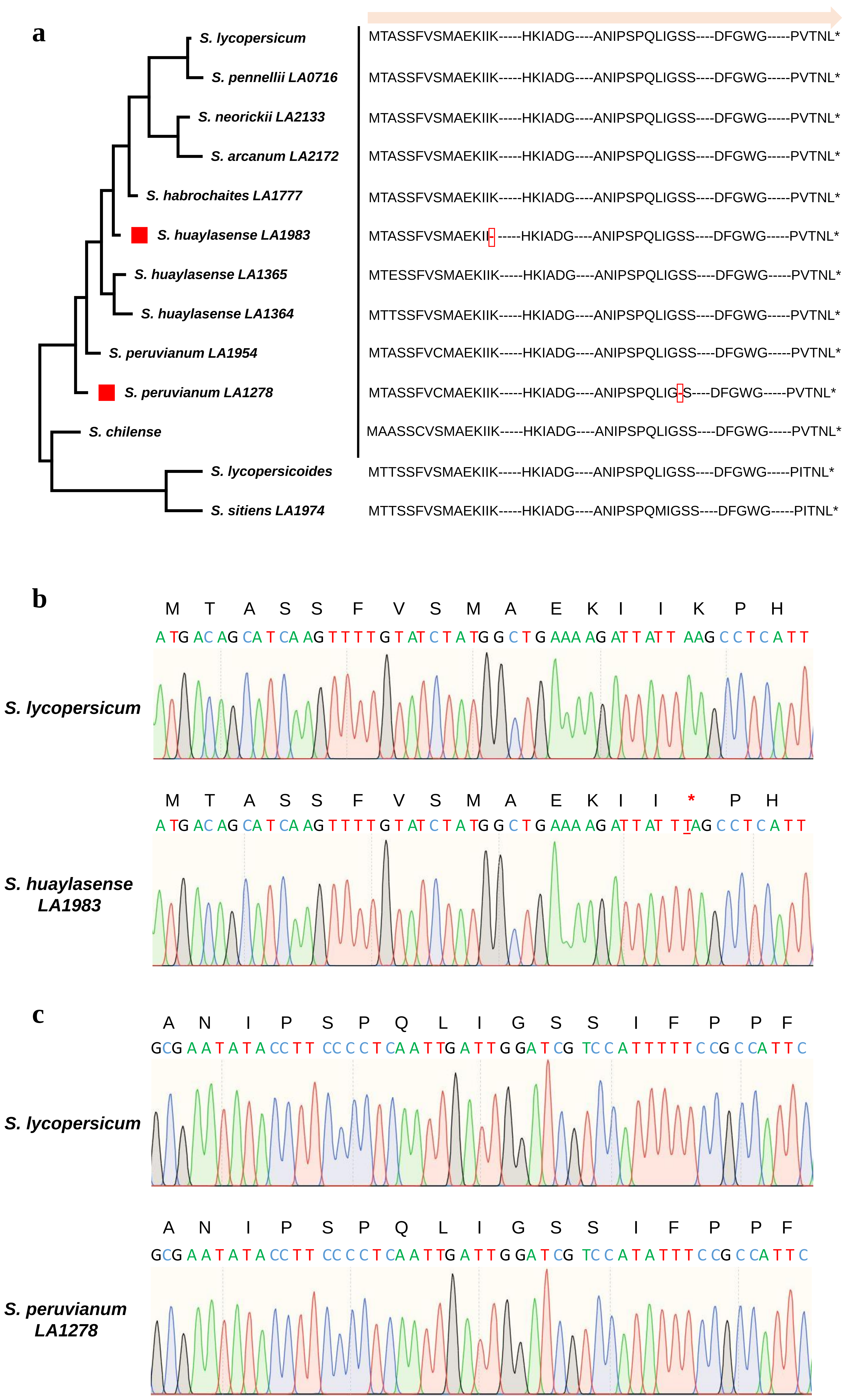

a
S. lycopersicum
S. pennellii LA0716
S. neorickii LA2133
S. arcanum LA2172
S. habrochaites LA1777
S. huaylasense LA1983
S. huaylasense LA1365
S. huaylasense LA1364
S. peruvianum LA1954
S. peruvianum LA1278
S. chilense
S. lycopersicoides
S. sitiens LA1974
MTASSFVSMAEKIIK-----HKIADG----ANIPSPQLIGSS----DFGWG-----PVTNL*
MTASSFVSMAEKIIK-----HKIADG----ANIPSPQLIGSS----DFGWG-----PVTNL*
MTASSFVSMAEKIIK-----HKIADG----ANIPSPQLIGSS----DFGWG-----PVTNL*
MTASSFVSMAEKIIK-----HKIADG----ANIPSPQLIGSS----DFGWG-----PVTNL*
MTASSFVSMAEKIIK-----HKIADG----ANIPSPQLIGSS----DFGWG-----PVTNL*
MTASSFVSMAEKII------HKIADG----ANIPSPQLIGSS----DFGWG-----PVTNL*
MTESSFVSMAEKIIK-----HKIADG----ANIPSPQLIGSS----DFGWG-----PVTNL*
MTTSSFVSMAEKIIK-----HKIADG----ANIPSPQLIGSS----DFGWG-----PVTNL*
MTASSFVCMAEKIIK-----HKIADG----ANIPSPQLIGSS----DFGWG-----PVTNL*
MTASSFVCMAEKIIK-----HKIADG----ANIPSPQLIG-S----DFGWG-----PVTNL*
MAASSCVSMAEKIIK-----HKIADG----ANIPSPQLIGSS----DFGWG-----PVTNL*
MTTSSFVSMAEKIIK-----HKIADG----ANIPSPQLIGSS----DFGWG-----PITNL*
MTTSSFVSMAEKIIK-----HKIADG----ANIPSPQMIGSS----DFGWG-----PITNL*
b
M T A S S F V S M A E K I I K P H
ATGACAGCATCAAGTTTTGTATCTATGGCTGAAAAGATTATT AAGCCTCATT
S. lycopersicum
M T A S S F V S M A E K I I * P H
ATGACAGCATCAAGTTTTGTATCTATGGCTGAAAAGATTAT TTAGCCTCATT
S. huaylasense LA1983
c
A N I P S P Q L I G S S I F P P F
GCGAATATACCTT CCCCTCAATTGATTGGATCG TCCATTTTTCCGCCATTC
S. lycopersicum
A N I P S P Q L I G S S I F P P F
GCGAATATACCTT CCCCTCAATTGATTGGATCG TCCATATTTCCGCCATTC
S. peruvianum LA1278

Supplementary Figure 16: GAME36 variant proteins in tomato clade of the genus *Solanum*. (a) GAME36 genotype variants from cultivated and wild tomato species were obtained from NCBI and other tomato public databases. Two wild tomato accessions, *S. huaylasense* LA1983 and *S. peruvianum* LA1278 were predicted to encode truncated GAME36 proteins due to introduction of premature stop codons in sequence (shown in red dash). As typically observed for BAHD acyltransferase family proteins, GAME36 protein sequences analyzed here also contains two conserved amino acid motifs HKIAD and DFGWG. **(b, c)** Sanger sequencing results of cloned *GAME36* sequences from *S. huaylasense* LA1983 (b) and *S. peruvianum* LA1278 (c) accessions. Among these two species, nonsense mutation was confirmed only in *S. huaylasense* LA1983, and not in *S. peruvianum* LA1278 as predicted (see panel a) Sequencing chromatograms from wild tomato accessions were compared to cultivated tomato (*S. lycopersicum cv.* Moneymaker) *GAME36* sequence.

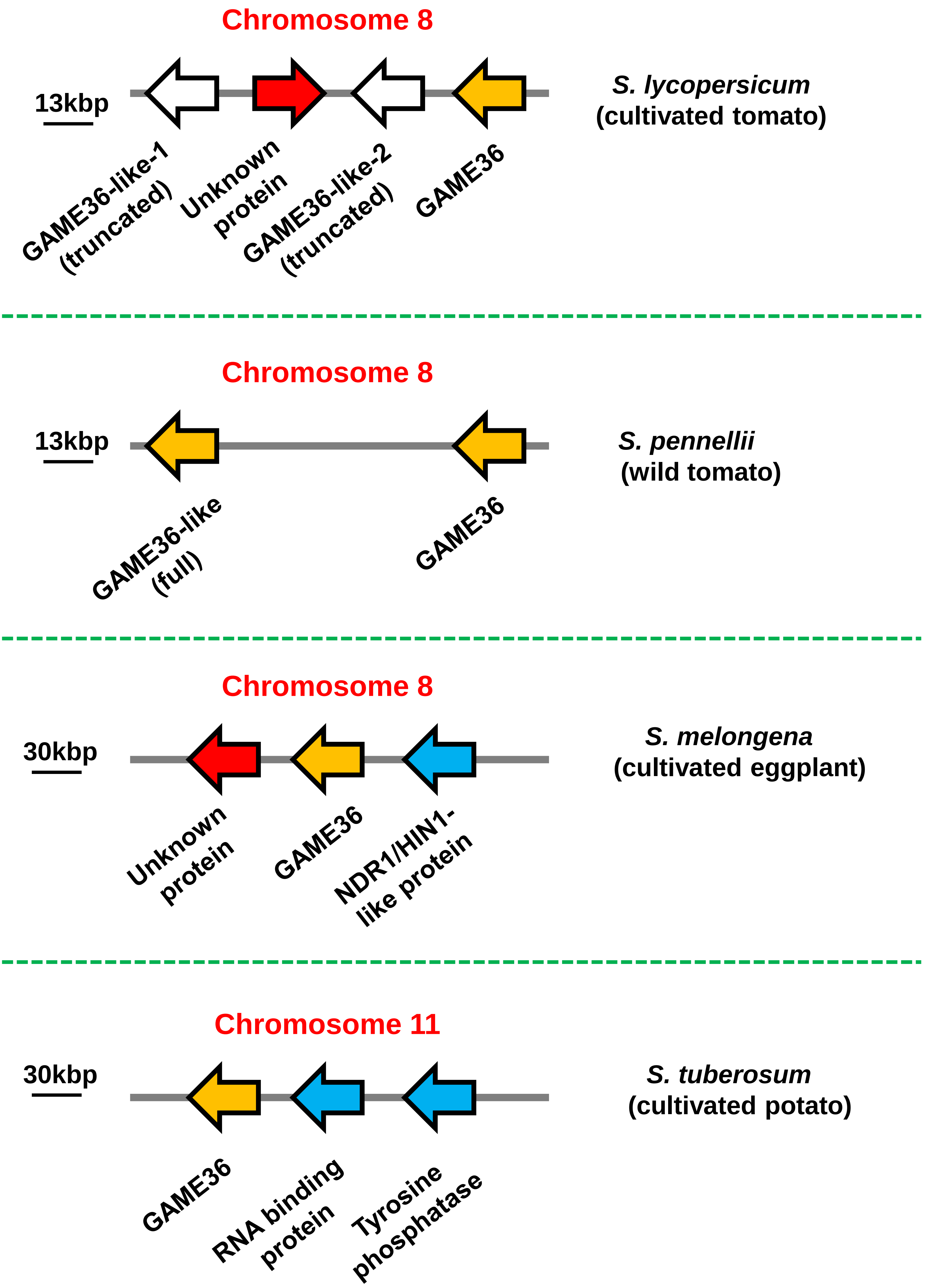
Chromosome 8
13kbp
S. lycopersicum
(cultivated tomato)
GAME36-like-1
(truncated)
Unknown
protein
GAME36-like-2
(truncated)
GAME36
Chromosome 8
13kbp
S. pennellii
(wild tomato)
GAME36-like
(full)
GAME36
Chromosome 8
30kbp
S. melongena
(cultivated eggplant)
Unknown
protein
GAME36
NDR1/HIN1-
like protein
Chromosome 11
30kbp
S. tuberosum
(cultivated potato)
GAME36
RNA binding
protein
Tyrosine
phosphatase

Supplementary Figure 17: Genomic organization of *GAME36* and *GAME36-like* genes associated with SGA metabolism in selected cultivated and wild *Solanum* species. *GAME36* and *GAME36-like* (full length) are shown in yellow block arrow. Truncated *GAME36-like* genes (white block arrows), unknown proteins (red block arrow) and other genes (blue block arrow) located next to *GAME36* on chromosomal region of respective *Solanum* species are presented. The arrowheads represent direction of transcription.

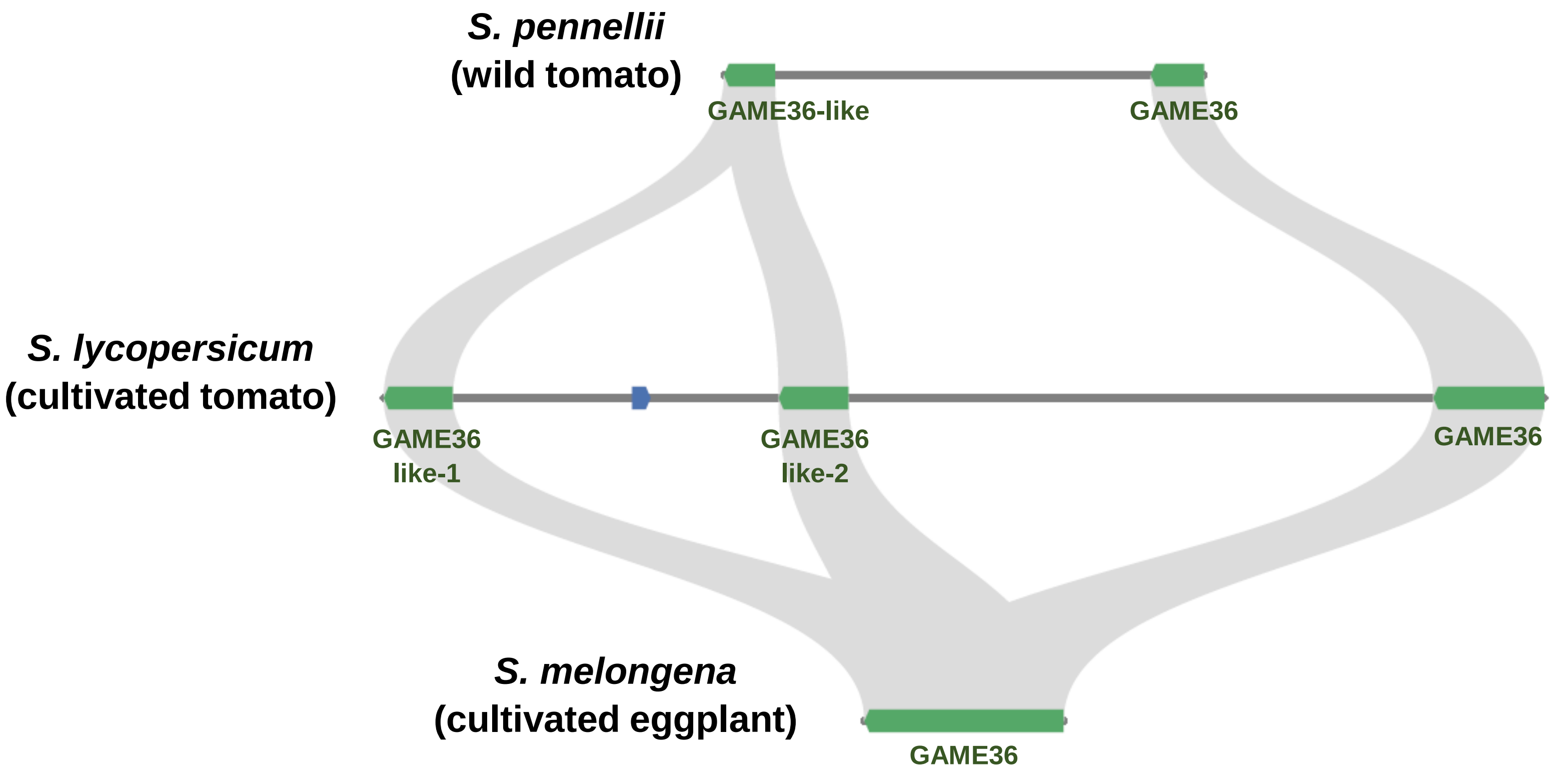

S. pennellii
(wild tomato)
GAME36-like
GAME36
S. lycopersicum
(cultivated tomato)
GAME36
like-1
GAME36
like-2
GAME36
S. melongena
(cultivated eggplant)
GAME36

Supplementary Figure 18: Syntenic alignment of GAME36 and GAME36-like genes between cultivated tomato, cultivated eggplant and wild *S. pennellii*.

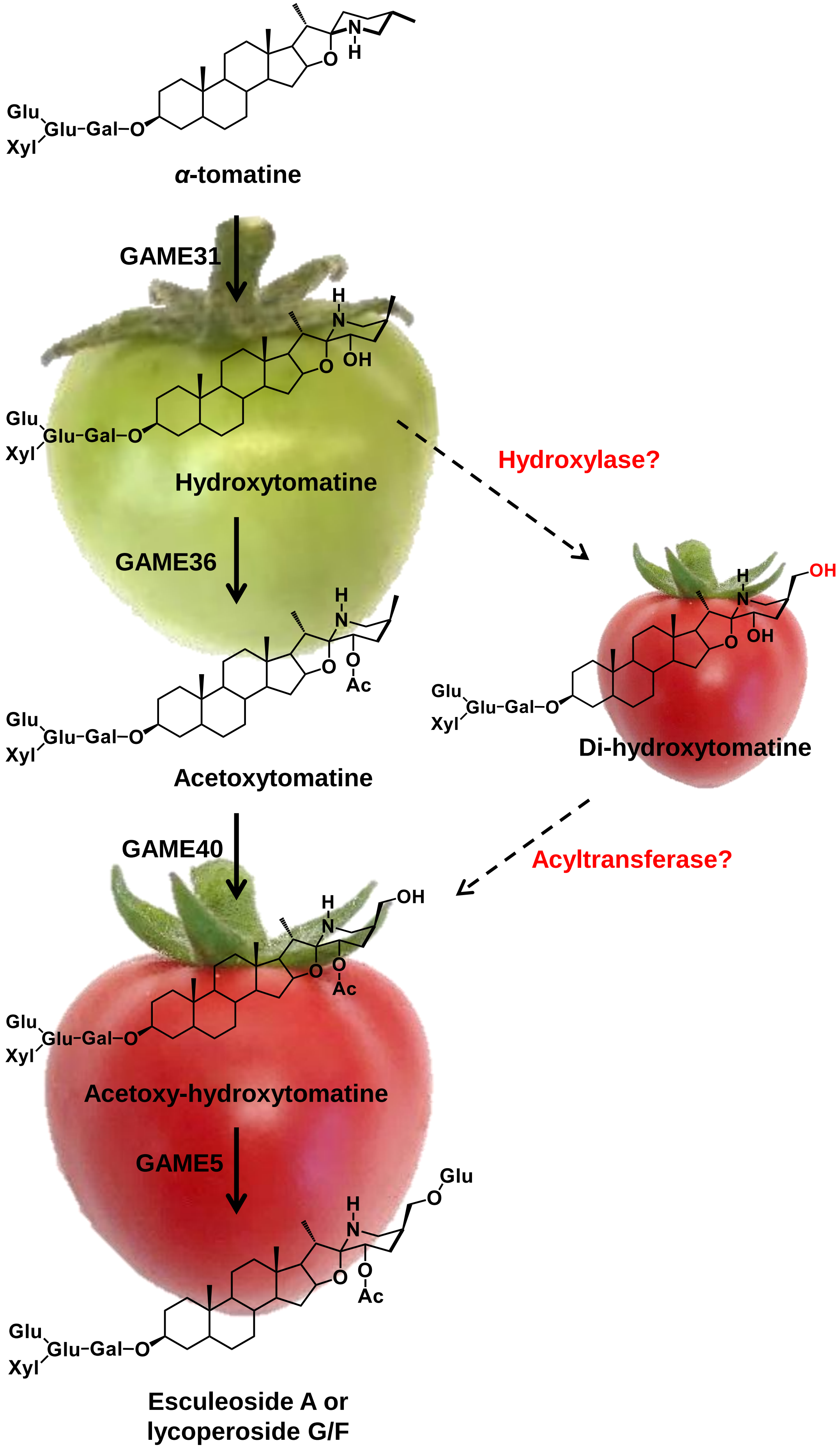

α-tomatine
GAME31
Hydroxytomatine
GAME36
Acetoxytomatine
GAME40
Acetoxy-hydroxytomatine
GAME5
Esculeoside A or
lycoperoside G/F
Hydroxylase?
Di-hydroxytomatine
Acyltransferase?
Glu
Glu-Gal-O
Xyl
OH
O
Ac

Supplementary Figure 19: Alternative minor biosynthetic route to Esculeoside A formation in tomato red ripe fruit. The major biosynthetic route for Esculeoside A production from α-tomatine involving four GAME enzymes is shown in bold black arrows. The alternative route involving di-hydroxytomatine as pathway intermediate is shown in dashed arrows, while proposed enzymes in this pathway are marked in red. GAME: GLYCOALKALOID METABOLISM; Glu: Glucose; Gal: Galactose; Xyl: Xylose; Rha: Rhamnose and Ac: Acetoxy.

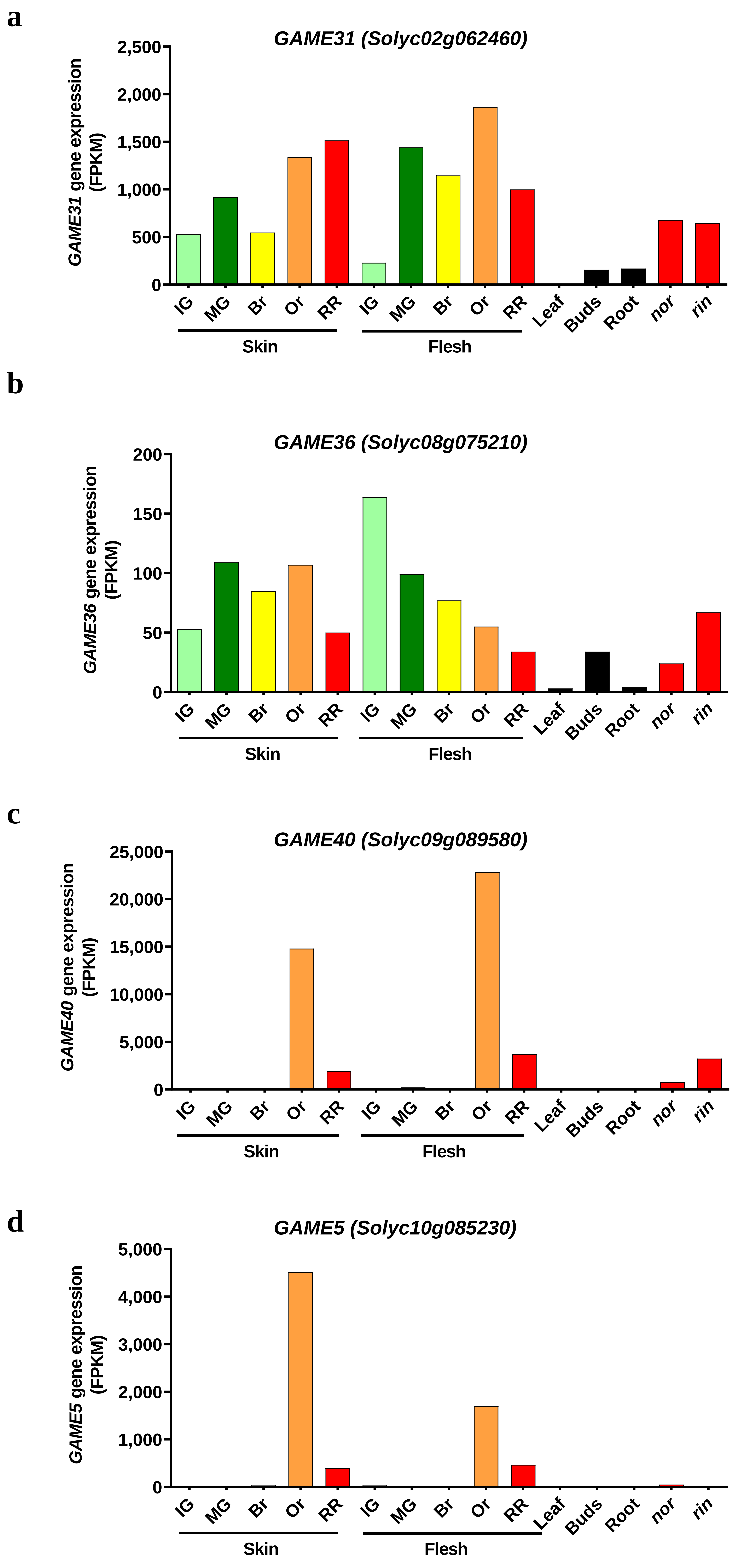
a
GAME31 (Solyc02g062460)
GAME31 gene expression (FPKM)
2,500
2,000
1,500
1,000
500
0
IG MG Br Or RR IG MG Br Or RR Leaf Buds Root nor rin
Skin
Flesh
b
GAME36 (Solyc08g075210)
GAME36 gene expression (FPKM)
200
150
100
50
0
IG MG Br Or RR IG MG Br Or RR Leaf Buds Root nor rin
Skin
Flesh
c
GAME40 (Solyc09g089580)
GAME40 gene expression (FPKM)
25,000
20,000
15,000
10,000
5,000
0
IG MG Br Or RR IG MG Br Or RR Leaf Buds Root nor rin
Skin
Flesh
d
GAME5 (Solyc10g085230)
GAME5 gene expression (FPKM)
5,000
4,000
3,000
2,000
1,000
0
IG MG Br Or RR IG MG Br Or RR Leaf Buds Root nor rin
Skin
Flesh

Supplementary Figure 20: Expression of *GAME genes* involved in Esculeoside A pathway. (a-d) Normalized *GAME31* (a), *GAME36* (b), *GAME40* (c), *GAME5* (d) expression level in tomato (*cv.* Micro Tom) tissue types, fruit developmental stages and ripening impaired mutants (RNA-seq expression data). Fruit developmental stages- IG: immature green; MG: mature green; Br: breaker; Or: orange; RR: red ripe. FPKM: Fragments Per Kilobase of transcript per Million mapped reads.
