# Supplementary material for: A BAHD-type acyltransferase concludes the biosynthetic pathway of non-bitter glycoalkaloids in ripe tomato fruit
Source: Nat Commun. 2023 Jul 27;14:4540. doi: 10.1038/s41467-023-40092-5 (PMC10374582; doi:10.1038/s41467-023-40092-5)
Supplement: Supplementary file 2 — Description of Additional Supplementary Files [file 41467_2023_40092_MOESM2_ESM.pdf]

Description of Additional Supplementary Files

File name: **Supplementary Data 1**

Description: BAHD acyltransferases in tomato (*S. lycopersicum*) genome. Previously characterized BAHDs are shown in green color. The newly characterized GAME36 BAHD acyltransferase in this study is marked in red.

File name: **Supplementary Data 2**

Description: GAME36 sequences from cultivated and wild tomato accessions used in the construction of phylogenetic tree (in Figure 7a). All BAHD sequences from tomato including GAME36, GAME36 and homologous (GAME36-like) sequences from wild and cultivated tomato species as well as from potato and eggplant, used in the construction of phylogenetic tree (Figure 8).

File name: **Supplementary Data 3**

Description: Putative steroidal glycoalkaloids identified in recombinant enzyme assays.

File name: **Supplementary Data 4**

Description: Oligonucleotides used in this study
