# Supplementary material for: A BAHD-type acyltransferase concludes the biosynthetic pathway of non-bitter glycoalkaloids in ripe tomato fruit
Source: Nat Commun. 2023 Jul 27;14:4540. doi: 10.1038/s41467-023-40092-5 (PMC10374582; doi:10.1038/s41467-023-40092-5)
Supplement: Supplementary file 4 — Supplementary Data 2 [file 41467_2023_40092_MOESM4_ESM.pdf]

Supplementary Data 2

GAME36 sequences from cultivated and wild tomato accessions used in the construction of phylogenetic tree (Figure 7a)

> S. lycopersicum
MTASSFVSMAEKIIKPHSPTPFSVKRYNLCLMDEIMVPVYMPIVAFYPNPSKTPEQVSNI
LEDSLSKVLSSYYPFAGTLGSDNATFVDCNDRGAKSIQVRYDCPMSEIVNLPDTGPEY
LPFAKGTPWSSTPEEQSLLVVQLSHFNCGGLGISARLSHKIADGCTLANFISDWASVAR
DDNANIPSPQLIGSSIFPPFTEMRIHTDTNVDYEFYNLPVCKKRYLFSNAKLEMLKTQVE
SETGVQNPTRIEVLSALIYKCAVTANSSSFRPSSLSLPVNLRPILNPPLETRTVGNIISFIK
VETTSEDEMTIGRVVREIRKGKDELKQEGGVKKEKLVSLWSEWIHSIDLYRSSSVCNY
PLNNLDFGWGKPNRVAIPVFGVANTCMFMDNLSGDGIEVIIALPEKDATQFENSKELLH
FASPVTNL

> S. pennellii LA0716
MTASSFVSMAEKIIKPHSPTPFSLKRYNLCLTDEIMVPVYMPIVAFYPNPSKTPEQVSNI
LEDSLSKVLSSYYPFAGTLGSDNATFVDCNDRGAKSIQVRYDCPMSEIVNLPDTGPEY
LPFAKGTPWSSTPEEQSLLVVQLSHFNCGGLGISARLSHKIADGCTLANFISDWASVAR
DDNANIPSPQLIGSSIFPPFTEMRIHTDTNVDYEFYNLPVCKKRYLFSNAKLEMLKTQVE
SETGVQNPTRIEVLSALIYKCAVTANSSSFRPSSLSLPVNLRPILNPPLETRTVGNIISFIK
VETTSEDEMTIGRVVREIRKGKDELKQEGGVKKEKLVSLWSEWIHSIDLYRSSSVCNY
PLNNLDFGWGKPNRVAIPVFGVANTCMFMDNLSGDGIEVIIALPEKDATQFENSRELLH
FASPVTNL

> S. chilense
MAASSCVSMAEKIIKPHSPTPSSLKRYNLCLTDEIMVPVYMPIVALYPFSSKTPQQVSNI
LENSLSKLLSSYYPFAGTLGPDNATFVDCNDRGAKFIQVRYDCTMSEIVNLPDTGPEYL
PFAKGTPWSSTPDEQSLLLVQLSHFNCGGLAISVRLSHKIADGCTLANFISDWASIARD
DNANIPSPQLIGSSIFPPFTETPSTGIHTDTNVDYEFYNLPVSKKRYLFSNSKLEMLKTQ
VESETGVQNPTRIEVLSALIYKCAVTAARANSSSYKPSSLSLPVNLRPILNPPLETRTIGN
IISFIKVDTMSEDEMTIGRVVREIRKGKDELKQEGDVKKEKLVSLWNEWIHSIELYRSSS
VCNYPLNNLDFGWGKPNRVTIPVFGVANTCMFMDNLSGDGIEVIIVLPEKDVTQFENS
KELLQFASPVTNL

> S. lycopersicoides
MTTSSFVSMAEKIIKPHSPTPFSLRRYNLCLTDEIMVPVYMPIVTFYPFSSKTPQQVSNI
LENSLSKLLSSYYPFAGTLGDNNTFVDCNDRGAKFIQVRYDCPMSEIVNLPDTGPEYLP
FAKGTPWSSTPDEQSLLLVQLSHFNCGGLAISVRLSHKIADGCTLCNFISDWASIARDD
NANIPSPQLIGSSIFQPSTEMPSTGIHMDTNVDYEFYNLPFSKKRYLFSNSKLEMLKSEV
TSETGVQNPTRIEVLSALIYKCAATAARANSSSFKPSSLSLPVNLRPILNPPLETRTIGNI
LSFIKVDTTSEDEMTIGRVVCEIRKGKDELKKEEWHVKKEKLVSLWSEWIYSIELYRSSS

VCNYPLNNLDFGWGKPNRVTIPVFGVANTCMFMDNLSGDGIEVIVVLPEIDVTQFENS
KELLDFASPITNL

> S. sitiens LA1974
MTTSSFVSMAEKIIKPHSPTPFSLRRYNLCLTDEIMVPVYMPIVTFYPFSSKTPPQLSNIL
ENSLSKLLSSYYPFAGTLGDNNTFVDCNDRGAKFIQVRYDCPMSDIVNLPDTGPEYLP
FAKGTPWSSTPDEQSLLLVQLSHFNCGGLAISVRLSHKIADGCTLCNFISDWASIARDD
NANIPSPQMIGSSIFPPSTEMPSTGIHMDTNVDYEFYNLPVSKKRYLFSNSKLEMLKSK
VESETGVQNPTRIEVLSALIYKCAATAARANSSSFKPSSLSLPVNLRPVLNPPLETRTIG
NILSFIKVYTMSEDEMTIGRVVCEIRKGKDELKKEEWHVKKEKLVSLWSEWIYSIELYRS
SSVCNYPLNNLDFGWGKPNRVTIPVFGVANTCMFMDNLSGDGIEVIIVLPEKDVTQFE
NSKELLEFASPITNL

> S. huaylasense LA1983
MTASSFVSMAEKII-
PHSPTPFSVKRYNLCLMDEIMVPVYMPIVAFYPNPSKTPEQVSNILEDSLSKLLSSYYPF
AGTLGPDNATFVDCNDRGAKFIQVRYDCTMSEIVNLPDTGPEYLPFAKGTPWSSTPDE
QSLLLVQLSHFNCGGLGISVRLSHKIADGCTLANFISDWASIARDDNANIPSPQLIGSSIF
PPFTEMRIHTDTNVDYEFYNLPVCKKRYLFSNAKLEMLKTQVESETGVQNPTRIEVLSA
LIYKCAVTLDEANSSSFKPSSLSLPVNLRPILNPPLETRTIGNIISFIKVETTSEDEMTIGR
VVREIRKGKDELKQEGDVKKEKLVSLWSEWIHSIELYRSSSVCNYPLNNLDFGWGKPN
RVAIPVFGVANTCMFMDNLSGDGIEVIIVLPEKDVTQFENSKELLQFASPVTNL

> S. neorickii LA2133
MTASSFVSMAEKIIKPHSPTPFSVKRYNLCLMDEIMVPVYMAIVAFYPNPSKTPQQVSNI
LEDSLSKLLSSYYPFAGTLGPDNATFVDCNDRGAKFIQVRYDCPMSEIVNLPDTGPEYL
PFAKGTPWSSTPEEQSLLVVQLSHFNCGGLGISVRLSHKIADGCTLANFISDWASVAR
DDNANIPSPQLIGSSIFPPFAEMRIHTDTNVDYEFYNLPVCKKRYLFSNAKLEMLKTQVE
SETGVQNPTRIEVLSALIYKCAVTLDEANSSSFKPSSLSLPVNLRPILNPPLETRTVGNII
SFIKVDTTSEDEMTIGRVVREIRKGKDELKQEGDVKKEKLVSLWSEWIHSIELYRSSSV
CNYPLNNLDFGWGKPNRVAIPVFGVANICMFMDSLSGDGIEVIIVLPEKDVTQFENSEE
LLHFASPVTNL

> S. huaylasense LA1365
MTESSFVSMAEKIIKPHSPTPFSVKRYNLCLMDEIMVPVYMPIVAFYPNPSKTPQQVSNI
LEDSLSKLLSSYYPFAGTLGSDNATFVDCNDRGAKFIQVRYDCTMSEIVNLPDTGPEYL
PFAKGTPWSSTPDEQSLLLVQLSHFNCGGLAISARLSHKIADGCTLANFISDWASIARD
DNANIPSPQLIGSSIFPPFTEMRIHTDTNVDYEFYNLPVCKKRYLFSNAKLEMLKTQVES
ETGVQNPTRIEVLSALIYKCAVTLDEANSSSFTPSSLSLPVNLRPILNPPLETRTIGNIISFI
KVETTSEDEMTIGRVAREIRKGKDELKQEGDVKKEKLVSLWNEWIHSIELYRSSSVCNY
PLNNLDFGWGKPNRVAIPVFGVANTCMFMDNLSGDGIEVIIVLPEKDVTQFENSKELLQ
FASPVTNL

> S. huaylasense LA1364
MTTSSFVSMAEKIIKPHSPTPFSVKRYNLCLMDEIMVPVYMPIVAFYPNPSKTPQQVSNI
LEDSLSKLLSSYYPFAGTLGSDNATFVDCNDRGAKFIQVRYDCTMSEIVNLPDTGPEYL
PFAKGTPWSSTPEEQSLLVVQLSHFNCGGLAISARLSHKIADGCTLANFISDWASIARD
DNANIPSPQLIGSSIFPPFTEMRIHTDTNVDYEFYNLPVCKKRYLFSNAKLEMLKTQVES
ETGVQNPTRIEVLSALIYKCAVTLDEANSSSFRPSSLSLPVNLRPILNPPLETRTIGNIISFI
KVETTSEDEMTIGRVVREIRKGKDELKQEGDVKKEKLVSLWNEWIHSIELYRSSSVCNY
PLNNLDFGWGKPNRVAIPVFGVANTCMFMDNLSGDGIEVIIVLPEKDVTQFENSKELLQ
FASPVTNL

> S. peruvianum LA1278
MTASSFVCMAEKIIKPHSPTPFSVKRYNLCLMDEIMVPVYMPIVALYPNPSKTPQQVSNI
LEDSLSKLLSSYYPFAGTLVSDNATFVDCNDRGAKFIQVRYDCTMSEIVNLPDTGPEYL
PFAKGTPWSSTPDEQSLLLVQLSHFNCGGLAISVRLSHKIADGCTLANFISDWASIARD
DNANIPSPQLIG-
SIFPPFTEMRIHTDTNVDYEFYNLPVSKKRYLFSNAKLEMLKTQVESETGVQNPTRIEVL
SALIYKCAVTANSSSFKPSSLSLPVNLRPILNPPLETRTIGNIISFIKVETMSEDEMTIGRV
VREIRKGKDELKQEGDVKKEKLVSLWNEWIHSIELYRSSSVCSYPLNNLDFGWGKPNR
VTIPVFGVANTCMFMDNLSGDGIEVIIVLPEKDVTQFENSKELLQFASPVTNL

> S. peruvianum LA1954
MTASSFVCMAEKIIKPHSPTPFSVKRYNLCLMDEIMVPVYMPIVAFYPNPSKTPEQVSNI
LEDSLSKLLSSYYPFAGTLGSDNATFVDCNDRGAKFIQVRYDCTMSEIVNLPDTGPEYL
PFAKGTPWSSTPEEQSLLLVQLSHFNCGGLAISVRLSHKIADGCTLANFISDWASIARD
DNANIPSPQLIGSSIFPPFTEMRIHTDTNVDYEFYNLPVCKKRYLFSNAKLEMLKTQVES
ETGVQNPTRIEVLSALIYKCAVTLDEANSSSFKPSSLSLPVNLRPILNPPLETRTIGNIISFI
KVETMSEDEMTIGRVVREIRKGKDELKQEGGVKKEKLVSLWNEWIHSIELYRSSSVCN
YPLNNLDFGWGKPNRVTIPVFGVANTCMFMDNLSGDGIEVIIVLPEKDVTQFENSKELL
QFASPVTNL

> S. arcanum LA2172
MTASSFVSMAEKIIKPHSPTPFSVKRYNLCLMDEIMVPVYMAIVAFYPNPSKTPQQVSNI
LEDSLSKLLSSYYPFAGTLGPDNATFVDCNDRGAKFIQVRYDCPMSEIVNLPDTGPEYL
PFAKGTPWSSTPEEQSLLVVQLSHFNCGGLGISVRLSHKIADGCTLANFISDWASIARD
DNANIPSPQLIGSSIFPPFTEMPIHTDADVDYEFYNLPVCKKRYLFSNAKLEMLKTQVES
ETGVQNPTRIEVLSALIYKCAVTLDEANSSSFKPSSLSLPVNLRPILNPPLETRTVGNIIS
FIKVDTTSEDEMTIGRVVREIRKGKDELKQEGDVKKEKLVSLWSEWIHSIDLYRSSSVC
NYPLNNLDFGWGKPNRVAIPVFGVANTCMFMDSLSGDGIEVIIVLPEKDVTQFENSEEL
LHFASPVTNL

> S. habrochaites LA1777
MTASSFVSMAEKIIKPHSPTPFSVKRYNLCLMDEIMVPVYMPIVAFYPNPSKTPEQVSNI
LEDSLSKLLSSYYPFAGTLGSDNATFVDCNDRGAKFIQVRYDCPMSEIVNLPDTGPEYL
PFAKGTPWSSTPDEQSLLLVQLSHFNCGGLGISARLSHKIADGCTLANFISDWASVAR
DDNANIPSPQLIGSSIFPPFTEMRIHTDTNVDYEFYNLPVCKKRYLFSNAKLEMLKTQVE

SETGVQNPTRIEVLSALIYKCAVTLDEANSSSFKPSSLSLPVNLRPILNPPLETRTIGNIIS
FIKVDTTSEDEMTIGRVVREIRKGKDELKQEGDVKKEKLVSLWSEWIHSIELYRSSSVC
NYPLNNLDFGWGKPNRVTIPVFGVANTCMFMDNLSGDGIEVIIVLPEKDVTQFENSKEL
LQFASPVTNL

All BAHD sequences from tomato including GAME36, GAME36 and homologous (GAME36-like) sequences from wild and cultivated tomato species as well as from potato and eggplant, used in the construction of phylogenetic tree (Figure 8)

>Solyc00g040290
MNLEFNSIVKILKSSLAETLVSYYAFSGDKVENSLKEVNFYNPDESIEGKFVPKKKHGVL
AIQMQVTQLNCGGTIVGCTFDHRVADAYSANLFLVSWSELAQSKPLSQLPSFRRSSLF
PRHPGYYDDSIDDLYVLLSTLPPMKSEIFYPTDQFISRIYYVTGDKIEYLQSLANCHDNQ
KGTSQRSKLESFSAFLWKTIACGINKETWGFNNFRFGIVVDGRTRLIINNVDKSLKGYF
DWVEAPRPEPSMAKIYAMNGDGPAVIVSSRQHFPAKKLNFGWGEPVFWSYHFPWAG
KAGYVMPMPIHKGNGDWIVYMHLLKCQIELIETSPYHVFKPVTAYYLNLI

>Solyc00g040390
MNLEFNSIVKILKSSLAETLVSYYALSGDKVENSLKEVNFYNPDESIEGKFVPKKKHGVL
AIQVTQLNCGGTIVGCTFDHRVADAYSANLFLVSWSELAQSKPLSQLPSFRRSSLFPR
HPGYYDDSIDDLYVLLSTLPPMKSEIFNPTDQFISRIYYVTGDKIEYLQSLANCHDNQKG
TSQRSKLESFSAFLWKTIACGINKETWGFNNFRFGIVVDGRTRLIINNVDKSLKGYFGN
VLSIPFGEKKVEEVKEKSLNWVAKVIHEFLDIAKTQEHFLGLIDWVEAPRPEPSMAKIYA
MNGDGPAVVVSSRQHFPAKKLNFGWGEPVFWSYHFPWAGKAGYVMPMPSHKGNG
DWIVYMHLLKWQIELIEASPYHVFKPVTAYYLNLI

>Solyc00g134620
MASFYFSTSFMLFSPCLLHLPFAYYAFSGDIVENSPGEPEILCNNGSVSFIEAFNDVELK
EINFYNPDESIEGKFVPKKKHGVLAIQSELAQSKPLSQLPSFRRSSLFPRHPGYYDDSID
DLYVPLSTLPPMKSEILNPNDQVISRIYYVTGDKIEHLQSLANYHDDQKGTSQRSKLESF
NWVEAHRPEPSMAKIYAMNGDGPAVVVSSGQHLPVKKINFEWGESAFWAYHFPWAG
KAGYVMPMLSPKGNGDWIVYMHLLKWQIELIEASPYHVFKPVTANYFNLI

>Solyc00g135260
MYIVENSAGEPEILCNNGGVSFIEAFGDVELKEINFYNPDESIEGKFVPKKKHGVLAIQM
KSEILNPNDQVISRIYYVTGDKIEHLQSLANYHDDQKGTSQRSKLESFSAFLWITIACGIN
KETWVSTILGLVLWSMVEIINNVDKSLKGYFGNVLSIPFGEKKVEEHFLGLIDWVEAHR
PEPSMAKIYAMNGDGPAVVVSSGQHLPVKKINFEWGESAFWAYHFPWAGKAGYVMP
MLSPKGNGDWIVYMHLLKWQIELIEASPYLMFKPVTANYFNLI

>Solyc01g005900
MTATEIPCSSQHLLENEEKPTTPNPTPTKNSKKLALVPLIFIIFFEVSGGPYGAEAAIGAA
GPLLAILGFLIFPFIWSVPEALVTAELATTFPGNGGVKASKIPSEEKVKFAYATNIRRIIIPK
LPFGYWGNGCVPMYVQFLAQELVNQPLSKTADSIKKSKFNTTDEYVRSFIDFQELHYH
EGITAGNRVSGFTDWRHLGHASVDFGWGGPMNVLPLSRHLVGSIEPCFFLPNSSTNE
GEKNGFKVLVYLQEEAMVDFKKEMNKLEHIGLSLL

>Solyc01g008300
MEKKVEITSQNLIKPKVCHIESFNFSALDQLAPLPHYPIFLYYPNDDQESTNISTKSQQL
KNSLSKILSDFYPFAGRLINENTSLSFNNHNNDDFGVLFIEAFAHNYNLQEDILLSGIKTN
TCGHFLPTLDSLLQTHLLIVQVTFFKCGGMILGCWVSHKLSDATSICSLINNWASTARE
GAVDAQLLLTPDFKTGVKIFPPTQKPYPSPFTNFVFNEQQLVSKIFLFNGPSIANLKTKA
LSKDVPSPTRVEAVSALIWKCATVSSKSSSVWKE

>Solyc01g068140
MVMDFLVTTKGSGGGNFITPLGQTPTGSVLDLSVIDSLAVLRCNARTLHVFKGGNSTVI
REAFGKALVPYYPLAGRLKFSAHNNQLLQIDCSGQGIWFVEASADCTLLDVNYFDEAS
ALDDTTFDKLLPQLTPLPVSNDSFSDPPLVLVQVTEFKCGGYVMGLTFCHSICDGLGA
AQFLKAVGEFARGVEKLSVAPVWCRELLLPLPQKGVAEDNSSMPPPPLTIPVPDQRLE
HASFDIPLDEINQLKHEVIQELNMDQNVFCSSFEIIAATLWRHRTRAILNITDDKKTSSNT
SFIPADDAGDEEVKLVFFANCRQLVPLPEGFYGNCFFPVTVTASNKVVAEASLAEVVKL
IKDAKADLPAEFSQWLNNKTNKPTNDDQFNDPFAPGQGVGYNTLFVSEWGRLGFNEV
DYGWGKPVHVIPVQGSAVIPVGIVSKQPLPKTGIRLMTWCVHSHHLHTFLNMMML

>Solyc01g105550
MACRLDIEIQSRKLLKPSASTPDNLRRLKLSLFDQLALRTYIPVLFNYLPSSSSTSYDDE
LEKSLAETLTKFYPFAGRFAKDIDPFSIDCNDEGVEYVQTKVNADDLAQFLRGQAHND
SESSLIDLLPIKDVEPSSPSSPLFGVQVNVFNNGGVTIGIQISHIVADAFTMATFVNEWA
HTCLTGRTVSNNPGFGQLSLLFPAKVLQFPSPSPDLNTNTTTTGPNYKIVTRRFVFDAL
AIENLRKTIKDNDMMMKQPSRVVVIMSLMWKVLTHISSAKNNGNSRDSSLGFPINMRG
KLSCTAPSLEHALGNYGMMGIADRKARRKDDELNDFVKLVGNTIWNTCEAIGKAESVD
DISSLAFNNHIKGVEKLLQEDKMDVYGTTSWCKLPWYEADFGWGKPFWVSPVGLNLI
EGAILMDTKDGNGVQLTICLKEKNMTEFEKHLHIFSSTPILG

>Solyc01g105590
MAKLDIEIQTRKILKPSAPTPDNLRRLKISLFDQLARSAYVSIVFNYLPSSSSSYDDDKLE
KSLAETLTKFYPFAGRLAKDDPFSIDCNDEGVEYVRTKVNADDLAQFLGKDDDDIESSL
IDLLPIKDVELSSPSSPLFGVQVNVFNNGGVSIGIQISHFLADAFTLATFVNEWAHTNTLS
SMPQDNNDLHKFGDLSSLFPPKMLQLPSFDPNTSSTTTTVPSYKNVTKRFVFDASAIE
SLKKTIKDDSSMMRKPTRLVVVMSLLWKVLARISSAKNGNSRDSCFGFVISFRGKVSCI
PSTEHVLGTFSIPEIANMEGDVARKDELNGFVKLVGNRIGETFAAIDKASKVDDIYSLTL
NNQIKVIEKFVQRDKMDFYGTTSWCKLPWYETDFGWGKPFWVTPVSFRIYEQTTLMD
TKDGDGIEIIVTMKENDMTEFERDPHILSSTSKLTFG

>Solyc01g107050
MASKVSIISTCTVKSCGRSQHHPDNCEIIELTPWDIYELQIEYFQNGLLFLMPTFEQVRD
VSKSTNTTSLIDHLRVSLSRTLDFFPPFCGRLEAAQAQEEKSGTTGFFVNCNNVGVQF
NHAIADGVTVGDIMESNLVPHVVHDFFPLSGVQNTEATSQPFLAVQVTELVDGVFIGCS
ANHALVDGSSYWHFYNSWAEISRGCNVISKIPYLKRKFPFKMDHFSDRIYIPNERINAG
DKFTPPALREKAFHFSRENVSKLKAKANHQMNTTKISSLQAVLAHLWQSVIRCRRLDH
SEETTFEVSIDMRKRLNPPLPEGFFGNAIYPAPTTIKTGELLKHGFGWAALQINKTIATH
DHEKLKCIYENWMKEPEVPKLGDLPSNYFMLNGSPQFNVYKYDFGWGKPIAHRGGV
GNLLDGKITVAPGLEEGSMIVEICLSSETIQALEEDIIFGEFVNNTTPMVGVERTIRSRI

>Solyc01g107070
MLRERVFHFTKESVAKLKAKANLVMNTTKISSLQAVLAHVWRSVIRCRHLDLSEETTFE
VSIDMRGRLNPPLPEGFFGNAICPATVTIKTGELLEHEFGWAALQINETIALHDQEKLKC
IYESWMNDPEYDFGWGKPITHRSGIGNMLEGKITVSPGQEEGSMILEICRL

>Solyc01g107080
MASKVSIISTWTVKAAGTSQHHTDNCEIIELTPWDILALQIDYAQSGLLYPMPTGEQVRD
VSKSTNTTTLIDHLRVSLSRALDFYPPFCGRLEAITEQSGTTSFVINCNNAGVLFSHAIA
GGVTIRDIIESNCVPHVVRDFFSLNGVQNQEATSQLPCLAVQVTELVDGIFIGCTNNHV
VVDGTSFWNFYNSWAELSRGSKIISKIPFLKREFPFKIDGFYPNRISIPNERINSGDKFIP
PALQMLREKVFHFTKENIYKLKAKANHEMNTTKISSLQAVLAHVWRSVIRCRRLDHNEE
TTFEVSINMREKVNPPLPEGYFGNAIYPVTVTIKTGQLLEHGFGWAALQINESIASHDHE
KLKCIYENWMKDPEIQKLDELPSNYFMLHNSPRFNFFKYDFGWGKPIAHRSGEGNML
DGKITVSPGVEEGSMIVEICLSSETIQALEEDIIFGEFVNTTPMVAVEPTIRARI

>Solyc02g062710
MASKVSIMSTWTVKAAGRSVIDDCEIIELTPWDILELQIEYGQGGVLCYMPTSQQMKDI
TKATNTTSLMDHLRVSLSRVLDFFPPLCGRLEAEEEQSGGFFIKCNDAGVQFNHAVAD
GVTVDDIMDQSKCVPHVVRDFFPLNGVRNIEATSKPFLGVQVTELVDGIFIGCTANHSL
LDGSSFWHFFTSWAEISRGFNVISQIPFLKRQFPFEIDNFSNRICIPNERINSNSDSTHE
HDPPALQDKIFHFTKESIAKLKSKANLEMKTTKISSLQAVLAHVWQSIIRCRNLDHNEET
TFEVPMDMRKRLNPPLPEGFFGNAIYPATITVKAGDLLKDGGFEWAALQINEMIASHDH
EKFKRIYENWMKDPEITKLGDLPRNYFMLHNSPRFNYNKYDFGWGKPIAQRGGMSNM
LEGKIDVSPGIEEGSMIFEICLSPKTIQALEEHKMFVAME

>Solyc02g079490
MGAKKDFNVKLIKTEVVAAMLPMQEHWLSQSNLDLLLPPVDVGVFLCYQNPIISGILSK
NWSFDSMVNVLKVSLGETLVSYYAFAGELIQNLAGEPEILCNNAGVDFIESWADVELKE
INFHNPDESIEGKLVPKKKHGVLAVQVTELKCGGVVVGCTFDHRVADAYSFNMFLVSW
AELAQSKPLSQLPSFRRSFLTPRCPSFYDPIIDTMYLPISALKQETANIDHDADEIISRIYY
VKAEEIRRLQSLANCNNTTFTKLEAFSAFLWKTIASDTNNKFRLGIVVNGRSRLSNGDE
EQAKILEGYFGNVLSIPFGEKKVEELKEKSLSWVASAVHEFLEHVVTREHFLGLINWVE

NHRPEPALARIYATNEDTPALVVSSGQQFPVRKIEFGWGEAVFGSYHFPWEGKSGYV
MPMPSPKGNGDWIVYMHMLKGQIDLIEAIASNVFKPLTADYLSLK

>Solyc02g081740
MAFALLSSPSLVSVCDKTFIKPFSLTPPTLRYHKLSYIDQFYSNLYIPLAFFYPKVQQRE
ENELSHIAHLLQTSLSKTLVSYYPYAGKLRDNATIDCNDMGAEFLSVRINCTMSEILDHP
DASQAESIVFPKALPWANNYEGGNLLVAQVSKFDCGGIAISLCLAHKIGDGTSMLNFVN
DWSRMTHSPMTTTLAPKFVGDSVFSSNNYSPIITPQMLSDVSECVQKRIIFPTAKLDAL
RAKVAAESGVENPTRAEVVSALLFKCAIKAASSTTTSMRPSKFVHLLNVRSMLKSRLP
QSTIGNILSSFSTTATKEEDIELPALVRSLRKEVEEAYKKDHVEQNEFMLEVVESMRKG
KKPYDDEYENVYSCSNLCRFPLYKVDFGLGKPERVSLPNGPFKNFFFLKDYKIGKGVD
ARVMLDKQHMSKFERDEELLDLIS

>Solyc02g081750
MAFASLSSPSLVSVCDKTFIKPSSLTPPTLKYHKLSYIDQFYSNMYIPLAFFYPKVQQRE
ESTDNELSQIAHLLQTSLSKTLVFYYPYAGKLRDNATIDCNDMGAEFLSVRINCTMFEIL
NHPDASQAESIVFPKDLPWANNYEGGNLLVAQVSKFDCGGIAISVCLAHKIGDGTSALN
FVNDWSRMTLSPMTTTLSPKFVGDSIFSSNKYSPIITPQMLSDVSECVQKRIIFPTTKLD
ALRAKVIIPQVHIICFYGFFFFFMFPDLFQFMRHNLKVAAESGVENPTRAEVVSALLFKC
AIKATSSTTTSMRPSKFVHLLNVRSMLKSRLPQSTIGNILSSFTTTATKEEDIELPTLVRS
LRKGVEEAHKKDHVEQNEMMLEVVESMRKGKKPYDDDYENVYSCCNLCKFPLYKVD
FGWGKPERVSLPNGPFKNFFFLNDYKIGEGVDARVMLDKQHMSEFERDEELLDLIS

>Solyc02g081760
MAFALLSSPSLVSVCDKTFIKPSSLTPPTLRYHKLSYIDQFHSNMYIPLAFFYPKVQQRE
ESTDNELSHIVHLLQTSLSKTLVSYYPYAGKLRDNAIVDCNDMGAEFLSVRINCTMSEIL
DHPDASQAESIVFPKDLPWANNYEGGNLLVAQVSKFDCGGIAISVCLSHKIGDGTSVLN
FVNDWSRMTYSPMTTTLAPKFVGDSVFSANNYSPIIAPQMLSNVSECVQKRIIFHKIKL
DALRAKVAAESGVENPTRAEVVSAILFRCAIKAASSTTSSMVPSKFVHFLNVRSMMKC
RPSAIGNILSIFSTTATKEEDIELPTLVHNLRKGVEKSYKKDQVEQNELILEVVESMRKG
KKPFDEEYENVYSCSNVCRFPFYNVDFGWGKPERVGLPNGPFKNLFFLKDLKIGQGV
DARVMLQKQHMSEFERDEELLELIS

>Solyc02g081770
MEIEILYKKLIKPFLPTPSHLQHYKLSFFDQIALKEHVPIVLFYANNKFINNFTIDERIKQSL
SKVLTHVYPAAGRYDKDECSILCLDQGISYTKAKVNCKLNNFLEKAHRDLSLAALFWPH
ENKYINKSNLMVSPIVTAQVTEFECGGLAVSLSSSHPAMDGFSNIKFLLEWAKVCKMET
PVENINFLRFNLGNVFPTRDISRLFKSTYDPVIEKDIVTKRFIICETIMSRLRKKCIDEARG
ALTFQPTRVEIITALLWRAFIRTSTIINGYVRPSLMDLPLNLRSKTSLTQVSNSMGNFRV
DVPIKFIPGETKMELHKFIILIRNGVNKVVASCTKASPDEIVSTLVNINNGSVASPEWGGN
DEVDKVSCSSLCNFPFHDIDFGLEKPKLLFFGSKDMQMFWLYDTDIHSQVCVQVDLKE
NYMKLFECNDDIKALTFIHNANL

>Solyc02g081800
MAIAPSQLVSPVTEKIIVKPSLPTPSPLKYHKLSFIDQSLSHLYIPLVFFYSKQQQEFDQL
VANKLQNSLATTLSAYYPYAGRMRDSATIECNDRGIEFLNVRISCPMSEMMNNPHDYA
EGNIFTKDLPWKNSFDGSLLVAQLSHFDCGGIAISTCLSHKVGDGGSVASFIYDWAKIT
RNPNQIARPKFISDTFFPTPNGPLIAPLIDSKLDKCVHKKFHFSASKLQGLRAKIAAEAGI
KNPTRAEVVSALLFISATKAASKINNTSFRPSKLINYVDIRPMTTPPLSRNVIGNLLTVTS
TTASHDEEMELPRLVREFRKEFEQVYKKDPVQHNSLVLKLLEIMESPYAIDEFDTYYCS
NMCKFSGYSIDFGWGKPERVCAPMGPFKNFFILSADQNMDGVEAMVTLEEQHMLAFE
CDEELLEFASPISSF

>Solyc02g093180
MTKLRVVSVCVIRSQKFSSSKKIQLTPWDLQLLTVDPIQKGLFFRKPNPEQPKELAKSS
SEIINRLKVSLSDTLNHFPLLAGRLIASKNSDGTSSFFVDCNNEGAEFNYVTAPDLTVDD
ILEQTYVPKIVKYFFPLNGVCNYEGVSKPLLAVQVTELVDGFFIGCTINHCVADGTSFW
HFFNCWSEIARSGDSNLIVNKPPVLDRWFPEFVASPIHVPLSNEHVHDEFDLPLLEERV
FHFSKENIAQLKAKANSEYGNDQKICISSLQAVLAHIWQSVIRCRCGTNANEKFSFKLLI
GARTRLQPHLPEGYFGNAVHFVNVTTTAREMLEHDSGWAAMQINKVVSTQTHEEVM
NFYQNWVKNPKIVKKSEVVANSLIASSSPRFDVYGNDFGWGRPVGVRSGAGNKHDG
KMTIFCGAEEGSIDIEACLTPETLHAMGQDTKFMGDLSVKIEIA

>Sl DCR (Solyc03g025320)
MTKEEIATTNVNILKKSNVKPQKPLGKKECQLVTFDLPYLAFYYNQKLMVYKLGAESFE
ETVEKLKDGLALVLEDFYQLAGKLGKDDEGVFKVEYDDDMDGVEVIVDEAQEIQVANL
TDHHEGINKFQDLIPYNKILNLEGLHRPLLAVQLTKLKDGLAMGLAFNHAVLDGTSTWH
FMTSWAQLCSGATSISVPPFLERTKARDTRVKLNLSKPSDAPEHAKSETNGDVSASVD
PPMRDRVFKFSESAIDQIKSKVNTNPGEGANNTTPFSTFQSLSAHVWLAVTRARQLKP
EEYTVVYTVFADCRKRVDPPMPESYFGNLIQAIFTVTAAGLLLSNPIEFAAGMIQQAIAKH
DAKAIEERNKEWESSPKIFAYKDAGVNCVAVGSSPRFKVYEVDFGWGKAEIVRSGLNN
RFDGMVYLYPEKNGGRGIDVEISLEANAMERLEKDKEFLMEA

>Solyc03g078130
MKHTATRLYLSQQQYIANFLTMVAIHNCKPLHTYMAPTTKLHAETHEPEIVCDNTGALF
LEPQGKLVLTNQDFPVQVQLTNYKCIGMFTMFTFDYALGDASSFSKFLLVWSQIARRK
SLSFSPDHRRYLLRARNLPIYSPSFDESFISCSLHDIHNISTPRTPKIVSIDGTKRTKIEAF
SAYIWKIMVKAIDKGHKMCKMGWLVDGRTKICNYNKKK

>Solyc03g097500
MENGKHSVAIELTVKQGVPSLVSPAEETEKGPYYLSNLDQNIAVPVRTIYCFKSEEKGN
DNAAEVMKDALSKVLVHYFPLAGRLTISQEMKLIVDCSGEGAVFVEAEANCNIEDIGDN
TKPDPVTLGKLVYDIPGAKNILEMPPLVAQVTKFKCGGFVLGLCMNHCMFDGIGAMEF
VNSWGEIARGLPIKVPPFLDRSILKPRNPPKPEYTHNEFAEIKDISDSTKLYQEEMMYKA
FCFDPEKLEQLKAKAKEDGNVTKCTSFEVLSAFIWKARTQALQMKPDQTKLLFAVDG
RSRFDPSIPRGYFGNGIVLTNALCTAAEIVENPLSVAVKLVQEAVKLVTDSYMKSAIDYF

ETTRARPSLTATLLITTWSRLSFHTTDFGWGEPIVSGPVALPEKEVSLFLSHGKERRSV
NVLLGLPASAMKTFEELMEI

>Sl HCT (Solyc03g117600)
MKIEVKNSTMVQPATETPQLRLWNSNVDLVVPNFHTPSVYFYRPTGSPNFFDGKVVK
EALSKALVPFYPMAGRLCRDEDGRIEIDCKGQGVLFVEAESDGVVDDFGDFAPTLELR
RLIPAVDYSQGIESYALLVLQITQFKCGGVSLGVGMQHHAADGASGLHFINTWSDMAR
GLDLTIPPFIDRTLLRARDPPQPQFPHVEYQPPPTLKTTEENVPNAETVPETSVSIFKLT
RDQINTLKAKSKEDGNTVNYSSYEMLAGHVWRSTCMARGLTQDQETKLYIATDGRAR
LRPSLPPGYFGNVIFTATPVAVAGDLQSKPIWYAASKIHDQLAIMDNDYLRSALDYLEL
QPDLKALVRGAHTFKCPNLGITSWSRLPIHDADFGWGRPIFMGPGGIAYEGLSFILPSPI
NDGSQSVAISLQAEHMKLFEKFLYDI

>Solyc04g009680
MIPIISKKIIKPSSPTPSTQRWHNLSLIDQVVDNLYMPFVFFYSNHQVATIPKHQFSEFLT
NSLSKTLASYYPWAGSLINNATIECDDHGAEFFEVEINSSMNEVIHNPDLTFPKGLSWG
YLSSSTSGVLIVVQLSHFECGGIALSLCMSHKVVGDACSAYFFLRDWARLTREPKLALS
PPYFVQDSLMPSIPFDVPLFPLLLSQKRKDVFKRGSECPPLQMRRFFFYYQIRTISVSP
NIKLAKSNTTTTNQHRKYCHYIFHTNIQNYEHDLKLPKLVTDIRKSKHDLPTRNNLEENE
YVEVMLEAYKTGKLPFHQRNCDIYTITSILAFEFEKIDFGFGKPTRASQESGSFSNLFIL
MNTPDDHDRAVEAFVNLNEQHMSIFKNDKDLLQFATPF

>Solyc04g078350
MASSPTVQHISDCFIKPLYDSEESKKPIYLSIIMGYSHLVVSKHLNKKIPLFTPSLSINCVN
TPGTRFIHASLDSTVSDILSPKDVPLVVQSFLDHDRAINHDGHEFGFGKGVALRSGYAH
KFDGEVSLYEGIEGDGSMDLEVCLLPHFMASLETDKEFMDSLLS

>Solyc04g078660
MASSSPTVQHISDCFIKPLYTSEEAKKPVYLSSWDLAMLSVQYIQKGLLFTKPSSFQLD
PLLQNLKDSLSITLVHFYPLAGRFKTLKQENPPLYTVFIDCVDTPGARFIHANLDLTVSDI
LSPKDVPLVVQSFFDHDRAINHDGHDLSLLTVQLTELIDGVFIGLSINHVLADGSSFWHF
FNSFSQVFKANNGQKQIIPISKSPNFNHWFPEGHGPIINLPYTHHDQFISRHESPILRER
FFHFSSECLKKLKAKANEECHTSKISSLQALSAHMWRCITRVRKFPADHITSCRMAINN
RARLDPPLPENYFGNCIQTVRGIASAGKLLENSLGWAAWEMHEAVVNHKNKDIREWV
EKLESGMIYQLGFFDPSSIMMGSSPRFDMYGNEFGLGKGVALRSGYAHKFDGKVSLY
EGIEGDGSMDLEVCLLPDFMASLETDKEFMDSLSS

>Solyc04g079720
MEVTTGETNTIYPSKPPFTENHVLPLSHIDTDRNLNFTFRYLRVYVNDDTTSDPYEVVT
SSLSAALVHYYQFAGSLRRRPSDNRLELHCQVGDGVPVIPSTVDCTLASMNYLDDPDY
NLAEKLVPDPRDEEALTRPLILQVNRFKCGGWVFGTAVHHAMCDGMGSTLFFHAMAE
IARGEKGMKIEPVWDRSNLLGPRNPPRVEFPVHEFLSLDRDSCPYLESGNSAVRECFE
VKDEWLDRLKGFLHEQSAGSKYTTFEALGAFIWRAKVKACKMSDDETVKYAYLTNIRR

RVKPPLPAGYWGNGCVPIYVQLLAKDIINEPIWKAADAIKKSKDIITDEYVRSFIDFQELH
YDEGITSGNRVSAFTDWRHVGHETVDFGWGGPVTVFPLSRHLVGSVEPCFFLPCSSK
TQGKKDGFKVLVCLQQEAMPVFMEEMEKLEHGLA

>Solyc04g080720
MLGSIDLPDCVYSKEPTFISPISPTPNHTLYLSNLDDQMFLRFSIKYLYIFTKSINLEKLKY
SLSRVLVDYYPLAGRLLKCPQNNHKLQVDCNGKGAIFAEAFLDLSADELLLVSNKPDKS
WRKLLYKDEAQSFLDIPPLVVQVTNLRCGGMILCTAINHCLCDGIGTAQFLHAWAHYTV
DPTVSLSIKPFHSRHVLKPHDPTQINSIHPAFTKIPLDDQNPQFDLNLHQYLQSQPVTPT
SITFSQSQILHLKRQCSPSVKSTSFEVLASHTWRCWVKSLDLPSSVNVKLLFSVNIRKT
VKPELPQGYYGNGFVLGCAEAPVKQVVNGNLQDTVKLVQHAKSELTNDTVKSIVNLLE
DKTVKTDLSTSLVISQWSRLSLEEVNFGEGKPIQMGPLTSDIYCLFLPSLGEIDGIRVLV
SVPENVVKKFEYYMKELWEVNDVNGDIIKGHLQYENPKMISA

>Solyc04g082350
MLISIYCLPKQNKKNYLYKLFQLFNFFTSQNMMKVQVISRENIQPSSPTPKHLKKFNLCL
LDQLIPAPYAPIVLFYPNLNDVKLREKSSLLKKSLAQTLSSFYPLAGRFRDELSIDCNDQ
GVNYVTTNVNCHLIDYLNKPNLESISQFLPCQPPFKVLGVGDYVTNIQINVFECGGIAIG
LCIAHKVLDGAGLSTFLKNWSGLVTCPNLMANYFFPSDDLWLRDTSMIMWSSMFKKG
NFVTKRLVFNDSAIDNLKRMSTSAHIKYPTKVEVVSSFIWKCLIASNKKSNSLLTHIVNLR
KRATPALPENILGNLIWLSSAKNNNAKRYVELADLVNQVRKSILKIDDGYVKRLRGDEG
CSLMRKSLKEIEDFCSKGANHYGFSSWCKFGFYDIDFGFGKPIWVSSISSKCSFFMNLII
LMESNRCDNGIEAWVTLDEEEEMNMLVDNQELLVFASVDPSPLPLYLSG

>Solyc05g014330
MKIEIEIISKLVIKPSTPTPHELHNYKLSYLDQITPNILMPLVFFYQANNNFTKTHISNQLKT
SLSHTLTKFYPLCGRLDVANTHVNCNDEGVPYVEAIAKCNLSDFLLDPLPNELNKLIPC
DLHDVKEFCLLVQANFFQCGGMAIGIAISHKIADALSTFMIINTWGAIARGSIDIPCPRFD
SSILFPPRDVTKFKSSVMIEKDNIVTKRFVFSASKVSALRDKYTEKGTENTRPPSRIEAL
SAFIWTRLMASIHAERDETKIYGMVHTVNLRTRSDPPLPDSLFGNVMQVVVTVPATDS
NDNNSTNKEQDFELVKKVRESINNVNSEFVSGLRKKDQKHLSFIKEKANEQRKGELVV
FNFTSLCRLPLYKADFGWGKPIWVGSARLVLKDVIGFLDTKSGNGIEAWVNLREEDMAI
FEADKELLSWCSD

>Solyc05g015800
MAHNTMPISVTHDKPKLVVPSIVTPHEIKHLSEIDDQGSTRFHVSVLMFYKYNSLMEGN
DPAKIIKNGLSKTLVFYYPLAGRLIEGPNKKLMVNCNGEGVLFIEADANVELEKLGDSIK
PPCPYMDLLLHNVPGSDGMIGCPLLLIQVTRFTCGGFVIGFRVNHTMMDAYGIKLFLNA
LSELIQGASTPSILPVWQRDILRARSPPCITCTHHEFDEQIESKIAWESIADKLIQQSFFF
GNKEMEVIKNQLPPNYGCTEFELLVAFLWKCRTIALDLHPDEIVHLTYLINIRRKLLNFEL
PSGYYGNAFITPAAISKAGLLCSNPLTYAVEMIKKLKDHMNEEYIKSVTDLIVIKGRPQLS
KSWNFIISDNRSGGFDKFDFGWGEPIFGGVPKAVSLISFGVPVKNERGEKGILIAISLPP
LAMKKFQEVVYNLTLRNMQGVNIISKM

>Solyc05g039950
MESVAHPLVSIISEKLIKPSSPTCPTKRWHKLSLIDQAFSNSYIPFSLFYTKDNAISNTSQI
SQLLEESLSKILSTYYPYAGRLKDNTTVDCNDAGAEFIQVQIDCLISETLNWHNTAIEDLL
FPQGLPWSNCASRGLVVVQLTYFNCGGIAISMCISHKIGDGCSGYNLFRDWSHITSHP
NNFSIPSLHYVEQSVFPPPSSGPFLSPLFMSNKHDCVQRRYIFSNQKLLNLKNTVASES
EVQNPTRTEVVSALIFKCAVRAAKANSGIFLPSSMVQAVDLRAQVGLPPNAIGNLLTICP
TSIITNNEESMTISKLVSEMRKSKELVYKRDNVNDNMFVALLLELAKSKREYHDNGPNA
YQITSLVKFALHEIDFGWGKPTKVSIANGLNNKLAILMGNQTGGIDAFVTLTEQDMSVL
QYDTELLEFASLVPSC

>Solyc05g050760
MENVIDSLKVSLSQTLSYLNPLAGRVKDGITTECNNQGVDLACANVHEDMSNVLMNLK
IQVLRKLLPMNPLTRSDDNVLLPLQINCFACGGIAIGVCISHLITDDSSIATFLKTWASISE
SHAENENITISDKLFMDYSSIFPPKEVHSVDTQFLTRVGIN

>Solyc05g052650
MPSATVNLVSKCTIFPSEKSSPNYLKLSVSDLPMLSVHYIQKGCLFTRPPFPIPQLISLLK
INLSHTLTDFPPLAGRFVTDSDGYVYINCNDDGVDFVHATATHICIRDVIGSIDVPHCVK
EFFPLDRTVSYRGHFIPLLAVQVTELADGVFIGCAVNHSVTDGTSFWNFFNTFAEVSR
GVKRIVRQPDFTRDSVLITNSVLKLPADGPKVTFAGDAPLRERIFSFSRESIQRLKAKTN
NQKLNFDGEINIVELIAKQSNDHLKIKTESAEISSFQSLCALLWRAVTRARKFPSSKMTT
FRMAVNCRHRLQPKLNPLYFGNAIQSIPTYVSAGDVLSHDLRWCAEQLNKNVKAHDD
FMVRKFVGDWEKDPRCFPLGNFDGAMLTMGSSPRFPMYENDFGWGRPVAVRSGRA
NKFDGKISAFPGREGGGSVDLEVILSPETMDALESDPEFMQFVNGY

>Solyc05g052670
MPSAAAATHVTLLSKSTISPSEKSSLPDLKLSVSDLPMLSVHYIQKGCLFTHPPFPISQLI
SLLKINLSHTLTRFPPLAGRFVTDSDGYVYINCNDDGVDFVHATATHICIRDVIGSIDVPH
CVNEFFPLDRTVSYRGHFIPLLAVQVTELADGVFIGCAVNHSVTDGTSFWNFFNTFAEV
SRGVKRIVRQPDFTRDSVLISSSILKLPADGPKVTFDGDAPLRERIFSFSWESIQRLKAK
TNNQKLNFDAEINIVKSMEKQSNDHLKIKTESAEISSFQSLCALLWRAVTRARKFPSSK
MTTFRMAVNCRHRLQPKLNPLYFGNAIQSIPTYVSAGDVLSHDLRWCAEQLNKNVKA
HDDVMVRKFVGDWEKDPRCFPLGNFDGAMLTMGSSPRFPMYENDFGWGRPVAVRS
GRANKFDGKISAFPGRKGGGSVDLEMILSPETMEGLESDTEFMQYVTGF

>Solyc05g052680
MTTPPVALVSKCTVFPSEKSSLPDLKLSVSDLPMLSVHYIQKGCLFTRPPFPIPQLISLL
KINLSHTLTRFPPLAGRFVTDSDGYVYITCNDDGVDFVHASATHICIRDVIGSIDVPHCV
NEFFPLDRTVSYRGHFIPLLAVQVTELADGVFIGCAVNHSVTDGTSIWNFFNTFAEVSR
GVKRIVRQPDFTRDSVLISNSVLKLPADGPKVTFAGDAPLRERIFSFSRESIQRLKAKTN
NQKLNFDGGINIVELIAKQSNDHLKIKTETAEISSFQSLCALLWRAVTRARKFPPSKMTT
FRMAVNCRHRLQPKLNPLYFGNAIQSIPIYASAGEVLANDLYWCAEQLTKNVNAHDDV

MVRKFVEDWEKDPRCFPLGNFDGAMLTMGSSPRFPMYDNDFGWGRPLAVRSGRAN
KFDGKISAFPGRGGGGSVDLEVILSPETMDALESDPEFMQYVTVY

>Solyc06g051130
MSSSVHVKEATLITPSDPTPIQVLPLSALDSQLFLRFTIEYLLVYKPSRHVLDKLATVSRI
KAALGRALVPYYPLAGRVRARTNGSAGLEVVCRAQGAAFIQAASDLTAEEFEGAPRH
NTQWRKLLSLQVTDVLKGAPPLVVQLTWLSDGSATLGVGFNHCLCDGIGSAEFLNLFA
ELATGKQKFTQLNRQVWNRYLMDPNIYNKRIYQQNHPEFNKVADLCNFNSRFSQLAP
TSVTFNRSRVNELKKLLTYTQSSCTSFEVLSAHIWKSWATSLNLPPNQTVKLLFTINIRN
RVKPSLPIGYYGNGFVLGCAQSCARDLVEKGLGYAVGLVKRAKDRVDDEYVREVVES
VSSNGTSPDSVGVLIMSQWSKLGLEKIDIGMGRPVEVGPVCCDRYCILLPVYDQKDSV
KVNVAVPTSAVDKYLYLLNNTTTIGT

>Solyc06g051320
MDVKVISEEIIKPCIPTPRHLRNYKISFIDQFTPCSYIPVILFYNANDDVDHELKPMQVALA
ETLSYYYPLAGRFKDVYSIECNDEGVVYVEAQANFNLSKFLQNPDIPFLNKFLPFKGNC
LEPSYNQPLVALQTTAFECGGMAIGVCMLHKVVDASTMSVFLKTWAKISRGEGDKTLH
PDFTSAISLFPPIDSLPTKFITDFDNFYFQGSKSPMRRFLFDSKSIKALKANTSSESVPFP
SKIEALTAFICKRIGAALMANNVPKTLMITHAANLRPRVDPPLPQKTFGNLLWLAFAFYD
PLDTNNELPDLGIMLREVFAQLTAENIKDIDSECVFESLSEVLESLSTNENIKTYRFTSW
CNMGLYDVNFGWGKPVWVAHMGDLPAANVRSKQQFVFIESASRQGIELWVASDEEEI
RFLEKDAEFLAYANPNPSICIN

>Solyc06g071940
MVMISTTTSVEVVSRETIKPLCPTPNHIRNYILSSIDQNSPPIYIPVLLFYLNNTSINTNEK
EKAYEREVIVAAQANLFRYDFFVFYVEMYKGGVPTRVQAVSSLIWSRILALYRSKPKYA
KICVAVHAVNIRPRMQPPVPSHTFGNYWTVAIAPAVVKTEQYAAEMSNDITILVEKK

>Solyc06g074710
MKVKIESSKIIKPFYEDNIIPPSTKTYIPLSVFDKVTYEAQIAIIYAYRPPTPPNAAIQLGLQ
KALAIYREWAGRLGKDENGIPVISLNDEGVRFVEASADSTLDKVMPFKPSASLLNLHPR
LNNVVELVQVQVTRFTCGSLVVGFTAHHTVADGHSTSNFLVAWGQACRGLDINPLPL
HDRTIFNPRNPPLIEYEHKGTEFMSKSVKKENSLDESTHHVTEDIVVHKVHFTVEFLAKL
KVKASSMNNNNNSNSNRPYSTFESLVAHLWRAITKARGLSGFETTHIRISVNGRMRLN
PRVPNEYFGNLVLWAFPTSKVKDLLREPLPYATKLIHDAVVKVNNNYFRSFIDFANHKD
EDLIPTADMNKHILCPNLEVDSWLRFPFYDLDFGMGCPYMFMPSYFPTEGMMFLLPSF
IGDGSIDAFIPLFQDNLPTFKKICYSLDLA

>Solyc07g006670
MKITKICEELIKPSSPTPIELRDHKISFIDELIPHSSIPLILFFKKSENITRSQICNHLKSSLSQ
TLTQFYPLAGRIKSQYSIDCNDEGAYYQESQVDVSLLDIIKNPKSNELVQLTPYNSNGTL
SNFQELLAIQVNLFTCGGIAISISISHKIGDASSLCNFIKYWSNACEGLIRSRTRSPEMLL
HSRQSTFLKNPSDIENDKVRKDSVFISLPSIFPSRGTIDNTLNVNSMPLRIVPEKLAVKRII

FTSSNIVKMKAKLVNWGYNENATRVEVIIGLLWKCFMVAKGCNSVAIIPVNIRQRIVPPF
DENSFGNFFLVTSCMASVENEWCSLVGKIKSAIGSIDGNYVEKIRGDDGFEFVDSNFR
QVGKLMRSQGDDFRVLTISSWCKFPIYEANFGWGESILTIVATLGVKNNIALLDSKEFP
GGIEAWVVMADQEMTLFEQDKELQHFTSLDAIGDLN

>Solyc07g006680
MQISKICEELIKPSSPTPHELRDHKISFIDERIPHSSIPLILFFKKNENITQSQICSHLKSSL
SQTLTQFYPLAGRMKSQYSIDCNDEGAYYQESQVDASLLDIIKNPKSNELVQLTPYNSN
GSSSNFQELLAIQVNLFNCGGIAISISISHKIGDGSSLCTLIINWCTTSERLIRSNTHLSIFL
KSPSNTGANKVREHSNFVSISSTFPPRGICDDTPKEKCIPIHPVAENLAVKRFIFTSSNIA
KMKAKLINWGYNENATRVEVILALLWKCFMSAKGCNSVAIIPVNIRQIIVPPLNENSFGN
FFLVTSCIASVENEWCSMVGKINSAIRRIDGNYVEKIRGEDGFEFVNSNFKQVGELIMS
QGDDIRVLRISSWCKFPIYEANFGWGEPILTIVAFLGVKDNIVLLDSKEFLGGIEAWVVM
ADQEMTLFEQDEELQDFTSLDAIGDLN

>Solyc07g008380
MESKSLTKIQILSKNIIKSNHVNDVDHPNNYKLSFFDQFACQMHVPCFLFYPIKYSTSPKI
SIIHEQLQQSLSKLLSHVYPASGRFSSDAQSINCHDEGVLYIKAKVDSQFCDFLKDAQK
DIDLALNFCPKINRNDSNLSLTPLVVVQVTEFACGKGLALSLSAEHAVIDGFTALKFVYE
WSKVSKMGINKINCFTFDDFGTIFPPTSDNHLLKRVESPRDDHNHDFPEMVARRFVIN
QSVISKLREHVGVVHIRPSRVELVIAFLWRALINVYRCKSNGRLRPCLLSVPVNLRGKID
FPRYENSFGNFAIEVPVKFIPGETGMELKDILLLIKDVIQKTNVSFVKSSDNIYSLASKFH
EEIKEWEENEQVDVCMASSLCRFPINEADFGWGKPCLLSFGLRRSDMFWLYDTQCGS
GIVLQVDLKKEYMDMFGCDKDVLSFIFDE

>Solyc07g008390
MLKHRLCSSIHVYAIRYTCLLLSYLMESKVLTKIQILSKSIIKSNHDNNVDHPKIYKLSFFD
QFALQMHVPCVLFYPLKNPTFTKTPIIHEQFQQSLSKLLSHVYPASGRFSSDGQSINCH
DEGVLYIKAKVDSQFCDFLKDAQKDIDLALNFCPKVDRNDSNLSMTPLVVVQVTEFAC
GKGLALCVSSEHAVIDGFTALKFVYEWSKVSKMGINKINCFTFDDFGTIFPPTSDSHLLK
RVESPRDDPNHDFPEMVARRFVINQSVISKLREHVGVVHIRPSRVELVIAFLWRALINV
YRCKSNGRLRPCLLSVPVNLRGKIDFPRYENSFGNFAIEVPVKFIPGETGMELKDILLLI
KDVIQKINVSFAKSSDDIYSLASKFHKEIQEWEENEQVDVCMASSLCRFPINEADFGWG
KPCLLSFGLRRSDMFWLYDTPCGSGIIVQVDLKKDYMDMFGCDRDLLSLTCE

>Solyc07g014580
MNSKKNFNVKVHKSEVVAAVLPMQEHWLPLSNLDLLLPPLEVGVMFCYLNPIIVSEKIN
MNLEFNSIVKILKTSLAETLVSYYAFSGDIVENSAGELEILCNNGGVSFIEAFGAVELKEI
NFYNPDESIEGKFVPKKKHGVLAIQVTQLKCGGIIVGCTFDHRVADAYSANLFLVSWSE
LAQSKPLSQLPSFRRSSFFPRHPGYYDDSIDDLYVPLSTLPPMKSEILNPNDQVISRIYY
VTGGKIEHLQSLANCHDDQKGTSQRSKLESFSAFLWKTIACGINKETWGFNNFRFGIM
VDGRTRLIINNVDKSLKGYFGNVLSIPFGEKKVEEVKEKSLNWVANVIHEFLDIAKTQEH

FLGLTDWVEAHRPKPSMAKIYAMNGDGPAVVVSSGQHFPAKKINFGWGEPAFWSYH
FPWAGKAGYVMPMPSPKGNGDWIVYMHLLKWQIELIEASPYEVFKPVTANYLNLI

>Sl SHT (Solyc07g015960)
MKVILKNQWVVKPAEATWNGTVSLSEFDQTFALTHVPTIYYYKYFNDFVTDEIVDTLKIS
LSKALVYFYPLAGRLRWINGSRLELDCDASGVVLTEAEADAKLDNLTDFLLSPDYNSLF
PRVDYTVEINELPLLFVQLTKFQCGGIALSFAISHAVVDGQSALYFFSEWARIARGEPLM
FSPCHDRKLLRAGEPANASPTFEHLQFNTPPLLIGKSSTENEKKKTTKGAMLKLTKHQV
EMLREKANQGRCLEDSKERSYTRYEVVTAHIWRCACKARGHKFEQPTNLCICVNIRQK
MNPPLPNTYFGNAIVDVIATGASGDIASSPLECAAKRVREAINMVTSDYANSTINFLKKQ
EDLSIYQDIHAFRNKEGPFYGNPNLGVISWISLPVLGLDFGWGKEIHMSPGIHEYDGDC
VILPENEGDGSLTVAIVLQHAHVDAFEKFFYEDIEC

>Solyc07g017320
MQVTQLKCGGIIVGCTFDHRVADAYSANLFLVSWSELMKSEILNPNDQVTSRIYYVTGD
KIEHLQLLANCHDDQKGTSQRSKLESFSAFMWKTIACGINKETWGFNNFRFGIVVDGR
TQLIINNVDKSLKGYFVNVLSIPFGEKKVEEVKEKSLNWVANVIHEFVDIAKTQEHFLGLI
DWIEAHRPEPSMAKICAMNGDGPAVVVSSGQHFPVKKINFGWGKPALWSYHFPWAG
KSGYVMPMPSPKGNGDWIVYMHLLKWKIELIKASPYHVFKSVTANYLNLI

>Solyc07g026890
MFVSIVVCLTVINSCDNDRSGKNCVFSQGLYFGNSSDSFDGRLAMIQLTHFDCGGIAV
NFCFSHKIVDGYSAGKFMSDWAAISKDLNHAIPYPRFDGASFFPPKVMFLQ

>Solyc07g043670
MAASQLALISKKIIKPSSPTPLIHRIQKLSIFDQLNPCSYMSYGLFFPKQCHPTLPDDPTHI
STILENSLSRALTSYYPLAGTIRDEGLHVECNDVGAKFLQVKIDCPMSQIFDQKSTEAED
LVLPRDLPWTPSKENLVVAQVNHFNCGGIAIGVCVSHKVVDGSSMANFTHHWSTMAR
NPSAPIPSFQFVGGSHFPPVSSPIAEAILQSKTRGNNTCISKKYLLSRSKINSLKAMIAAE
SGNKSSSITPSSVEAASAFVYKCIASNSTKPSIFTQSVNLRPIMKKWLPEEFQGNASFLF
MAPEISDPADIKLHRLISELRKEKEMYWETTNLMSTLLEKVKLLQNQLERDHDDEVDVY
KCTSLSKFIFGQMDYGWGSPTRVCLGSVPINKKIFLTDSQTEDGIEVVVSLKQQQMSAL
ETKSNLLEFASPL

>Solyc07g043700
MVTPKSHIVSMSKKIIKPSSPTPSSLRHHNLSFLDQINSPLHNPLAFFYPKPKNSSYDIN
HISTLLQNSLANLLTSYYPFAGTYNDNIFIDCNDVGAEFFDVQLDIPMSEILDHPYYNDDI
NLVYPQGCPWNIYDGTLVVAQLTHFDCGGIAVSVCISHKVADAYSVIKFITDWAYLTRD
SSHAKPSVLFDGTSFFPPVTDTSTLPDFDAGNDRNCVTRMFHFSSSGLDKLKTIAAAE
SGVENPSRVEVATALIHKHAMIASGSFKPSLLCHIMGLRPPLPLNSIGNACSFFLTSTQT
EDEMELSNSVAVLRKSKDQLREKMKNATQSDIFFKVVEITKQGAELVEKVNKADVYKC
SSLCNFGLYDMDFGWGKAIRVSSVSRAVKNQIFFMDSPTGNGMDVLISLDEKEMEIFQ
SDKVILEYASPVVKM

>Solyc07g043710
MGSISRIVSMSRKIIKPSSATPPSHKRHNLSLLDCYANNEYASSVMFYPKPTIPSNNISQ
LLQKSLSKALTYYYPFAGMLKDNTYVDCNDRGAEFLNVRVDGRMSDVINSCDNDRSG
KNCVFPQGLCFGNTSDSFDGRLAMTQLTHFDCGGIAVSFCFSHKIVDGYSAGKFMSD
WAAIAKDPNHHAITYPQFDGASFFPPGIHDAGTSEVNSLPEEGLVQQRFVTRRYLFSA
SKLNTLKAKIVDSGSGIVGNPSRIESVSALLYKSAAANRNNTSFRPSTFTLFANMRPPLP
LNTIGNAPGYISTSIEEEVDMQLPRIVAELRKGKEKLRNRFNTVDPNKLVFESFELMKEV
GQVYNREGFDIYRCSSLCNFPFYGIDFGWGTPQRVAFLIPPCNFFVLLDNQNRDGGVE
ALVTLEESEMTVFQQDQQILQFARPI

>Solyc07g049660
MDSIKPSSSQLVFTVRRQNAELIAPAKPTPRETKFLSDIDDQEGLRFQIPVINFYRKDSDI
STGGNHDPVKVIKKAIAETLVFYYPFAGRLREGNGRKLMVDCTGEGVMFVEADADVTL
EQFGDELQPPFPCLEELLYDVPGSAEVLNSPLLLIQVTRLKCGGFIFAVRLNHTMSDAA
GLVQFMTAVGEMARGASVPSTLPVWSRELLNSRDPPRVTCTHHEYDEVPDTKGTIIPL
DDMVHKSFFFGPSQVSALRRFIPPHLRKSSTFELLTSVLWRCRTISLKPDPEEEVRVLC
LVNARSKFNPPLPNGFYGNAFAFPVAVTTAAKLCKNPLGYALELVKKAKSDVTEEYMK
SVADLMVMKGRPHFTVVRTYLVSDVTRAGFGEVDFGWGKAAYGGPAKGGVGAIPGV
ASFYIPFKNKNGENGIVVPVCLPAFAMEIFVRELNGMLKSEEDPLGKYTNYAIIKPAL

>Solyc07g049670
MDSIKQSSSQLVFTVRRQKAELIAPAKPTPRESKFLSDIDDQEGLRFQIPVINFYRKDSY
SDLDSSMGGNYNEPVKVIKKAIAEALVFYYPFAGRLREGNGRKLMVDCSGEGVMFVE
ADADVTLEQFGDELKPPFPCLEELLYDVPGSAGVLNCPLLLIQVTRLKCGSFIFAVRLN
HTMSDATGLVEFMTAVGEMARGASAPSTLPVWSRELLNARNPPRVTCTHHEYDEVP
DTNGTIIPLDDMVHKSFFFGPSEVSALRRFLPPHLRKCSTFELLTSVIWRCRTISLKPDP
AEEVRVLCLVNARFKFNPPLPNGFYGNAFAFPVAVATAEKLVKNPLGYALELVKKAKS
DVTEEYMKSVADLMVINGRPHFAVVRTFLVSDVTRAGFGEVDFGWGKPVYGGPAKG
GVGPFPGMASFYIPFRNKNGENGIVVPMCLPAFAMEIFVKEIERMLKGDVNPLVNTNY
AIIKPAL

>Solyc07g052060
MEIEILCTKLIKPCLPTPPHLQRYKLSFFDQISEKEHVPIVLFYANNNNFFNTSTINERIEQ
SLSKILTHVYPAAGRYDKDECSILCLDQGVSYTKAKVNCKLYNFLEKSRKDLSLAALFC
PHVNKYIDKTNLMVSPIVTAQVTEFECGGLAVSLSFSHPAMDGFSDFKFLFELARVCK
METPIENIKFLSFNLGNIFPTRDISRLFKSTFDRVIEKDIIVKRFIVREAAMSRLRKKCIDEA
RGALDFQPSRIEIITAILWRAFIGASTIINGYVRPSLMDFPLNLRSKSYLPQVKNSMGNFR
IDVPIKFIPRETKMELHHFVILIRNAVDKVVASCTKASPDEIVSTLVDIYNESFEAPEWGG
NDEVDKVLCSSLCNFPLQDTDFGLGKPTLVFFGVKDMQMFWLHDTDIRSEVGVQIDLK
ERYMQSFQCDDDIKDLTFIGNANL

>Solyc08g005760
MAHTMPISITYEETKLVVPTITTPHETKYLSEIDDQGSTRFHSHLLIFYKYNSLMEGKDPA
KIIKDGLSKTLVFYYPLAGRLIEGPNKKLMVNCNGEGVLFIEGNANVELEKLGESIKPPC
PYLDLLLHNVPGSDGIIGSPLLLVQVTRFSCGGFAIGFRVNHTMMDAYGFKMFLHALSE
LIQGASTPSILPIWQRHLLSVTSLSPNITCTHNEFDEETPSKIAWESIEDELIQQSFFFGN
KEIEAIKNQLSPYCGSTKFELLVAFLWKCRTIALDLHPEEIVRLTFFVSIRGKLQKYKLPS
GYYGNAFISPATISKAGLLCSNSLTYAVELVKKLKYDMNEEYIKSLTNLMVIKGRPKLSK
SWNFIVSDNRFVGFDEIDFGWGKPIFGGVSEKISLISIGIPINNEKGEKGILVAISLPPLAM
EKFQKIVYNMNFKNVGTNIISKM

>Solyc08g005770
MANILPISINYHKPKLVVPSSVTSHETKRLSEIDDQGFIRLQIPILMFYKYNSSMKGKDLA
KIIKDGLSKTLVFYYPLAGRLIEGPNKKLMVNCNGEGVLFIEGDANIELEKLGESIKPPCP
YLDLLLHNVHGSDGIIGSPLLLIQVTRFTCGGFAVGFRFNHTMMDAYGFKMFLNALSELI
QGASTPSILPVWERHLLSARSSPSITCIHHEFDEEIESKIAWESMEDKLIQQSFFFGNEE
MEVIKNQVPPNYECTKFELLMAFLWKCRTIALNLHSDEIVRLTYVINIRGKKSLNIELPIG
YYGNAFITPVVVSKAGLLCSNPVTYAVELIKKVKDHINEEYIKSLIDLMVTKGRPELTKS
WNFLVSDNRYIGFDEFDFGWGNPIFGGILKAISFTSFGVSVKNDKGEKGVLIAISLPPLA
MKKLQDIYNMTFRVIISNI

>Solyc08g005890
MEEIQVVSSCLVRASSNSQNVNLNIEITPWDLQFLLVDTIQKGLLFHKPKKENIDNLKKS
LSHTLHFFPPLAGRFSIVKNSDDETISFFINCNNAGVEFIHANASKLSVSMIIDSTYVPNII
HSLFPLNNIRNFEGVTKPLFGVQITELVDGYFIGCTMNHTLGDGTCFWHFFNSWAEISR
GRKIISRVPILERYFPEKIHVPIHFPMNLDDEKLFEKIEIPTLVKRVFHLSKENITRLKKKAN
DEMSINSISSLQAYLAHLWRCVTRCRKIDDREEVYIGILIGARSRLKPPLLEGYFGNAVHI
KIVKTSARELLENGLGWVAMQINKMVVSQNSKEVIKMYKGWIENPIIFNKTRVFVDETR
LSIGSSTRFNIYGNDFGWGKPIGVRSGMANKRDGGITLFQGANEGSVDIEVCMCPHTL
QAMENDQEFMEMVTN

>Solyc08g007210
MATNCNGNGAPFFSPKLQVEAIQTVIPMKITDPRFSRRIAIPENFHLGNLQRRFHMILYY
NKASETDSGWIVAGWFKESLGSAIVENPIFAGRLRKLEDDYELVSNDSGVRMLEANFP
MNMVDFIDHKKNENIENELVYWEDIHETSPQFSPLFLIQVTNFKCGGYSIGISCSLFLAD
PFAMTSFLKSWSKIHNNLVSQIDSPKIPAFYTPNFTKIGSSPTLSTTSPKTKNQLTNTSIF
KYPKTPLKMNNNEFIKNLASKCIGEIEEKIGKNISSNFTFLVKENFESIKVENCSKEEIIKE
EKSGLLSIISTNWGDLGANKVRFTEGNEAIHISCWIINGENNQDLVMISPTNDEDNSELN
IIITISN

>Solyc08g013830
MTPMVQELNFPQLQIPLTINSISPILPSSPIPPSYGDTLYLSNLDDMIGSRVFTPTMYFYR
SNGKMDVMTIINVLKEALASVLVPYYPFSGRLRETKEGKLEVFFGPKQGVLLVEACSE
MKILNLGDLTVPNPAWKNLVYTFPNEEQYKVIDMPLLIAQVTRFSCGGFSLGLRVCHCL

CDGVGAMQFLSAWASTARLNKLTLDPKPCWDRETLIPNDPPFIQYPHIEFKRIDDAFSF
TRRLFVQKCYRVTRDFQAHLKTLVGPNISCTTFDAMAAHVWRSWVKALINVSPLDYEL
RLTFSVNCRSRLTNPPLKSGFYGNAVSVACATSTVSGIVNGSISDTICLVRNARLSVSK
AYLRSTIDYIQVNRPTKLEFGGKLTITQWTRFSMYESADFGWGKPIYAGPIDLTPTPQV
CVFLPQDNSDGAMLVCICLPEDVSHRFTDIFCLLN

>Solyc08g014490
MASPALKILSRASKKLIKPSSPTPLTQKWHKFSLIEQAQTHTYVPFGFFYSNNQLGALS
NQPSQTSKLLENSLSKNLVSYYPYAGHMKDNAVIDCNDKGVEFLNVHIDAPMSQVLND
KDCHVKDLIFPHGVAWENDSEYGLAVIQLTHFDCGGIAVSTCLSHKIGDACNGLQFLID
WAKLTRDPNAKITPPYYISDTIFPSPPTGPLDSPVVPSILDGCTQKRYVFSSSKVSELRS
SIASVSQVKDPTPTEVLSALLFKCVAKAVTANSGSFVPSKLIQYADLRGMISPKLPPNSI
GNVLSHFSTHISNEADMNLPQIVSLMREEKLLFRTRDNIKENAWALEILELAKGLPPKKK
EFDEYTCSSVCKFPFYDVDFGWGKPMAATIATGPYNKLFNLMNYKDDGVEAFVVLDE
QDMSVFERDEEFLEFASPYANYF

>Solyc08g036440
MSNPFDLLTVNITPEPEKDNDMVEGFNKRVLLFFFFHDNFFKTAFSAECQEKKVKFYG
RSRFDPSIPQGYFGNIIVLTNALCTAAKIVKNSLSTAVKLVQEDVKLVTDSYMKNKSFLS
TRRISVGKAYCIRAVALPEKEVSLLRSHGKERRSVNVLLRLPASAMKTFEDLWRSK

>Sl GAME36-like-1 (Solyc08g075180)
MANFVSDWASIAREANIIPSPTMTGSSIFPPCTNLPSTDIYTNTIVNVPIENIKKRYRFSN
SKLEMLKSQVTSEAEVQNPSRVDVLSALIYKCAVTAARAQANSFKPSMLTLAVNLRPIL
DPPLATRAIGNMVSFIKVETTSVDEITIAGVVRELRKAKDEFKKEDHVNANKLVALHSEN
LPISNEFETYRSHSMCNFPLNNLDFGWGKPNKVTIPPIGVGFCFILMDSPSGDGIEAIVA
VPETYVTQFENNKELLQFATPIN

>Sl GAME36-like-2 (Solyc08g075200)
MAAASRLVSFAEKIIKPHRATPLSLGRYNLSINDQIMVPFYQSIAAFYPNPSKTPEQVSSI
LENSLSKVLSSYYPFAGTLRDNTFVDCNDRGAKFMNVRYDCPMSEIAKLPDTGPEYLP
FAKGIHNSLLLGDEDLLVVQLSHFNCGGLAIGTSILFYRKNDLSKREFVLFSTFWESPLN
FEKIKKTCFQIIKQKIV

>Solyc08g078030
MANCNENIGVAHDFVSNLQIEAIQTVMPMKQTDPRISRRVFIGENPGSGNFQRRFHMV
FCYNKVSETDSGSMVAGWIKESLGKALVEKPLLAGRLRSIGENDTNYGEFEIVSNDSG
VRLIEAEIQMNFDDFIHLKEKKNIEGQLVFWDDIHEPNTPYSPLFYVQVTNFKCGKYSV
GISCSLFLEDPYSMTSFLNRWSKIHINMVSEADAPKIPTFFLPKLRRKGCSSPTLYSSLN
TSNYHVNDTLIFKLPLKILNLSDDINKNNLVEKCVEEVENKYGKNLSTKLCLFVRETSED
VNVETFTREGINSFGSIKNGLISANYLDDLGLADNMRFNEENKAIHFSCWIINPGNEDLL
LITPSPGDESGSQKNVIVTMRD

>Solyc09g014280
MPTSSLFVVSKCTIYPDHHTSLNNTLKLSVSDLPMLSCQYIQKGVLLTQSPFESSNSLIS
HLKTALSKTLSHFPPLAGRLITDSDGYVYILCNDAGIDFVHTKAPHLMISTLVPQNQSDIP
IYFRKFFQFDKTLSYAGHHKPLMAIQVTELNDAVFIGCTMNHAVVDGTSFWNFFNTFAE
FSKGEMKITRQPCFGRKTVFDSPAVLKFPEGGPSATFSGDEPLREKIFHFSREAILKLK
LRANKTGVNGKTNGKITPVNDETGLKNGAAMKNSASEISSFQSLSAQLWRSVTRARKL
EGNKTTTFRMAVNCRHRLEQRLEPLYFGNAIQSIPTIASVKELLSNDLYWSANQLHQNV
VAHGDVTVRQGVKDWESNPRLFPLGNFDGAMITMGSSPRFPMYDNDFGWGRPVAV
RSGMANKFDGKISAFPGREGNGTVDLEVVLAPETMVGLEEDMEFMQYVS

>Solyc09g092270
MVSSKLEDGLIYNIKLSSVGPARVTGQDVVYEPSNMDLAMKLHYLRGIYYFDSQAFQG
VTIYKIKEPIFIWFNYFYMTNGRFRRAESGRPYIKCNDCGARFIEAQCDKTLDEWLEMK
DTSLEKLLVSNQVLGPELAFSPPVLIQHTKFKCGGISLGLSWAHVLGDIFSATEFWNFL
GKVVGGYQPPRPINLAHSLTKANSTETLQKIVEDPLSIKRVDPVEDHWVANASYKLESF
SIHVSASKLGQLQSRIGIQGPFESLCSIIWQSISRIRDGPGPKVVTICKKGEEKKEGGLIG
NTQLIGVVKVDDSIRDANPSELARLIKNEIIDERLKIDEAIEKEHGVSDVIVYGANLTFVSL
EGVDLYEFDWKGHKPKNVSYFIDGVGDAGTVLVLPDGPNYAEGRIVTMTLPVDEIMKL
KNELNNEWSIA

>Solyc10g008670
MATVIEQCQVAPPPGGATEVILPLTYFDHVWLGFRRMRRILFYKLSISKPDFVQNIIPPL
KNSLSLTLKHYTPLVGNIACPLNSSGYPELCYVTGDSVSDTFTETDMDFNHLIGNHPRN
ANDFYPFIPQLAQPKDAPGFKLVPVLAIQVTLFPNLGISIGFSNHHVACDGNIIVKFIRTW
GLLNKLRGDEQCLANELIPFYDRSVIKDPYKQGTIIWDEMKQNMPEIGDIIVIPPLDRVR
GTFIMERNNIVKLKNLILSRRPNLSYVTSFTITCAYIWTCLIKSKFAIEDEMIDEDVMEIFG
CVADCRSRLNPPLPQSYFGNCLVTIASKASRVELVGKEGFITAVEVIGEAIKSQMKDVE
LILNCSWYREFCGINMKHTLSVSGSPKFNLYEVDFGWGRPEKIEIISIDNSSGISMSISKY
KDSHGDLEVGLSLPKTRMNAFVAIFNHGLSFL

>Solyc10g008680
MVSIIEQCQVAPPTCAIDKLTLPLTCFDTLWILHNHHIPRILFYKLQNIDKNSFIQNIIPTLK
HSLSLTLKHYLPLVGNLVCPWNSTGYPELRYVPGDSVSVTFSETDKDFDYLVSYDHIH
NAKDFHPFVPKMAEPKDASGVQFTPVLAIQVTLFPNHGISISCISHHVVGDESTIVGFIN
SWALLNKNKGNDDHDDKFIVPFYDRSIVKDPYGLGDCIWEETKKHKKEMMSDIIVTPPD
NNNNMVRGTFTIRRDHIEKLKNLILLTRPSLAHVTSYTVVGGYAWSCLVKSEGANEIINE
NVMECFGCGVNYRARFDPPLPSSYFGNCIIWYIASKKHVDLVGDEGFRVKLVGHPMK
GDIFAIAGSPKYDLYEADFGWGKPQKWHFVFFGSGLLMSLGKSKDSNGDLEIGLYLPK
TRMDAFATIFSDGLTVL

>Solyc10g055730
MTKLEVVSTCVIKSQKSIEKKIELTPWDPQVLTLDPNQKGLLFRKPNPKELAKSSSEIIVN
HLKISLSKTLNCFPLLAGRLVARKNIDDDTLSFFVDCKNEGAEFAHAIALDLNVENIAQLK

AKANSEYGNNIVCISSLQALLAHLWQSVIRCRFSTNAAEITYRSCIYLKDILEMPCILSTS
QPPLRRYWSWVALQMNKVISTQTHEEVMNFYQSWVKKSKMLKKSGVFANSLIASSSP
RFNVYNNDFGWGRPVAVRSGAGNKHEGIITIFCGAEEGSMDIQPQTLRAMGQDTKFM
AAVTKIHDIV

>Solyc10g079570
MVSSLVSLISKKIIKPSILTPPTKKSHKLSFVDQVMHMSIPMAFFYPKIGNYEPTHVPQIL
ENSLSKLLSFYYPYAGRLNKDDANYVDCNDMGVELSHVHVHCPMSHILRQPYTNSDH
VVFPVEQPYAHMNEGNLATTQLKSRVNQKFKILLEVVSALLYKCALVARGVNDSGSFK
PSSLFQVANMRHRLNPPLSYDTCGNILSGYFVETNNEKDVNCSKMVGEMKKGKLNLH
PKESALVKSIKKGKTPFYSNDEVDNYFCSSLIEFPLYKVDFGWGRPIRVGMGAGPFDK
FFILLDNQSGDGVEVIVMLDEQNMAIFERDLELLEFASPITNL

>Solyc11g008630
MGTLYQPLESTIQDLKVTIHNTSLVFPSQETPKKPMFLPNIDQVLNFDVQTLHFFNANP
EFPPEIVTERLRIALSRVLVPYDFLAGRLRMNQESKRLEFDCNSDAGAVFMVASSELTL
NEIGDLVYPNPGFRQLIVHENIDILEKDDKPLCILQVTSFKCGGFAMGFSTNHITFDGISF
KTFLQNLASQAFDDDNNNPKPLAIVPCNDRTLLAARRPPRVTFPHVELLKLDVPIGEEL
NAKVFETLQEELDFKIFKFNPSDINSLKEKAKDENTPNAKITSFNVVTSYVWRCKALSYD
DENNSERVSTVLFAVDIRPRLNPPLPQSYAGNAVLTSYASATCHELEEGPFSKIVDLVS
QGGKRMDDEYARSAIDWGEINKGFPNGEFLLSSWWKLGFSQVEYPWGKPKYSCPVV
CHRKEIILLFPNIDDGKSNNNDGVNIFVALPPKQMNKFESYFNKFLLD

>Solyc11g020630
MVINRRRPWTKSWNFLITNDAILGLDEVDFGWGKPILGGVDRSPFSFASFFWSFNNNI
GEKSIVIAINLPKEAMEKFQHLIHDFTSKNVEKIQLASKI

>Solyc11g020640
MAIIPRPLPISYSRRKPELIVPKNKIPKEILYLSDIDDQEGLHFQMPILMFYKYNSSMKGK
DHAKIIKDGLSKALVFYYPLAGRIIEGPNRKLMVNCNSEGIMFIEADANVELEKLGDSILP
PCPYLEELLYNEPGSVGIIGCPLMLVQVTHVTCGGFVVGFKVNHTMMDAYGFKMFLNA
LSEIIQGASAPSILPVWQRDLLSARSSPCITCTHNEFDEQVESKLAWIAMEDKLIQHSFF
FGNKEIKAIKDQLLQPGTKKKWNYHLDIMAMHLQHQQPYQKLDCYVQIH

>Solyc11g066640
MKIKIESSRIIKPFYEHTPPSTTSHIPLSVFDKVTYEAQIAIIYAYHPPTPPNIAIELGLRKAL
VVYREWGGRLGEDEHGNRVILLNDEGVRFVEASASSTLDQAMPFKPSPSLLSLHPSLK
DVKELVQVQLTRFTCGSLVVGFTAHHTVADGHSTSNFLVAWGQACRGLKVNPLPLHD
RSIFTPRNSPLFEYQHKGVEYMSKSKKEHSLNEVHHISEDVVVHKVHFTVQFLANLKAK
ASSMNGNNKPYSTFESLLAHLWRVITKARGLSGFESTQIRISVNGRTRLNPKVPNEYF
GNLVLWAFPTTKVKELLREPLPHATKLIHDAIAKVNNNYFRSFIDFANTKAKEEDLVPTA
DMNKYILCPNIEVDSWLRFPFYDLDFGTGCPYMFMPSYFPTEGMMFLIPSFVGDGSID
VFVPLFEDKLPLFKKICYSLDLLED

>Solyc11g067270
MASSTIISRKMIKLLSPTPSSLRCHKLSFMDHINFPLHSPYAFFYPKIPQNYSNKISQVLE
NSLSKVLSFYYPLAGKINNNYTYVDCNDTGAEYLNVRIDCPMSQILNHPYNDVVDVVFP
QDLPWSSSSLTRSPLVVQLSHFDCGGVAVSACTSHTIFDGYCLSKFINDWASTARNME
FKPSPQFNASTFFPLPSETNLSSTLPATRPSQRHVSRMYNFSSSNLTRLKDIVTKESHV
KNPTRVEVASALVHKCGVTMSMESSGMFKPTLMSHAMNLRPPIPLNTMGNATCIILTT
AMTEDEVKLPNFVAKLQKDKQQLRDKLKDMKEDRMPLYTLELGKNAMNIIEKDTHDVY
LCSGMTNTGLHKIDFGWGEPVRVTLATHPNKNNFIFMDEQSGDGLNVLITLTKDDMLK
FQSNKELLEFASPVVESTK

>Solyc11g067290
MASSTILSRKMIKLLSPTPSSLRHHKLSFMDCINLPQYSPFAFLYPKPKNHTKTQISQILE
NSLSKVLSSYYPFAGRIKDNNTYVDCNDIGVEYLNVRINHSMSDILNSRCNDVADIVYP
KDLPWSSSVNRSPLVIQLSHFDCGGIGLGVCLSHKIVDGYCIAKFISDWANTARDMDFK
PSIKFNASTFFPLIEGAPNIMSINSSPQSQRYVSRAYNFSSSNLKRLKRSITGVQNPTRV
EVATSLIHKCGAIASMKNLGSFKPSLISQVINLRPLIPLDTMGNATCIYSIIATTENEIELPN
YVAQMQNVKQQIRNELKNLDTDKIVPYTLEKVRGIVDIMEKDIFDIYLSTSVHNFGLYSK
ANFGWGKPIKVSNTKYPTKKSIMLFDDPSEEGIDAQITLTEDEMAIFQEDKELLEFASPM
VQSTK

>Solyc11g067330
MASSTILSRKFIKPSPPTPSSHRHYNLSFRDQTANNLYLPIAALYSKPENHTITQISNILE
NSLSKILFFYYPFGGRIKDNKYVDCNDIGAEYFNVHINCQMSEILSNPYNDAIEIVFPQNL
AWGNSLSEERSSLLVVQLSHFDCGGVGISICLSHKVADGYSGCKFLSDWVSMARDDH
KLNFQLSCQFDGASFFPPIDNPPPMPNVVPDPERCVSRMYNLSSSTLLKLKDIVVSTNP
QIQNPSRIEVATALFHKCGVDVSMAKSGVFRRTVLFHVMNLRPPIPFNTIGNATCYFST
RAMSIDKTTLPNYVGELQKAKQQIRYELKDMNTKELALHAIEKIKEIVNIAKDDCFDMYF
CTSLCTFGSRKIDFGWGSPLRVTHVKDPMKNKFIFMDDPSGEGINVLITLTEADMLLFE
SNKELLEFASPVVQSLE

>Solyc11g067340
MASSTILSRKIITPSPPTPSSHRHCNLSFRDQTANTLYLPIAALYSKPENHTITQISNILEN
SLSKILYFYYPFGGRIKDNKYVDCNDIGAEYFNVHINCQMSEILSNPYNDAIEIVFPQNM
AWGNSLSEERSSLLVVQLSHFDCGGVGISICLSHKVADGYSAFKFLGDWISMARDDHK
LNFQPSPQFDGALFLPPIDNPPPMPNVVPDPQQCVSRMYNLSSSTLSKLKDSIVSTNP
QIQNPSRIEVAMALIHKCGVDVSMSKSGVFRPTMLYQVMNLRPPIPLNTMGNATCLFS
TIAMSIDKTTLPSYVGELQKAKQQLRHKLKQMDTNQLASHAIEKLKEIVNIANEDVFDIYF
CTSLCTFGSHKIDFGWGSPLRVTQVKHPTKNKFMFLDDPSGEGINVLITLTEADMLLFQ
NNKELLEFASPVVQSLE

>Solyc11g067350
MAKSGAFRPTVLFHVMNLRPPIPFNTIGNATCYFSTRAMSIDKTTLPNYVGELQKAKQQ
IRYELKDMNTKELALHAIEKIKEIVNIANEDVFDIYFCTSLCTFGSHKIDFGWGSPLRVTQ

VKHPTKNKFMFLDDPSGEGINVLITLTEADMLLFQNNKELLEFASPVVESSLLGSTLSLN
VELSDVKSKL

>Solyc11g069680
MSSFSRLLSTISKKVVKPFSPTPSTQKIHKLSLLDQCMGNFYMPLVLFYPKHQLEQGPK
QLSKLLETSFSKVLTYHQPWAGSLRDNATIHCDGTGAEFFEVEVNCPMNQIVHRPDLT
FPPGLPWKNVPHVNDGGRLSVAQLSHFDCGGIAISVCMSHKVGDARSAFSFLKDWAT
LTREQSNDQLSCPSNSNDQLSCPSYYVQDSLMPSLPDGPLKFPVVVEPNGEESIEFEK
RFFLSESNIRALKALIVDDPSSIVQNPTTTEVVSAIVYKCAAIAGANTSNGNNDSSSQMV
FVSDLRKTIPPSIKSTSTIGNILTTFSTPTYNLEDLRLPKLVADIRKSKHELSTRDNFNENR
WVSEMIEYANKINTGTEYELSYRRESSSHDVYRCSSVCNIPFQDLDFGWGRPTRASIA
STPFNNMIYLMNTQDQNRGIEVFINLDQQQMSIFEQDKEFLQFASPVGDNNFHEDKEK
LCCL

>Solyc11g071470
MNVKIESSKIIKPLYEGIPPSTTIHIPFNVFDNVTFDTLMALIYAYRPPTPPTSTIETGLRKT
LSIYREWAGRIGEDEHGNRGVFLNDEGVRFIEASVDASLDEVLPLKPSPSMLSLHPSLK
DVVELIQVQVTRFTCGSVVVGFTGHHMIADGHAASNFFVAWGQACRGMEITPIPMND
RAIFRPRNPPLVEYNHVGAEFVSKLVNKELVKINNDQDKEKNIIVHKVHFTLEFLGKLKA
HASFMNGKAKTYSTFESLIAHLWRVITNSRNLNASQNTQIRISVDGRRRITPRVPDEFF
GNMVLWAFPTSKVKDLLDEPLHYATKIIHEAITKVDDKYFKSFIDFANDEKVMTRQDLIP
SANMKNDSLCPNLEVDSWLRFPFYDLDFGTGCPFLFMPSYYPIEGMMFLVPSFIGDGS
IDAFIPLYEHNLTNFKKICYSLDLKAK

>Solyc11g071480
MNVKIDSSKIIKPLYEGTPPSTTTHIPFNIFDNVTFDALMALIYAYRPPTPPTSTIEIGLRKT
LSIYREWAGRIGEDEHGNRGVFLNDEGVRFIEASVDTSLDEVLPLKPSPSVLSLHPSLK
DVVELIQVQVTRFTCGSVVVGFTGHHIIADGHAASNFFVAWGQACRGMEITPLPVNDR
TIFRPRDPPLVEYNHVGAEFVSKLGYKELVKVNNDEHKEKNIIVHKVHFTLEYLGKLKAH
ASFMNENAKTYSTFESLIAHLWRVITKSRDLNAFQNTRIRISVDGRRRIIPRVPDEFFGNI
VLWAFPTSKVKDLLDEPLHYATKIIHEAISKVDDKYFKSFIDFANDEKVMTRQDLIPSAN
MNNESLCPNLEVDSWLRFPFYDLDFGTGCPFVFMPSYYPIEGMMFLVPSFIGDGSIDA
FIPLYEHNLTNFNKICYSLDLKAK

>Solyc12g005430
MEIGFVNENDCIKVEILCKKLIKPSSPTPSQNQRYKLSFFDQIAEREHIPVVLFYPYNNIN
SHTIDERLEKSLSDVLTHVYPAAGRYDDNAECSILCLDQGVSYTKAKVNCKLGNFLEKT
RKDLSVATLFGPHENKNMDQNNFMVSPIVIIQVTKFECGGLALSFSVSHPAMDGFTGL
QFLFGWGKVCRLGTPIDKIHFLSFNLGNIFPTRDTSALFNSADVVNREENIVVKRFVVR
EAALSRLKKQCIDESGGALTFQPSRVEIVTAILWRAFIRASAARNGFVRPSLMDFPLNL
RSKSSLPQVKTSMGNFRIDVPIKFIPGETKMELHNFITLIRNTMNKVVASFAKPSPDEIVS
TLVNIYNQSFTSPEWGGNNEIDKVACSSLCKFPLQDIDFGLGKPSLVYFGLKDMEIFWL
YDTDCHTSEIGVQLDLKESTMQLFECDNDIKALMFIRDAKL

>Solyc12g005440
MEIKILCTKLIKPFLPTPPHLQHYKLSFFDQISEKEHVSMVFFFHNYNNIDMDERLEQSL
SKILTHVYPAGGRYNEKDKYCSILCVDQGVFYTKAKTNGTLDNFLNKARNDLGHAALF
SPHVNKNIDETNFMVSPIVTIQVTEFECGGVAISISTSHPAMDGFSDFQFISEWAKVCRI
GTPIDKINILSFNMGDIFPTRDITGIFKSTPTPIIQQDIVVKRIVIHEDVMSRLRKKCTFSTF
QPSRVEIITAILWRAFIRATAIINGYLRPSLLDFPMNMRSKITFLPQVKNSYGNFMIGVPV
KFIPGENKMELHDFIMLIRNAVNKIVASCKKANSPDEIVATLVDSYNVSFRSPEWGGND
EVDKVMCTSICKFPVHDSDFGLGKPNLIFFGMKDTQMFWLYDIGPEIVVQVDLKERCM
QLFDREDDIKDLIFIRDAKL

>Solyc12g006330
MSASRLVSLSRKIIKPSSPTPLSHRIHKLSLMDQMGTHSYMAALIFYPKQNTTTSMPEP
TKISRVLEKSLSKVLTSYYPFAGQVRDNSFVECNDIGADLSQVRIDCPMSSIFDHPRTYI
DNLVLPFDPWFPSTDSLVAAKLCHFECGGVALGVCLSHKVSDGYSLGKFLKDWSMVA
RDSEAKLSLLFNGSSIFKPSNSSSFQVVADPPLYKNESKRFHFSASNLKSLKSLISSADS
ATQICPTTVEAITAFIYRCVSTPQSLLIQAVDQRGTSNDALVPADLTGNAILPFVVSATNK
EEMNLERLVSELRKGKEKIQDMLKYIESEEFLCSKVSDIARELNKRTSNNDIPMYRFSSL
RRFPSNDINFGWGRPRQVDISTFPINMFILMDNQNGDGVEVIASLQDGELSALERNDD
FLQFASPCLGF

>Solyc12g010980
MKDSMQVTILSKNLIKPSLPTPKHLKYHKLSFFDQVADVAHLPLVLFYPHCKNNSKHEE
LEESLSRILSHVYPLAGRFAEDDESLILCLDQGVTYIKAKVNCTLDDLLQQTKKDLDLAL
SFWPQGTMDVDDSNLFVTPLMVVQVTTFECGGLALAISIAHPVMDGCTTFKILYEWTK
VCKFGTPSKEISFMNFNVGALFPYKHDLSTLLEPPVDEGKRKDSKLIARKFVFEKDAISR
LREKFDSISESLGFKPSRVEMITALLWRSLIRSTKSAKLKRSVMSFPLNLRGKVADFPEI
TDSFGNLIIEVPIKFEHDDETKIESLHQIVKLIKESVKVINNKCVKATPDEIISLVIDLYKDSY
SGLEWGGDDEVMNFTSSSLSRFPIQKTDFGWGKPSLMHFGSRHNQVLWLYDTECET
GIVVQMDLEKKHMDKLVCDQDIIDFAKF

>Solyc12g044660
MASVIEQCQVVSPPRYASEVTFQLTCFDHTWLAFGCTQQILFYNHHFVQIIVPSLKHSF
SLALKHYTILSGNLVSPLINSSGYPELRYKTGDFVFVTFSETTATDFNYLISNHPRYAKD
FYPFIPQLAEPKNLIPFYDRSILKDPHEQEMAIWDVMKTFKVEMRDIIVIPDVDKVRGTFII
GHNEITKLKNFILSRR

>Solyc12g087980
MDHVEKLISDVKLSSVVPGRITGDDKLQHEFTNMDLIMKLHYIKALYFFKNDVVEGLHIH
DLKLPMFNLLELYYPISGRIRRYDGGDGGGGGGRPFMKCNDSGVRVVEAKCKNKTID
EWLAMNDNDHDEELVYDQLLGPDLGFSPLVFIQFTWFKCGGMSIGLSWAHILGDSFSA
SNFLNIWAKIMVGQQISPQFLHKSTKTNKLINNNNNNNPILSTITKFPFSLKRVDPVGDH
WKITNNIKMQSHSFHITQNQLNQLVSNVCGTYNKVKPFDVICATLWKMLAKVRGEYSS
EPAIVTIIRGDHDSETTEAVSSNNQVTISTVEANDIKVSDVDTSELTELIGEKTVDETRIVE

ELMKKENGVSDFIVYGANLTFVNLEEAMIYDLEVRGKKPIFASYNISGVGDEGVILVLPR
LEGGRIVNLVLPKKQIEGLKNKMREELGIF

>Sl FdAT1 (Solyc12g088170)
MSQITTQNLNGTCIQIEILNEKLIKPSLPTPNHLNSYKLSFFDQIAPNFAVPLLYFYPPVPP
ENSHLQRVEEVHKQLQNSLSEVLTKFYPLAGRLSEDGTSIECHDQGVIYLEAKVNCQL
NEFLDKAYKDSDLVKIFVPPIRIRLAELPNRPMMAIQATMFEHGGLALAVQIVHTTGDGF
SGCAITDEWAKVSRMEKGNVRNLQFRSDLVEVFPPRDNILEMIKKGRPRGYEMKIATRI
FMFDEIAISKLKENVNKFMSYSSRVEVVTALIWRSLMRVVRLRHGHNRPSMLQFAINLR
GRGSPRVVGEDQNFFGNFYLDIPIKYVSSRSNQDPELHEIVTLIRNAKNKILSEIANASS
EEIFSILIESLNQIREGYNDDEIDLYPTSSLCKFPLNESDFGWAKPIWVSRVNVPFQMFF
LMDSKNGIEARVCLNEEDMMKLEKDVDIVEFSYVPK

>Solyc12g096250
MEEIQIVSTCVVRASSNSNNGITHTSQNIEMTPWDLQFLLVETIQKGLLFKKPTPQQQN
NLIKSLNSLSLVDHLKASLSRTLDFFPPLAGRFSTTKNPNDNTITSFSITCNNSGAEFTH
AIAPELTVKEILESCYVPTIVHSFFPLNKVRNVQCVTKPLLGVQVTELVDGYFIGCTMSH
SLGDGTCFWHFFNSWSEITRGFELISRFPTLERWFPQNVNPPIYFPLELDDEKLDVCM
EMPIVKERVFHLSKENVSKLKAKANYEMDTKSISSLQAFLAHLWRCVTRCNRVNANEE
VILNIVIGTRTRLDPPLPEEYWGNAAIIKPIKVKAGELLENGLGYAALLINKVVASQNYEE
VMDSYRRWVENPVIVSKKSLFVANRLSISSSPKFNVYSCDFGWGKPVGVRSGMANKD
NGKVTLFRGVEEGSVDIEVCLMTSTLLAMENDEEFMEFITV

>Solyc12g096770
MEIEIISTKFIKPSSPTPNHLQTYKLSFFDQVSDETHLPLVFFYPPTNNINFSSHHEEQLE
QSLSRILTHVYPISGRFNEDINSISCQDQGVKFIKAKMNSKLNEFLDKAHKDVNLSLLCW
PQDSWNVDPSNLFTMPLVIIQITEFECGGLALSMSHMHMTMDGYSTFSFINEWSKVCR
HKIPLEKIDFMSFDLANVFPTRDLSKLLLPRIPPVDRVECKLVARRLYINEDSISRLREKV
SGDLCKFKPSRVEMIMAILWRAVIRASEKKHGYLRRSLMNIPINLRTRLISLPQVEKSFG
NLGVDAPIKFIPEENKMELHEFVTLIHNAVKETITTCDKTSPEDIVSAVSNIYNESFLAQD
WGGNDEVDRIISSSLCKFPIQEADFGWGKPCLMHFGSRHGQVCWLYDAECGNGICV
QVDLKEDNMNLFECDNDIKDFFQF

>Solyc12g096790
MAFQKENMQVEIISTKFIKPSSPTPNHLQIYKLCFFDQVTDETHLPLVLFYPPTNNINLSS
HHEEQLEQSLSRILTHVYPISGRFNEDINSISCQDQGVKFIKAKMNSKLNEFLDKAHKDV
NLSLLCWPQDSWNVDPSNLFAMPLVIIQITEFECGGLALSLSHVHMAMDGYSTFSFINE
WSKVCRLEIPVEKIDFMSFDLANVFPTRDLSKLLLPRVPTEDRVESKLVAKRLYINEDSI
SRLREKVGGDLCKFKPSRVEMITALLLRALIRASEKKHGYLRRSLMNIPINLRTRLTCLP
QVEKSFGNLGVDAPIKFIPGENKMELHEFVTLIHNTVKETIATCDKTSPEDIVFAVSNIYN
KSFLAQDWGGSDEVDKYTSSSLCKFPIQEADFGWGKPCLMHFGSRHDQCCWLYDAE
CGNGICVQVDLKEDHMHLFECDNDIKYFFSF

>Solyc12g096800
MELNLEFISTKLIKPSIPTPPHLKNYKLSFFDQLAEREHMPLLLFYPYGNNNDIGDDLFD
QKLEKSLSRILSHVYPAAGRLSRDRFSIDCLDQGVTFTKAKVNCQFNDFIDQVQKDLNL
ALFFFPRDIQDLKDVDFDSTPPMVVQVTKFECGGIAMSISASHLVMDGFSNFKFVYEW
AKVCKFEIPDDEIDFMSFDFGEILPARDLSRIFPNRVHPVESEERFIANRFFITEQTISSLR
DKLTGAIDSGELCFKPSRVEIITAILWRALIRVSEAKHGYLRRSLVFFPVNLRGRISLPLK
ENAFGNYVMDAPIMFVPEKNKMELHDFVTLIRNSVQKAIDACAIGTADDIIANVADSYKE
IFASKEWGTDNDEVDKCIISSLCKFPMKDADFGRGKPSLMHFGLRNFHSCWMYDAEC
GSICVQVDLKDSYMSLFECQSDIKAFTNVLGNQERIQLQPLL

>Sl ACT2 (Solyc11g071480)
MNVKIDSSKIIKPLYEGTPPSTTTHIPFNIFDNVTFDALMALIYAYRPPTPPTSTIEIGLRKT
LSIYREWAGRIGEDEHGNRGVFLNDEGVRFIEASVDTSLDEVLPLKPSPSVLSLHPSLK
DVVELIQVQVTRFTCGSVVVGFTGHHIIADGHAASNFFVAWGQACRGMEITPLPVNDR
TIFRPRDPPLVEYNHVGAEFVSKLGYKELVKVNNDEHKEKNIIVHKVHFTLEYLGKLKAH
ASFMNENAKTYSTFESLIAHLWRVITKSRDLNAFQNTRIRISVDGRRRIIPRVPDEFFGNI
VLWAFPTSKVKDLLDEPLHYATKIIHEAISKVDDKYFKSFIDFANDEKVMTRQDLIPSAN
MNNESLCPNLEVDSWLRFPFYDLDFGTGCPFVFMPSYYPIEGMMFLVPSFIGDGSIDA
FIPLYEHNLTNFNKICYSLDLKAK

>Sl AAT1 (Solyc08g005770)
MANILPISINYHKPKLVVPSSVTSHETKRLSEIDDQGFIRLQIPILMFYKYNSSMKGKDLA
KIIKDGLSKTLVFYYPLAGRLIEGPNKKLMVNCNGEGVLFIEGDANIELEKLGESIKPPCP
YLDLLLHNVHGSDGIIGSPLLLIQVTRFTCGGFAVGFRFNHTMMDAYGFKMFLNALSELI
QGASTPSILPVWERHLLSARSSPSITCIHHEFDEEIESKIAWESMEDKLIQQSFFFGNEE
MEVIKNQVPPNYECTKFELLMAFLWKCRTIALNLHSDEIVRLTYVINIRGKKSLNIELPIG
YYGNAFITPVVVSKAGLLCSNPVTYAVELIKKVKDHINEEYIKSLIDLMVTKGRPELTKS
WNFLVSDNRYIGFDEFDFGWGNPIFGGILKAISFTSFGVSVKNDKGEKGVLIAISLPPLA
MKKLQDIYNMTFRVIISNI

>Sl HQT (Solyc07g005760)
MGSEKMMKINIKESTLVKPSKPTPTKRIWSSNLDLIVGRIHLLTVYFYKPNGSSNFFDNK
VIKEALSNVLVSFYPMAGRLGRDEQGRIEVNCNGEGVLFVEAESDSCVDDFGDFTPSL
ELRKLIPSVETSGDISTFPLVIFQITRFKCGGVALGGGVFHTLSDGLSSIHFINTWSDIAR
GLSVAVPPFIDRTLLRARDPPTSSFEHVEYHPPPTLNSSKNRESSTTTMLKFSSEQLGL
LKSKSKNEGSTYEILAAHIWRCTCKARGLPEDQLTKLHVATDGRSRLCPPLPPGYLGN
VVFTATPIAKSCELQSEPLTNSVKRIHNELIKMDDNYLRSALDYLELQPDLSTLIRGPAYF
ASPNLNINSWTRLPVHECDFGWGRPIHMGPACILYEGTIYIIPSPNSKDRNLRLAVCLDA
GHMSLFEKYLYEL

>Sl ASAT4 (Solyc01g105580)
MNCYIEIQSRKMVKPSAPTPDNLRRLKLSLFDQMDIGAYVPIVFNYLPNSTSSYDHDDK
LEKSLSETLTKFYPFAGRFRKGIDPFSIDCNDEGIEYVRTKVNADDLAQYLRGQAHNDI
ESSLIDLLPVMHRLPSSPLFGVQVNVFNNGGVTIGIQILHMVSDAFTLVKFVNEWAHTTL
TGTMPLDNPGFGQLPWLFPARALPFPLPDFNTTTAPNYKNVTKRFLFDALAIENLRNTI
KANDMMMKQPSRVVVVMSLIWKVLTHISSAKNNGNSRDSSLVFVVNLRGKLSCTAPS
LEHVVGNCVIPATANKEGDEARRKDDELNDFVKLVRNTIRDTCEAIGKAESVDDISSLA
FNNLTKCIEKILHGDEMDFYSCSSWCGFPWYEADFGWGKPFWVSSVSFGHHGVTNL
MDTKDGDGIQVTICLKENDMIEFERDPHILSSTSKLAFHSLG

>Sl ASAT3 (Solyc11g067270)
MASSTIISRKMIKLLSPTPSSLRCHKLSFMDHINFPLHSPYAFFYPKIPQNYSNKISQVLE
NSLSKVLSFYYPLAGKINNNYTYVDCNDTGAEYLNVRIDCPMSQILNHPYNDVVDVVFP
QDLPWSSSSLTRSPLVVQLSHFDCGGVAVSACTSHTIFDGYCLSKFINDWASTARNME
FKPSPQFNASTFFPLPSETNLSSTLPATRPSQRHVSRMYNFSSSNLTRLKDIVTKESHV
KNPTRVEVASALVHKCGVTMSMESSGMFKPTLMSHAMNLRPPIPLNTMGNATCIILTT
AMTEDEVKLPNFVAKLQKDKQQLRDKLKDMKEDRMPLYTLELGKNAMNIIEKDTHDVY
LCSGMTNTGLHKIDFGWGEPVRVTLATHPNKNNFIFMDEQSGDGLNVLITLTKDDMLK
FQSNKELLEFASPVVESTK

>Sl ASAT1 (Solyc12g006330)
MSASRLVSLSRKIIKPSSPTPLSHRIHKLSLMDQMGTHSYMAALIFYPKQNTTTSMPEP
TKISRVLEKSLSKVLTSYYPFAGQVRDNSFVECNDIGADLSQVRIDCPMSSIFDHPRTYI
DNLVLPFDPWFPSTDSLVAAKLCHFECGGVALGVCLSHKVSDGYSLGKFLKDWSMVA
RDSEAKLSLLFNGSSIFKPSNSSSFQVVADPPLYKNESKRFHFSASNLKSLKSLISSADS
ATQICPTTVEAITAFIYRCVSTPQSLLIQAVDQRGTSNDALVPADLTGNAILPFVVSATNK
EEMNLERLVSELRKGKEKIQDMLKYIESEEFLCSKVSDIARELNKRTSNNDIPMYRFSSL
RRFPSNDINFGWGRPRQVDISTFPINMFILMDNQNGDGVEVIASLQDGELSALERNDD
FLQFASPCLGF

>Sl GAME36 (Solyc08g075210)
MTASSFVSMAEKIIKPHSPTPFSVKRYNLCLMDEIMVPVYMPIVAFYPNPSKTPEQVSNI
LEDSLSKVLSSYYPFAGTLGSDNATFVDCNDRGAKSIQVRYDCPMSEIVNLPDTGPEY
LPFAKGTPWSSTPEEQSLLVVQLSHFNCGGLGISARLSHKIADGCTLANFISDWASVAR
DDNANIPSPQLIGSSIFPPFTEMRIHTDTNVDYEFYNLPVCKKRYLFSNAKLEMLKTQVE
SETGVQNPTRIEVLSALIYKCAVTANSSSFRPSSLSLPVNLRPILNPPLETRTVGNIISFIK
VETTSEDEMTIGRVVREIRKGKDELKQEGGVKKEKLVSLWSEWIHSIDLYRSSSVCNY
PLNNLDFGWGKPNRVAIPVFGVANTCMFMDNLSGDGIEVIIALPEKDATQFENSKELLH
FASPVTNL

>Sp GAME36
MTASSFVSMAEKIIKPHSPTPFSLKRYNLCLTDEIMVPVYMPIVAFYPNPSKTPEQVSNI
LEDSLSKVLSSYYPFAGTLGSDNATFVDCNDRGAKSIQVRYDCPMSEIVNLPDTGPEY

LPFAKGTPWSSTPEEQSLLVVQLSHFNCGGLGISARLSHKIADGCTLANFISDWASVAR
DDNANIPSPQLIGSSIFPPFTEMRIHTDTNVDYEFYNLPVCKKRYLFSNAKLEMLKTQVE
SETGVQNPTRIEVLSALIYKCAVTANSSSFRPSSLSLPVNLRPILNPPLETRTVGNIISFIK
VETTSEDEMTIGRVVREIRKGKDELKQEGGVKKEKLVSLWSEWIHSIDLYRSSSVCNY
PLNNLDFGWGKPNRVAIPVFGVANTCMFMDNLSGDGIEVIIALPEKDATQFENSRELLH
FASPVTNL

>Sp GAME36-like
MAAASRLVSFAEKIIKPNRATPLSLGRYNLSINDQIMVPFYLSIAAFYPNPSKTPEQVSNI
LQNSLSKVLSSYYPFAGTLRDNTFVDCNDRGAKFMNVRYDCPMSEIANLPDTGPEYLP
FAKGIHDSLLLGDEDLLVVQLSHFNCGGLAIGTSISHKIADGCTTSNFISDWASIARDDE
EANIIPSPQMIGSSIFPPSTNLPSTDIYTNTIVNVPIENIKRRYLFSNSKLEMLKSQVTSET
GLQNPSRVDVLSALIYKCAVTAARAQANSFKPSMLTLAVNLRPILDPPVATRAIGNMVS
FIKVETTSVDEITIARVVRELRKAKDEFKKEDHVNANKLVALHSENLPISNEFETYRSHS
MCNFPLNNLDFGWGKPNKVTIPLIGVGFCFFLLDSPSGDGIEAIVAVPETYVTQFENNK
ELLQFATPMN

>Sm GAME36
MAASRLLSFDEKIMKPSCPTPLSLRRYNLCRIDQFIPHFHMAMAAFYPFPSKKPQEISDI
LEKSLSKVLSSYYPFAGRLKDNIFVDCNDQGAKFINVRYNCPMSEVVNLPDTSPECLPF
PKGITRIQDEENLLIIQLSHFNCGGLALSVNLSHKIADGCTLCNFFSDWASIARDHNANIP
SPQMIGSSIFPPSTDTIQRDTTVDEIDNTPLENKRYVFSNSKLEMLKAQVASETGIQNPT
RVEVLSALIYKCATAGPRVKPSVLILPVNLRPIFNPPIPTRVIGNIFSLIRVPTMMDDEITV
AGVVRELRKAKDELKKEDNVKNNNMISISSELSSMANEVELHYSTSLCNYPLNNLDFG
WGKPSRVTMQLRGHKNGFLFMDNQRGDGIERDPILVTALHCILVPYAPSWFCNNAHS
ALTRLFNSVTPC

>Sl ASAT2 (Solyc04g012020)
MSSSVSRLVSSVCKKIIKPYSPTPISLRCPKLSYLDQMVGGIYIPLALFYPKLSNTWSNK
PNNVVSQHLEKSLSKVLTNYYPFAGKLNDNISIDCNDNGVEFFVTEINCPMSEIFNHPY
FEKHNLVYPTEVINNQYTYEGSLAVFQLTHFNCGGIAISMCLSHKVGDGYTFGNFMNH
WATIARNPLSSEIYPISPKFDGSFYFPPAKDEDSSNVSNNNIVPQREECVSKGFSISSSK
LTALKARVINDSEVQNPSDTEVVSAFLYQRAMATKKLVNSDSIRPSLLHQAVNLRPPLP
KHTMGNICSLFSILTKEEKEMDLARVVSKLRKEKEEVKQKYKNAKIEELLPITLEQHRKA
NDLLVNNSCYDLYRFSSLITFPSYEVDFGWGKPEKVISPISTSNPPIKNMFFLMADKNR
DGVNLTCSMKKQDMLAFERDEELLRFASPTS

>St GAME36
MSASRLLSFGEKIIKPNCPTPFSLGRYNLSVIDQIMFPVYMPIAAFYPFPSKTPQQVSNII
EKSLSKVLSSYYPFAGTLRDNTFVDCNDRGAKFMNVRYDCPMSEIVNLPDIGPEYLPF
PKGVPWGRTSDEKSLLFVQLSHFNCGGLAISASLSHKVADGCTLCNFFSDWASIACDH
NANIPSPQMIGLPILSSFTNMPSTESFTDTVVDEIDNQPLSRKRYVFFNSKLEKLKTQVE
SETGVQNPTRFEVLSALFYKCAATAARANSSSFKPSMLLLPVNLRPILDPPLPTRAIGNI
LSMIKVTAMSEDEMTIARVVREIRKAKDELKKEDHVKESKLVSLWSELSSMGNEFELYR

STSVCNYPLNNLDFGWGKPNMVTIPITGIGNSFIFMDNQSGDGIEAIVALPEKNVTRFE
NSKELLEFASPIANLN

>*S. pimpinellifolium* LA1589 (GAME36)
MTASSFVSMAEKIIKPHSPTPFSVKRYNLCLMDEIMVPVYMPIVAFYPNPSKTPEQVSNI
LEDSLSKVLSSYYPFAGTLGSDNATFVDCNDRGAKSIQVRYDCPMSEIVNLPDTGPEY
LPFAKGTPWSSTPEEQSLLVVQLSHFNCGGLGISARLSHKIADGCTLANFISDWASVAR
DDNANIPSPQLIGSSIFPPFTEMRIHTDTNVDYEFYNLPVCKKRYLFSNAKLEMLKTQVE
SETGVQNPTRIEVLSALIYKCAVTANSSSFRPSSLSLPVNLRPILNPPLETRTVGNIISFIK
VETTSEDEMTIGRVVREIRKGKDELKQEGGVKKEKLVSLWSEWIHSIDLYRSSSVCNY
PLNNLDFGWGKPNRVAIPVFGVANTCMFMDNLSGDGIEVIIALPEKDATQFENSKELLH
FASPVTNL

>*S. habrochaites* LA1777 (GAME36)
MTASSFVSMAEKIIKPHSPTPFSVKRYNLCLMDEIMVPVYMPIVAFYPNPSKTPEQVSNI
LEDSLSKLLSSYYPFAGTLGSDNATFVDCNDRGAKFIQVRYDCPMSEIVNLPDTGPEYL
PFAKGTPWSSTPDEQSLLLVQLSHFNCGGLGISARLSHKIADGCTLANFISDWASVAR
DDNANIPSPQLIGSSIFPPFTEMRIHTDTNVDYEFYNLPVCKKRYLFSNAKLEMLKTQVE
SETGVQNPTRIEVLSALIYKCAVTLDEANSSSFKPSSLSLPVNLRPILNPPLETRTIGNIIS
FIKVDTTSEDEMTIGRVVREIRKGKDELKQEGDVKKEKLVSLWSEWIHSIELYRSSSVC
NYPLNNLDFGWGKPNRVTIPVFGVANTCMFMDNLSGDGIEVIIVLPEKDVTQFENSKEL
LQFASPVTNL
