# Supplementary material for: A BAHD-type acyltransferase concludes the biosynthetic pathway of non-bitter glycoalkaloids in ripe tomato fruit
Source: Nat Commun. 2023 Jul 27;14:4540. doi: 10.1038/s41467-023-40092-5 (PMC10374582; doi:10.1038/s41467-023-40092-5)
Supplement: Supplementary file 7 — Reporting Summary [file 41467_2023_40092_MOESM7_ESM.pdf]

nature portfolio

Corresponding author(s): Prashant Sonawane
Asaph Aharoni

Last updated by author(s): 2023/06/08

Reporting Summary

Nature Portfolio wishes to improve the reproducibility of the work that we publish. This form provides structure for consistency and transparency in reporting. For further information on Nature Portfolio policies, see our Editorial Policies and the Editorial Policy Checklist.

Statistics

For all statistical analyses, confirm that the following items are present in the figure legend, table legend, main text, or Methods section.

n/a	Confirmed	
☐	☒	The exact sample size (*n*) for each experimental group/condition, given as a discrete number and unit of measurement
☐	☒	A statement on whether measurements were taken from distinct samples or whether the same sample was measured repeatedly
☐	☒	The statistical test(s) used AND whether they are one- or two-sided<br>*Only common tests should be described solely by name; describe more complex techniques in the Methods section.*
☒	☐	A description of all covariates tested
☒	☐	A description of any assumptions or corrections, such as tests of normality and adjustment for multiple comparisons
☐	☒	A full description of the statistical parameters including central tendency (e.g. means) or other basic estimates (e.g. regression coefficient) AND variation (e.g. standard deviation) or associated estimates of uncertainty (e.g. confidence intervals)
☐	☒	For null hypothesis testing, the test statistic (e.g. *F*, *t*, *r*) with confidence intervals, effect sizes, degrees of freedom and *P* value noted<br>*Give P values as exact values whenever suitable.*
☒	☐	For Bayesian analysis, information on the choice of priors and Markov chain Monte Carlo settings
☒	☐	For hierarchical and complex designs, identification of the appropriate level for tests and full reporting of outcomes
☒	☐	Estimates of effect sizes (e.g. Cohen's *d*, Pearson's *r*), indicating how they were calculated

Our web collection on statistics for biologists contains articles on many of the points above.

Software and code

Policy information about availability of computer code

Data collection	All presented data have been acquired using existing and routinely used softwares. These were mentioned in the respective parts of manuscript. Except wild tomato accession analysis, LC-MS data presented in this study was collected and analyzed using UPLC-TQ-MS (Waters) and UPLC/qTOF (Waters Acquity) instruments. For wild tomato accession, LC-MS data was collected and analyzed using Thermo Scientific UltiMate 3000 ultra-high performance liquid chromatography (UHPLC) system coupled to an Impact II UHR-Q-ToF (Ultra-High Resolution Quadrupole-Time-of-Flight) mass spectrometer (Bruker Daltonics).
Data analysis	TargetLynx program (Waters) and Bruker Compass Data Analysis (Version 5.3) were used for LC-MS data analysis. Microsoft Excel 2016 and GraphPad Prism 8 softwares were used for regular statistical analysis and enzyme kinetic analysis.

For manuscripts utilizing custom algorithms or software that are central to the research but not yet described in published literature, software must be made available to editors and reviewers. We strongly encourage code deposition in a community repository (e.g. GitHub). See the Nature Portfolio guidelines for submitting code & software for further information.

Data

Policy information about availability of data

All manuscripts must include a data availability statement. This statement should provide the following information, where applicable:

- Accession codes, unique identifiers, or web links for publicly available datasets
- A description of any restrictions on data availability
- For clinical datasets or third party data, please ensure that the statement adheres to our policy

Data supporting the findings of this work are available within the paper and its Supplementary Information files. Publicly available RNA-seq data used in our manuscript was retrieved from NCBI Sequence Read Archive with BioProject ID PRJNA307656 [https://www.ncbi.nlm.nih.gov/bioproject/PRJNA307656/] and PRJNA798612 [https://www.ncbi.nlm.nih.gov/bioproject/?term=PRJNA798612]. Source data are provided with this paper. Correspondence and requests for materials should be addressed to P.D.S. or A.A.

Human research participants

Policy information about studies involving human research participants and Sex and Gender in Research.

Reporting on sex and gender	Not applicable to our study
Population characteristics	Not applicable to our study
Recruitment	Not applicable to our study
Ethics oversight	Not applicable to our study

Note that full information on the approval of the study protocol must also be provided in the manuscript.

Field-specific reporting

Please select the one below that is the best fit for your research. If you are not sure, read the appropriate sections before making your selection.

[x] Life sciences [ ] Behavioural & social sciences [ ] Ecological, evolutionary & environmental sciences

For a reference copy of the document with all sections, see nature.com/documents/nr-reporting-summary-flat.pdf

Life sciences study design

All studies must disclose on these points even when the disclosure is negative.

Sample size	Details of biological replicates related to various experiments are provided in the manuscript, wherever necessary. In case of game36 crispr lines, we presented sample size data from T1 generation with biological replicates only. For example, for game36 crispr line #41, n=2 meaning these are two biological replicates collected from two independent positive plants (T1 generation).
Data exclusions	No data was excluded in our analysis
Replication	Details of biological replicates used in various experiments are provided in methods section as well as in Main Figures and Supplementary Figures legends, wherever necessary.
Randomization	Biological replicates are collected from independent genotypes. Sample size was mentioned for each experiment in figure legends.
Blinding	Blinding was not relevant for our study

Reporting for specific materials, systems and methods

We require information from authors about some types of materials, experimental systems and methods used in many studies. Here, indicate whether each material, system or method listed is relevant to your study. If you are not sure if a list item applies to your research, read the appropriate section before selecting a response.

Materials & experimental systems

n/a	Involved in the study
☒	☐ Antibodies
☒	☐ Eukaryotic cell lines
☒	☐ Palaeontology and archaeology
☒	☐ Animals and other organisms
☒	☐ Clinical data
☒	☐ Dual use research of concern

Methods

n/a	Involved in the study
☒	☐ ChIP-seq
☒	☐ Flow cytometry
☒	☐ MRI-based neuroimaging
